# Clinical characteristics and outcomes in leptomeningeal disease with or without brain metastasis: insights from an explorative data analysis of the Charité LMD registry

**DOI:** 10.1007/s11060-025-04937-x

**Published:** 2025-02-11

**Authors:** David Wasilewski, Chiara Eitner, Rober Ates, Selin Murad, Zoe Shaked, Julia Alexandra Steinle, Andreas Wetzel-Yalelis, Tarik Alp Sargut, Judith Rösler, Majd Abdulhamid Samman, Peter Truckenmüller, Robert Mertens, Daniel Kroneberg, Alexander Kowski, Helena Radbruch, David Capper, Felix Ehret, Siyer Roohani, Nikolaj Frost, Jawed Nawabi, Julia Onken, Maximilian Schlaak, Jens-Uwe Blohmer, Uwe Pelzer, Ulrich Keller, Jalid Sehouli, Peter Vajkoczy, Ulrich Keilholz, Martin Misch

**Affiliations:** 1 Department of Neurosurgery, Charité - Universitätsmedizin Berlin, Corporate Member of Freie Universität Berlin Und Hum-Boldt-Universität Zu Berlin, Berlin, Germany; 2 Charité - Universitätsmedizin Berlin, Corporate Member of Freie Universität Berlin Und Hum-Boldt-Universität Zu Berlin, Charité Comprehensive Cancer Center, Berlin, Germany; 3 German Cancer Consortium (DKTK), Partner Site Berlin, a partnership between DKFZ and Charité - Universitätsmedizin Berlin, Berlin, Germany; 4 Department of Gynecology, Charité, Universitätsmedizin Berlin, Germany, Corporate Member of Freie, Universität Berlin Und Humboldt-Universität Zu Berlin, Berlin, Germany; 5 Berlin Institute of Health at Charité - Universitätsmedizin Berlin, Berlin Institute of Health (BIH) Charité, Charitéplatz 1, 10117 Berlin, Germany; 6 Department of Neurology, Charité - Universitätsmedizin Berlin, Corporate Member of Freie Universität Berlin Und Humboldt-Universität Zu Berlin, Berlin, Germany; 7 Department of Neuropathology, Charité - Universitätsmedizin Berlin, Corporate Member of Freie Universität Berlin Und Humboldt-Universität Zu Berlin, Berlin, Germany; 8 Department of Radiation Oncology, Charité - Universitätsmedizin Berlin, Corporate Member of Freie Universität Berlin Und Humboldt-Universität Zu Berlin, Berlin, Germany; 9 Department of Infectious Diseases and Respiratory Medicine, Charité - Universitätsmedizin Berlin, Corporate Member of Freie Universität Berlin Und Humboldt-Universität Zu Berlin, Berlin, Germany; 10 Department of Neuroradiology, Charité - Universitätsmedizin Berlin, Corporate Member of Freie Universität Berlin Und Humboldt-Universität Zu Berlin, Berlin, Germany; 11 Department of Radiology, Charité - Universitätsmedizin Berlin, Corporate Member of Freie Universität Berlin Und Humboldt-Universität Zu Berlin, Berlin, Germany; 12 Department of Hematology, Oncology and Cancer Immunology, Charité - Universitätsmedizin Berlin, Corporate Member of Freie Universität Berlin Und Humboldt-Universität Zu Berlin, Berlin, Germany; 13 Department of Dermatology, Venerology and Allergology, Charité - Universitätsmedizin Berlin, Corporate Member of Freie Universität Berlin Und Humboldt-Universität Zu Berlin, Berlin, Germany

**Keywords:** Brain metastasis, Resection, Re-resection, Radiotherapy, Breast cancer, Melanoma, Lung cancer, Survival

## Abstract

**Introduction and objectives:**

Leptomeningeal disease (LMD) involves disseminating cancer cells to the leptomeninges and cerebrospinal fluid. The impact of intracranial parenchymal brain metastases and extracranial disease burden at LMD diagnosis remains unclear. This study evaluates these factors alongside local and systemic therapies before and after LMD diagnosis.

**Methods:**

A retrospective analysis was conducted on 188 patients diagnosed with LMD between 2011 and 2024. Data on demographics, imaging findings, and treatments were collected. Kaplan–Meier estimates were used for survival analysis, and independent prognostic factors were identified using a backward-stepwise Cox regression model.

**Results:**

Primary cancers included breast cancer (34.0%), non-small cell lung cancer (22.3%), and melanoma (14.4%). LMD was diagnosed via MRI in 56.4% of cases, cerebrospinal fluid (CSF) cytology in 2.7%, and both in 41.0%. Median overall survival was 2.8 months [95% CI: 2.4 – 3.7]. Independent prognostic factors for improved survival included male sex (HR: 0.61 [95% CI: 0.40 – 0.93], p = 0.020), absence of hydrocephalus at LMD diagnosis (HR: 0.42 [95% CI: 0.22 – 0.79], p = 0.007), and targeted therapy post-diagnosis (HR: 0.33 [95% CI: 0.20 – 0.55], p < 0.001). Two or more lines of systemic therapy before LMD diagnosis increased mortality risk (HR: 1.73 [95% CI: 1.16 – 2.59], p = 0.007). Lack of CNS parenchymal disease at LMD diagnosis also increased risk (HR: 0.51 [95% CI: 0.30 – 0.89], p = 0.017). Pre-diagnosis radiation therapy showed no survival benefit, while post-diagnosis radiation improved outcomes (HR: 0.47 [95% CI: 0.32 – 0.70], p < 0.001).

**Conclusion:**

Absence of hydrocephalus and use of targeted therapy post-diagnosis are favorable prognostic factors, while extensive prior systemic therapy and CNS parenchymal disease worsen outcomes. Tailored therapies addressing intracranial disease are crucial for improving survival in LMD patients.

**Supplementary Information:**

The online version contains supplementary material available at 10.1007/s11060-025-04937-x.

## Introduction

Leptomeningeal disease (LMD) also known as leptomeningeal metastasis (LMM) involves the spread of cancer cells into the leptomeninges and/or cerebrospinal fluid (CSF). It is generally considered a late-stage complication of metastatic malignancies associated with significant morbidity, such as neurological deficits, hydrocephalus (HCP), and a poor prognosis [1, 2]. As in brain metastasis (BrM) (i.e. parenchymal central nervous system (CNS) metastases), the most common underlying tumor entities are breast cancer, Non-small cell lung cancer (NSCLC), and melanoma, followed by gastrointestinal tumors such as gastric cancer [3, 4]. LMD as a CNS involvement is infrequent and may range from 2–12% of cases, whereas LMD may affect up to 37% of patients with BrM at later time points during their disease course typically following local pre-treatment, including BrM resection and stereotactic radiosurgery (SRS) [5–8]. Diagnosis in clinical practice varies and may involve one or more of the following diagnostic tests: cranial and spinal magnetic resonance imaging (MRI), CSF sampling, or biopsy in unclear cases [4–7]. Growing evidence indicates that the LMD incidence and the risk of disease relapse through LMD appear to be on the rise. This is partly explainable through longer median overall survival (OS) of cancer patients based on advancements in local and systemic therapies resulting in improved control of intracranial BrM burden and systemic disease, i.e., primary tumor and extracranial metastases (EcM) [3, 4, 6, 8]. Therapy for LMD may include local interventions such as neurosurgery (e.g., resection of space-occupying BrM, ventriculoperitoneal (VP) shunt or reservoir implantation) and radiotherapy (RTx) such as whole brain radiation (WBRT), or craniospinal axis irradiation (CSI) [3, 4, 8–10].

A multitude of retrospective studies with different subpopulations identified prognostic factors of patients with newly diagnosed LMD, for example, including tumor type, Karnofsky performance score (KPS), positive CSF cytology, type of LMD involvement (i.e. classic linear “sugarcoating” enhancement vs. nodular involvement), use of targeted therapy or receptor tyrosine kinase inhibitors (RTKIs) after LMD diagnosis, primary tumor control [3, 8, 10–18].

Despite the growing recognition, recent prospective trials in the context of LMD and retrospective studies reporting patient outcomes, comprehensive analyses of real-world data regarding the prognostic impact of intracranial BrM and extracranial disease burden before and at the time of LMD diagnosis, as well as the activity of the primary tumor, are scarce [19, 20]. This retrospective single-center study of a large cohort of LMD patients aims to characterize in detail the clinicopathological characteristics and local and systemic therapies before and after the diagnosis of LMD. Our objective is to provide insights into the prognostic factors at the time of LMD diagnosis, focusing on the relevance of intracranial BrM and extracranial metastasis burden in newly diagnosed LMD patients.

## Methods

### Patient cohort

Patients with known underlying solid tumor malignancy and evidence of leptomeningeal spread (LMD) and presence of BrM before the diagnosis of LMD or at diagnosis of LMD were included in this study with signs of LMD via cranial MRI (cMRI) and/or spinal MRI (sMRI) and/or positive lumbar puncture with detection of tumor cells via CSF analysis (Supplementary Fig. 1A, B). Patients with hematologic malignancy or primary central nervous system malignancy were excluded. Patients were retrospectively and, in part, prospectively identified and treated at a tertiary care center between January 2011 and April 2024. All eligible cases were included in this study. Clinical variables of interest were based on previous studies in the field. They involved demographics, radiological characteristics, pathology-related characteristics at baseline (i.e., diagnosis date of LMD), previous treatment modalities before a diagnosis of LMD, and treatment modalities after diagnosis of LMD and were extracted from patient records. Stratification according to classic LMD and nodular LMD was based on the classification of Turner et al. [13]. Radiologic tumor assessment related to intracranial disease, or the central nervous system and EcM burden was determined based on cMRI with or without sMRI scan at the time of LMD diagnosis and a CT staging 2 months before or after LMD diagnosis following the discretion of the treating physician and based on retrospective patient chart reviews without central confirmation according to standardized response evaluation criteria response assessment in neuro-oncology (RANO) criteria and response evaluation criteria in solid tumors (RECIST) criteria (version 1.1), respectively. Tumor response on cMRI or CT staging was classified into complete response (CR), partial response (PR), stable disease (SD), or progressive disease (PD) for all included patients. Information on molecular pathology was extracted from institutional neuropathology or pathology reports on either tumor tissue or extracranial tumor tissue. Lack of CNS parenchymal disease or CNS status is henceforth defined as patients without a previous history of BrM before LMD diagnosis and lack of presence of BrM at the time of LMD diagnosis.

### Statistical analysis

Descriptive statistics were performed to summarize the presented patient cohort and associated clinical, histopathological, radiological, and treatment-related patient features. All statistical analyses were performed using R software (version 4.0.3) (R Foundation) to compute statistics, including frequencies, means, and SDs, to characterize the cohort. We used the gtsummary package (R Foundation) to describe tabular data of the patient cohort, including categorical and numerical variables. Comparisons between groups were made using the chi-squared or Fisher’s exact test (categorical variables) and Wilcoxon rank sum test (continuous variables) with addition of multiple comparison adjustment based in the Benjamini-Hochberg multiple testing procedure. Median OS was estimated by Kaplan–Meier (KM) analysis with 95% CI bands being displayed in gray; plotting was performed using the survival and survminer packages (R Foundation). We used the bootstrap resampling technique to calculate the median follow-up time and its 95% confidence interval (CI). The dataset included diagnosis dates and the latest follow-up dates, allowing for the calculation of follow-up durations. The boot package in R was used to perform 1,000 bootstrap resamples of the follow-up times, computing the median for each resample. The 95% Cis were derived using the percentile method, identifying the 2.5th and 97.5th percentiles of the bootstrap distribution. A multivariable Cox regression model for OS was computed using preselected clinical variables of interest deemed associated with the respective outcome. We used the survival and MASS packages for fitting the Cox proportional hazards model and conducting stepwise regression, in which a backward stepwise elimination method of sequential variable exclusion with the highest p-value variable being excluded at each step until only those with p < 0.15 were left, and where values of 0.05 < p < 0.15 ultimately were included in the final model. The ggforest function from the survminer package was utilized to visualize the results of the final model. Further R packages included viridis, ggsankey, dplyr, tidyverse. Data collection was performed with Excel version 14.3.9 (Microsoft). A P value < 0.05 was considered significant, with P values being 2-sided. R code and raw data will be made available on request.

## Results

### Baseline patient characteristics

**Fig. 1:**
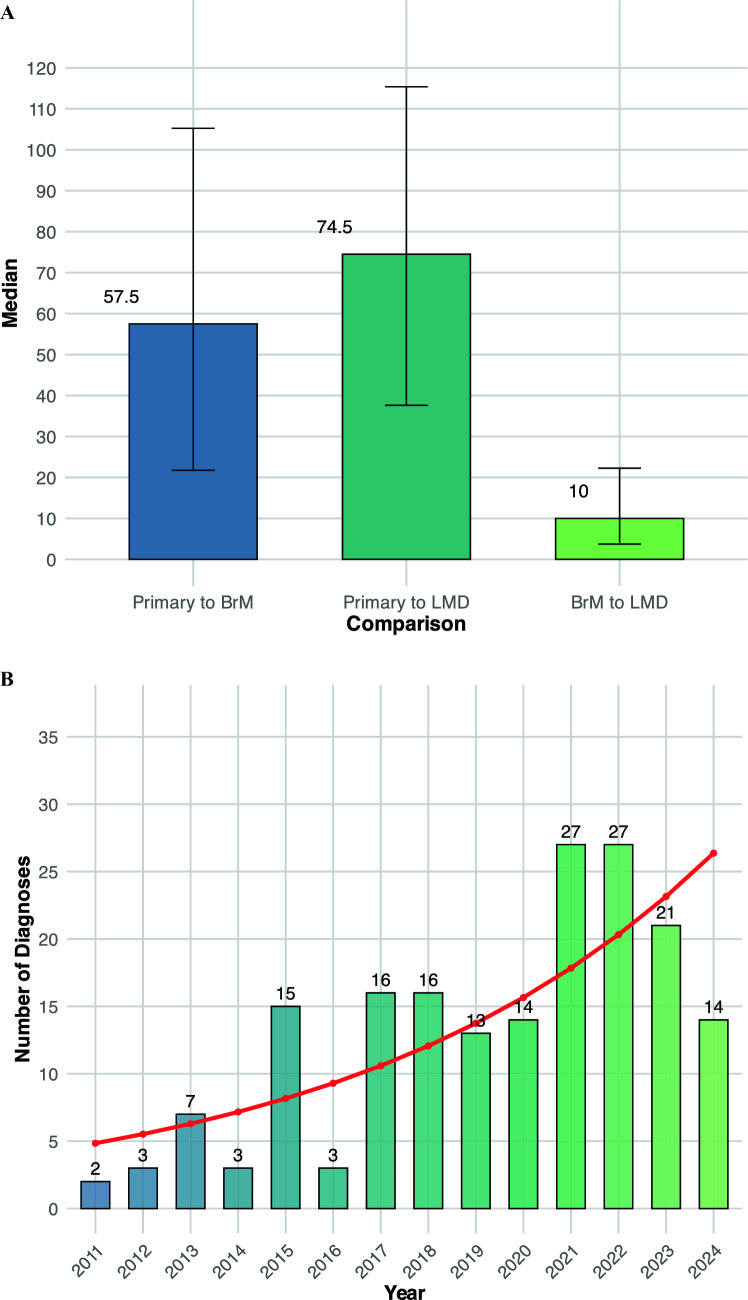

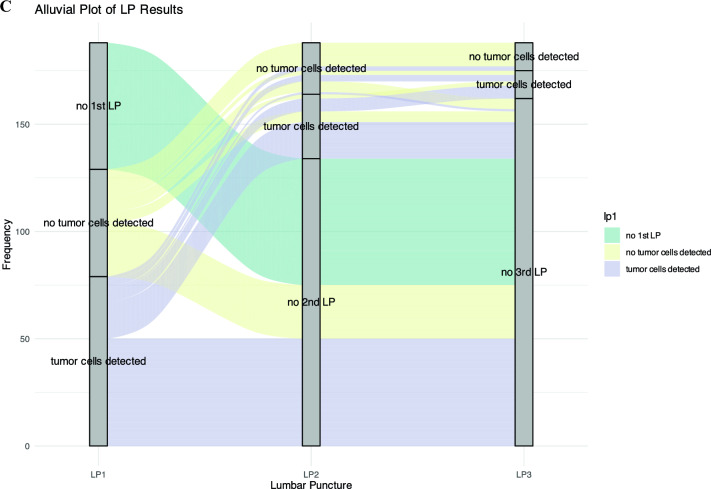
Progression Timeline and Diagnostic Trends in Leptomeningeal Disease. A bar graph depicting the median time intervals for transitions between disease states in patients. It shows that the median time from primary cancer diagnosis to the development of BrM is 57.5 months, with a 95% confidence interval (CI) of 21.8 to 105.3 months. From primary diagnosis to leptomeningeal disease (LMD) diagnosis, the median interval is 74.5 months, with a CI of 37.6 to 115.4 months. Additionally, the median time from developing brain metastases to LMD diagnosis is 10.0 months, with a CI of 3.7 to 22.3 months (**A**). Histogram overlayed with a regression plot depicting the annual number of LMD diagnoses from 2011 to 2024 and the trend indicating a statistically significant annual increase in LMD diagnoses by approximately 13.9%, with a coefficient for the diagnosis year of 0.130 (SE = 0.020, z = 6.53, p < 0.001). Figure 1C features an alluvial diagram that visualizes the frequency and outcomes of lumbar punctures (LP) across multiple attempts (LP1, LP2, LP3). The diagram tracks the detection or absence of tumor cells at each LP attempt, effectively illustrating the flow of patients and the changes in test results across successive LPs, thereby highlighting the diagnostic process over time

One hundred and eighty-eight patients diagnosed with LMD with or without a history of parenchymal intracranial BrM were included (Supplementary Fig. 1, Fig. 1). The median time from primary diagnosis to BrM was 57.5 months [95%CI: 21.8—105.3], the median time from primary diagnosis to LMD was 74.5 months [95%CI: 37.6—115.4], and the median time from BrM to LMD was 10.0 months [95% CI: 3.7—22.3] Fig. 1A). Median follow-up from diagnosis of LMD was 69 days [95% CI: 53.5 – 82]. Median OS of the whole cohort was 2.8 months [95% CI: 2.4 – 3.7] with 149 deaths (79.3%) during the follow-up period. LMD diagnosis data from 2011 to 2024 showed a significant upward trend in annual diagnoses, with a coefficient for diagnosis year of 0.130 (SE = 0.020, z = 6.53, p < 0.001), indicating an approximate 13.9% annual increase in LMD diagnoses highlighting a significant rise in LMD case identification over time (Fig. 1B).

Patient characteristics are summarized in the supplementary part of this work (Supplementary Table 1A-D, Supplementary Table 2A-C).

Further baseline features, pre-LMD treatment characteristics as well as treatment characteristics after LMD diagnosis grouped according to the three most common tumor entities (breast cancer, NSCLC, melanoma vs. other) are summarized in the supplementary part (Supplementary Table 2A-C). LMD was diagnosed via MRI in 106 patients (56.4%), cerebrospinal fluid cytology only in 5 patients (2.7%), and both methods in 77 patients (41.0%). Data on serial lumbar punctures show that on the first LP (lp1), 79 out of 188 patients (42.0%) tested positive for tumor cells, with breast cancer being the tumor type with the most frequent positive detection rate regarding the first LP (Fig. 1C). The majority of patients (134) did not receive a second puncture. Of the patients receiving a second LP. Of the patients receiving a second LP (lp2), 30 patients (55.6%) were positive, including 7 who tested negative in lp1. By the third LP (lp3), the positive detection rate further decreased to 13 patients (50.0%), including 2 who tested negative up to lp2 (Figure 1C). Of 26 patients who received all three lumbar punctures, 13 tested positive (50.0%). 

### Association of clinical factors with survival after LMD diagnosis

The median OS of the whole cohort was 2.8 months [95% CI: 2.4 – 3.7] (Supplementary Fig. 2A). When tumor entities were grouped according to the most common tumor types, i.e., breast cancer, NSCLC, melanoma, and “other”, survival times varied according to the tumor group. Breast cancer patients had the highest median OS time of 4.9 months [95% CI: 3.1 – 8.6], those with NSCLC had 2.4 months [95% CI: 1.7 – 3.2], melanoma patients had a notably shorter median OS time of 1.6 months [95% CI: 0.8 – 6.9], and patients categorized as ‘other’ had a median OS time of 2.8 months [95% CI: 1.9 – 4.8] (Supplementary Fig. 2B). Common clinical variables investigated in LMD patients, such as age, KPS, presence of other underlying chronic conditions such as cardiovascular disease, and pattern of LMD, were not associated with OS (Supplementary Fig. 2C-G). Interestingly, the pattern of LMD did not seem to affect the clinical course (Supplementary Fig. 2F-G), but patients with the presence of HCP were associated with a significantly worse prognosis (Supplementary Fig. 2H).

Analysis of the impact of history of BrM and EcM before LMD diagnosis, presence of intracranial parenchymal metastases and EcM at the time of LMD diagnosis as well as activity of intracranial metastases and EcM in the context of LMD diagnosis on survival showed that none of these factors associated with OS in our cohort using KM survival analysis (Supplementary Fig. 3A-E; Supplementary Fig. 4A-E). Additionally, the activity of the primary tumor was not associated with OS (Supplementary Fig. 4F).

**Fig. 2:**
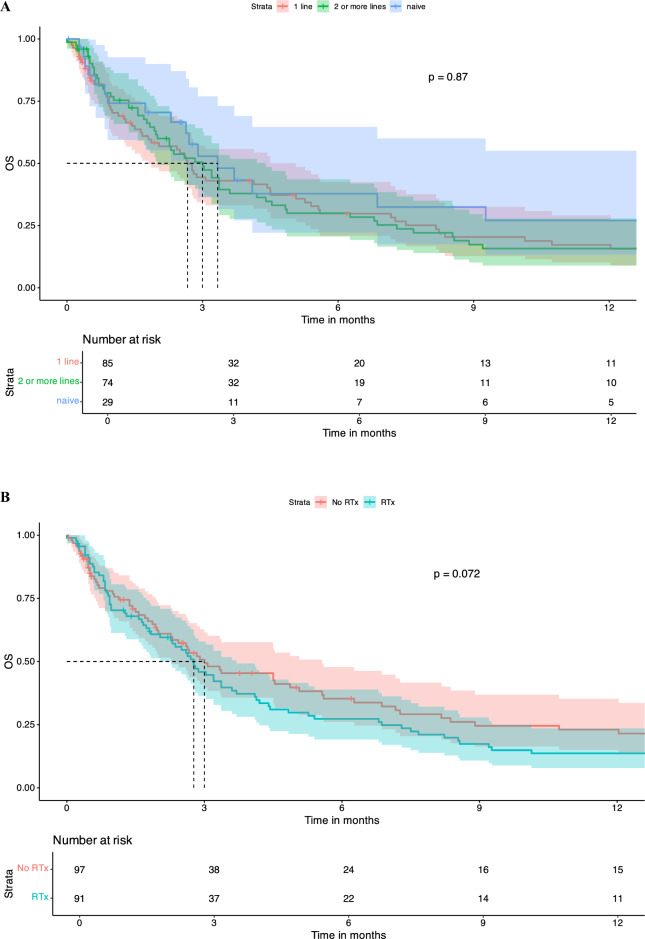

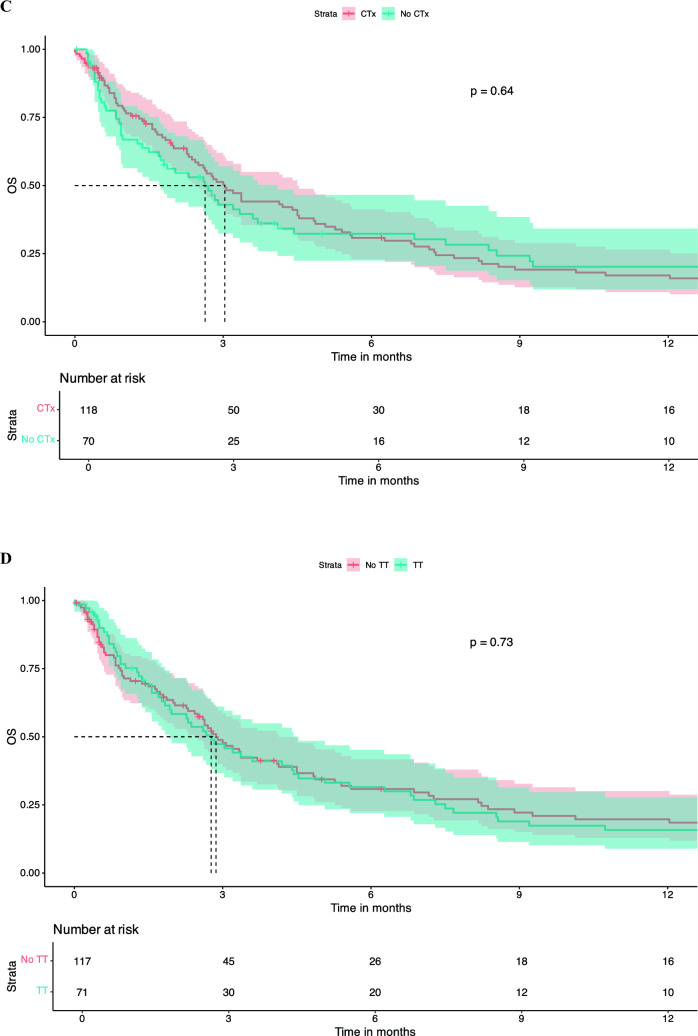

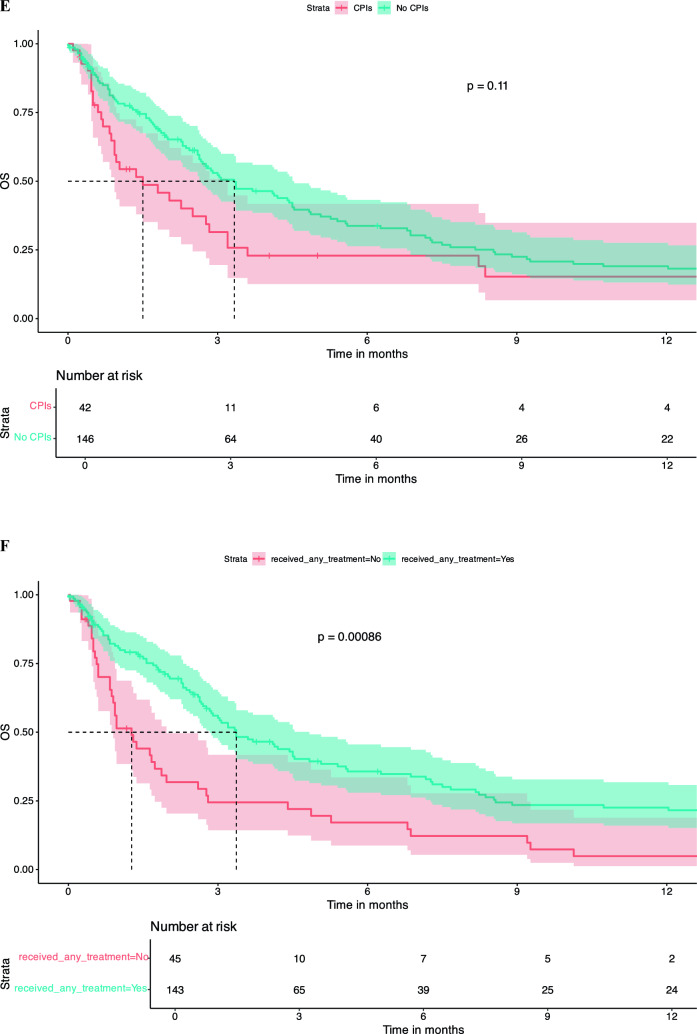

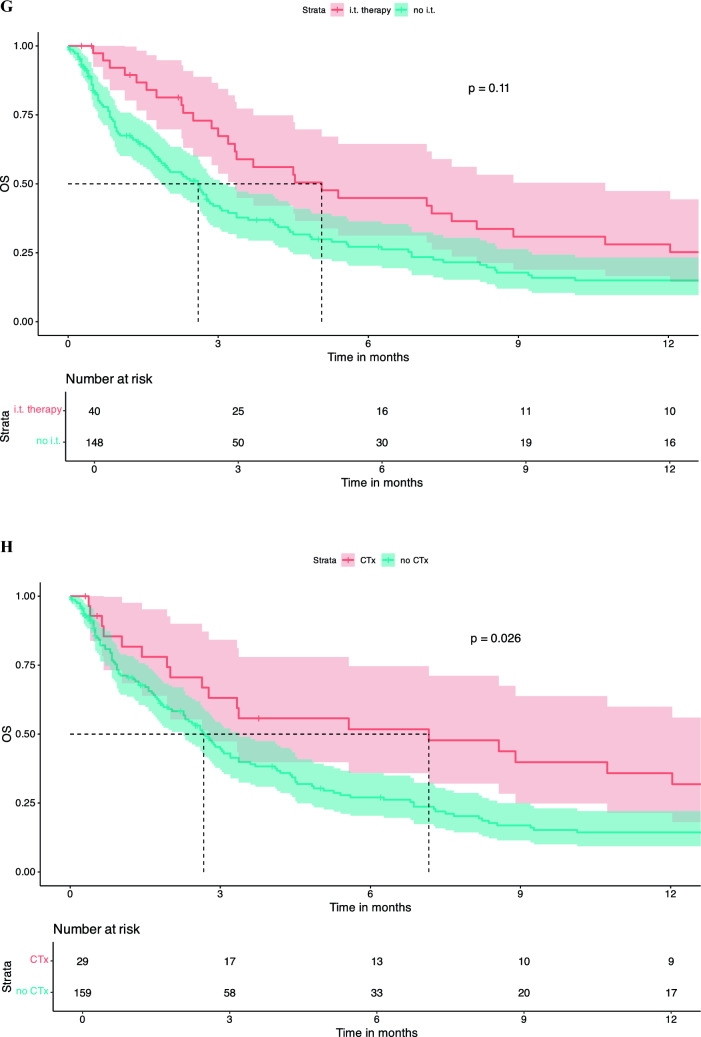

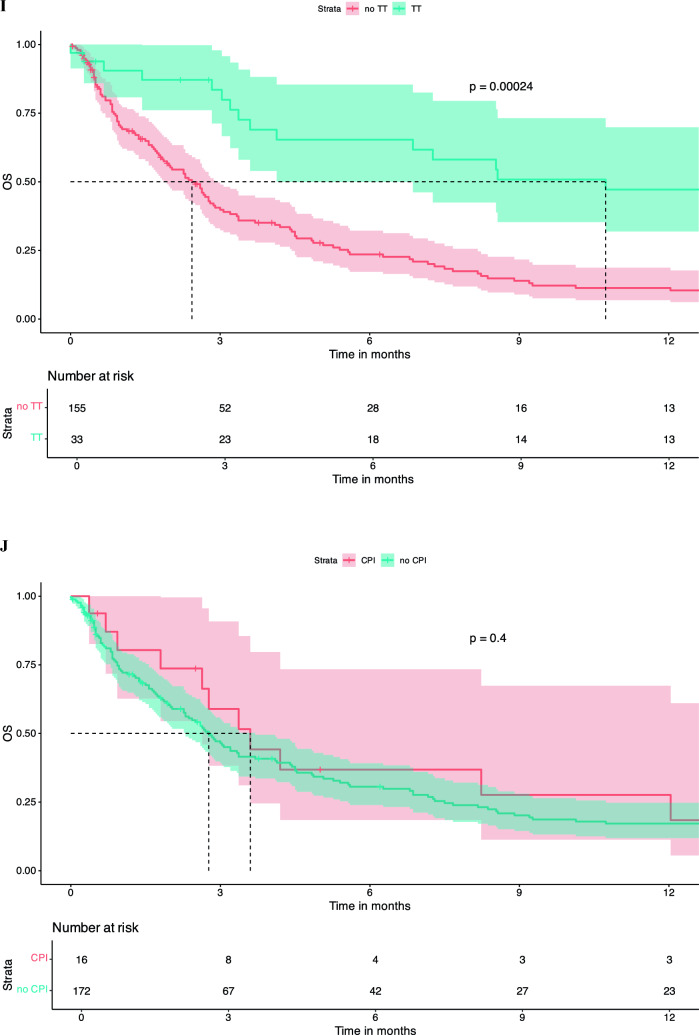

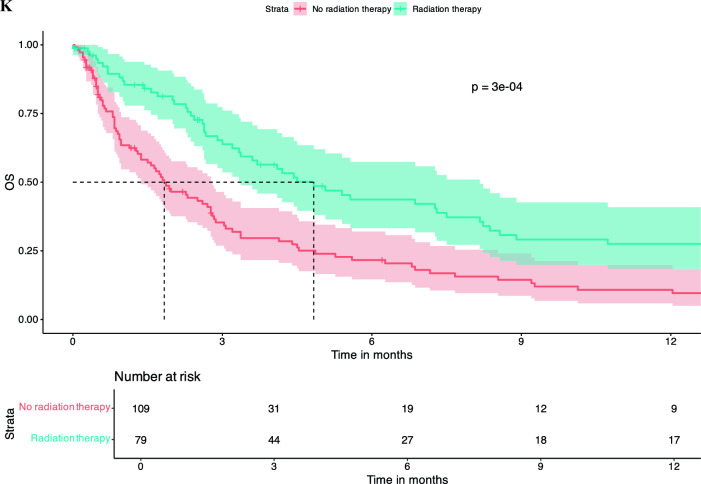
Pre-LMD treatment characteristics and KM estimates for OS. Number of Therapy Lines Before LMD Diagnosis: Patients who underwent one line of systemic therapy before LMD diagnosis had a median OS of 2.3 months [95% CI: 1.8 – 5.1]. Those who had two or more lines of systemic pre-treatment had a median OS of 3.0 months [95% CI: 2.0 – 4.4], and treatment-naive patients had a median OS of 3.3 months [95% CI: 2.6 – 14.0], p = 0.98 (**A**). When patients were dichotomized into those who had any form of RTx before LMD diagnosis versus those who had no RTx, the median OS for those who did not receive RTx was 3.0 months [95% CI: 2.3 – 5.6], and for those who received RTx, the median OS was 2.8 months [95% CI: 2.3 – 3.7], p = 0.76 (**B**). Chemotherapy Before LMD Diagnosis: Patients who received chemotherapy before LMD diagnosis had a median OS of 3.0 months [95% CI: 2.4 – 4.5]. Those not receiving chemotherapy had a median OS of 2.63 months [95% CI: 1.7 – 4.1], p = 0.91 (**C**). Targeted Therapy Before LMD Diagnosis: Patients who did not receive targeted therapy before their LMD diagnosis had a median OS of 2.9 months [95% CI: 2.4 – 4.1], whereas those who received targeted therapy had a median OS of 2.8 months [95% CI: 1.9 – 4.5], p = 0.98 (**D**). Immunotherapy Before LMD Diagnosis: Patients who received immunotherapy (checkpoint inhibitors) before their LMD diagnosis had a median OS of 1.5 months [95% CI: 0.9 – 3.2]. Those who did not receive CPI had a significantly higher median OS of 3.3 months [95% CI: 2.7 – 4.5], p = 0.21 (**E**). KM survival curves indicate that patients who received any treatment following an LMD diagnosis had a median OS of 2.8 months [95% CI: 1.9 – 3.9], whereas those who did not receive any treatment post-LMD had a median OS of 1.67 months [95% CI: 0.5 – 3.0], p = 0.008 (**F**). KM survival curves comparing OS between patients who received intrathecal methotrexate (i.t.) treatment after LMD diagnosis and those who did not. Median OS for the i.t. therapy group was 5.1 months [95% CI: 3.3 – 10.7], compared to 2.6 months [95% CI: 1.9 – 3.1] for the no i.t. therapy group, p = 0.11 (**G**). KM survival curves comparing OS between patients receiving chemotherapy (CTx) after LMD diagnosis and those who did not. median OS for patients who received CTx was 7.2 months [95% CI: 2.8 – 23.2], compared to 2.7 months [95% CI: 2.3 – 3.4] for those who did not receive CTx (p = 0.026) (**H**). KM curves assessing OS differences between patients receiving targeted therapy after LMD diagnosis versus those who did not. Patients who received targeted therapy exhibited a significantly higher median OS of 10.7 months [95% CI: 6.9 – 46.2], compared to 2.4 months [95% CI: 1.9 – 2.9] for patients without targeted therapy (p = 0.00024) (**I**) KM analysis for patients treated with immunotherapy (CPI) after LMD diagnosis versus those who did not receive CPI. The median OS for patients receiving CPI was 3.6 months [95% CI: 2.6 – NA], compared to 2.88 months [95% CI: 2.3 – 3.7] for those without CPI treatment (p = 0.4) (**J**). Kaplan–Meier survival curves comparing OS between patients who received radiation therapy post-LMD diagnosis vs those patients without post-LMD radiation therapy. Patients receiving radiation therapy exhibited a median OS of 4.8 months [95% CI: 3.4 – 8.2], compared to 1.8 months [95% CI: 1.4 – 2.8] for patients without radiation therapy (p = 0.0003) (**K**)

There was no significant difference in OS between patients who were treatment naïve in terms of systemic therapy at the time of LMD diagnosis vs. pre-treated patients (dichotomized into naïve vs. 1 line vs. ≥ 2 lines). Number of treatment lines was not associated with OS in KM survival analysis (Fig. 2A). Similar findings were observed regarding pre-treatment with CNS-RTx or systemic pre-treatment status (Fig. 2B-E).

Any treatment, either systemic treatment and/or local treatment post-LMD diagnosis was associated with an increased survival median OS of 3.37 months [95% CI: 2.8 – 4.8] vs. no treatment post-LMD diagnosis 1.3 months [95% CI: 0.9 – 2.0], p = 0.00086 (Fig. 2F). Post-LMD diagnosis patients that received intrathecal (i.t.) methotrexate (MTX) had a median OSs of 5.1 months [95% CI: 3.3 – 10.7] compared to 2.6 months [95% CI: 1.9 – 3.1] for those who did not receive this treatment (p = 0.11) (Fig. 2G**).** No difference between i.t. MTX via repetitive LP vs. i.t. MTX via a reservoir was noted (data not shown). Furthermore, LMD patients who received systemic chemotherapy had a significantly higher median OS of 7.2 months [95% CI: 2.8 – 23.2], whereas those who did not receive chemotherapy had a median OS of 2.7 months [95% CI: 2.3 – 3.4] (p = 0.026) (Fig. 2H). Similarly, patients receiving targeted therapies (TT), including monoclonal antibodies (e.g., trastuzumab in breast cancer or bevacizumab in Her2/neu negative breast cancer) or small molecule inhibitors as in the case of melanoma (e.g., MEK and/or BRAF inhibitors as well as RTKIs against EGFR) showed a significantly longer median OS compared to patients without TT after LMD (10.7 months [95% CI: 6.9 – 46.2] vs. 2.4 months [95% CI: 1.9 – 2.9], p = 0.00024), whereas use of CPI was not associated with increased OS (Fig. 2I and J). Patients treated with RTx after LMD diagnosis showed a significantly longer median OS of 4.83 months [95% CI: 3.8 – 8.2] vs. patients without any RTx after LMD diagnosis with a median OS of 1.8 months [1.4 – 2.8], p = 0.0003 (Fig. 2K).

### Independent prognostic factors

**Fig. 3:**
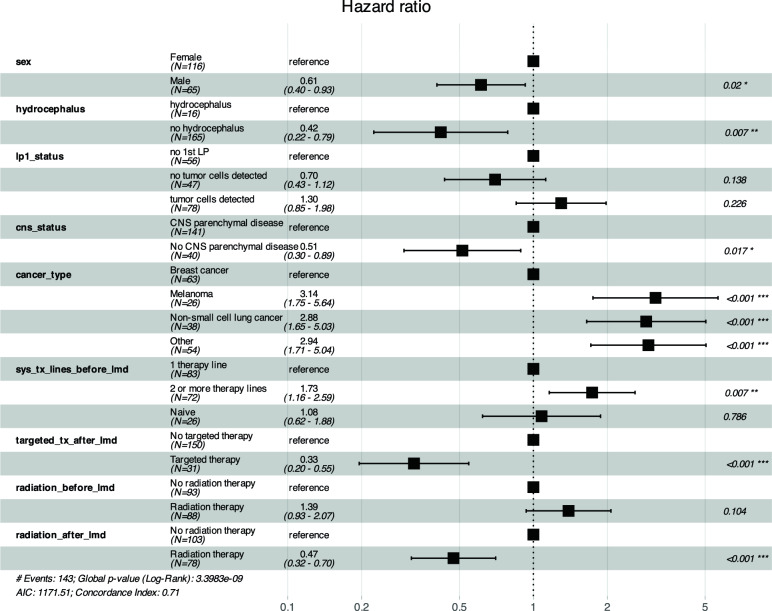
Stepwise Cox regression analysis for OS. The forest plot illustrates the hazard ratios (HR) and associated 95% confidence intervals of clinical variables included in the final Cox proportional hazards model, which was developed using a backward stepwise selection procedure. This model assesses the association of individual variables with median OS  in patients with leptomeningeal disease (LMD). The variables that remained significant in the model include sex (“sex”), the presence of HCP at the time of LMD diagnosis (“hydrocephalus”), lumbar puncture (LP) status with no tumor cells detected (“lp1_status_no_tumor_cells_detected”) and with tumor cells detected (“lp1_status_tumor_cells_detected”), central nervous system (CNS) disease status (“cns_status”), cancer type (melanoma, NSCLC other; “cancer_type”), systemic therapy lines before LMD diagnosis (2 or more therapy lines, naive; “sys_tx_lines_before_lmd”), targeted therapy after LMD diagnosis (“targeted_tx_after_lmd”), radiation therapy before LMD diagnosis (“radiation_before_lmd”), and radiation therapy after LMD diagnosis (“radiation_after_lmd”). The forest plot confirms the robustness of the stepwise model, where non-significant variables were iteratively removed based on the Akaike Information Criterion (AIC), which balances the goodness of fit and model complexity, ensuring the most parsimonious model without overfitting, thereby providing reliable hazard ratio estimates for the variables that remain. The horizontal lines that do not cross the HR of 1 (the vertical line) indicate statistically significant effects. Variables with confidence intervals that span across 1 suggest no significant effect on survival. This technical visualization facilitates the interpretation of complex survival data, highlighting key factors that influence patient outcomes in LMD. The high concordance index (C = 0.71, SE = 0.022) indicates good predictive accuracy of the model, demonstrating that the selected variables provide a strong explanatory power for the survival data in this patient cohort. This forest plot, therefore, serves as a crucial tool for understanding the impact of clinical and treatment-related factors on the prognosis of LMD patients. The discrepancy between the total number of events from the total number of deaths in the Cox model arises because patients with missing data in any of the model’s variables were excluded from the analysis (complete case analysis)

To identify independent prognostic factors for OS, backward stepwise multivariate Cox proportional hazards regression for baseline variables at LMD diagnosis was performed, showing that lack of HCP as well as lack of history of parenchymal CNS metastases and lack of parenchymal CNS metastases at the time of LMD diagnosis (summarized as cns_status) were associated with decreased risk of death: HR: 0.42 [95% CI: 0.22–0.79], p = 0.007 and HR: 0.51 [95% CI: 0.30–0.89], p = 0.017, respectively. Melanoma (HR: 3.14 [95% CI: 1.75–5.64], p < 0.001), NSCLC (HR: 2.88, [95% CI: 1.65–5.03], p < 0.001) and other entities (HR: 2.94, [95% CI: 1.71–5.04], p < 0.001) were associated with increased mortality risk. Administration of TT and radiation therapy after LMD diagnosis were associated with a decreased risk of death (Fig. 3).

### Comparison of patient characteristics grouped according to involvement of CNS parenchymal disease

Of the total cohort of 188 patients, 145 had CNS parenchymal disease, and 43 had no CNS parenchymal disease. Notably, a larger but non-significant percentage of patients with CNS parenchymal disease had a lower KPS (≤ 70%) at the time of LMD diagnosis (59% vs. 77%, p = 0.073). Similarly, symptoms like headache (39.3% vs. 48.8%), as well as nausea (26.2% vs. 11.6%), and vomiting (15.9% vs. 2.3% were more common in patients with CNS parenchymal disease, however without any significant difference. There was a significant difference in the modality of LMD diagnosis: MRI was the primary tool for LMD diagnosis in the case of patients with CNS parenchymal disease (62.8% vs. 34.9%, p = 0.013), whereas in the case of patients without CNS parenchymal disease CSF sampling together with MRI was more frequently performed than in patients with CNS parenchymal disease (60.5% vs. 35.2%, p = 0.013) (Table 1, 2). Other entities (non-breast cancer, non-melanoma, non-NSCLC) were more significantly more frequent in the non-parenchymal disease group with 34 patients (23.4%) vs. 21 patients (48.8%) (p=0.044). Interestingly, presence of extracranial metastases at the time of LMD diagnosis was more frequently observed in patients without any CNS parenchymal disease with 34 patients (79.1%) vs. 81 patients (55.9%) (p=0.023). Regarding systemic therapies, 84.8% of patients with CNS parenchymal disease received systemic treatment before LMD diagnosis, with chemotherapy being the most common (38.6% in CNS parenchymal disease vs. 31.0% in non-CNS parenchymal disease). Targeted therapies and checkpoint inhibitors before LMD diagnosis were also more prevalent in the CNS group (64.1% vs. 55.8% and 75.9% vs. 83.7%, respectively) (Table 2). Following LMD diagnosis, intrathecal therapy was administered to a higher percentage of patients in the non-CNS parenchymal disease group via both lumbar punctures (16.3% vs. 11.0%) and reservoirs (14.0% vs. 7.6%). Post-diagnosis chemotherapy was more frequent in non-CNS parenchymal disease patients, with a diverse range of agents used. However, overall, the proportion of patients receiving classic chemotherapy was relatively low in both groups (12.4% for CNS parenchymal disease and 25.6% for non-CNS parenchymal disease). Chemotherapy, TT and checkpoint inhibitors were similarly distributed between groups (Table 3). Local therapies after diagnosis showed significant variability, with BrM resection and later radiation therapy being more common in CNS parenchymal disease patients (37.2% vs. none in non-CNS). Radiation therapy, particularly WBRT, was more frequently employed in CNS patients after LMD diagnosis (30.3% vs. 27.9%) (Table 3).

**Table 1:** General patient characteristics

Variable	CNS parenchymal disease, N = 145	No CNS parenchymal disease, N = 43	p-value^*1*^	q-value^*2*^
Age, Median (IQR)	57.3 (47.7 – 66.8)	55.2 (49.6 – 66.7)	0.92	0.92
Gender, n (%)			0.21	0.31
Female	96 (66.2)	24 (55.8)		
Male	49 (33.8)	19 (44.2)		
KPS group at the time of LMD diagnosis, n (%)			0.031	0.073
70% or less	85 (59)	33 (77)		
More than 70%	60 (41)	10 (23)		
Presence of other underlying chronic diseases, n (%)			0.18	0.30
No other underlying chronic diseases	64 (44.1)	14 (32.6)		
Other underlying chronic diseases	81 (55.9)	29 (67.4)		
Headache present at diagnosis of LMD, n (%)	57 (39.3)	21 (48.8)	0.27	0.36
Nausea present at diagnosis of LMD, n (%)	38 (26.2)	5 (11.6)	0.046	0.10
Vomiting present at diagnosis of LMD, n (%)	23 (15.9)	1 (2.3)	0.019	0.051
Vertigo present at diagnosis of LMD, n (%)	30 (20.7)	12 (27.9)	0.32	0.41
Visual deficits present at diagnosis of LMD, n (%)	20 (13.8)	8 (18.6)	0.44	0.54
Seizures present at diagnosis of LMD, n (%)	13 (9.0)	1 (2.3)	0.20	0.31
Decrease in clinical performance present at diagnosis of LMD, n (%)	74 (51.0)	23 (53.5)	0.78	0.88
Cranial nerve deficits present at diagnosis of LMD, n (%)	34 (23.4)	15 (34.9)	0.13	0.25
LMD in the context of brain metastasis resection, n (%)			< 0.001	< 0.001
LMD at the time of brain metastasis resection	20 (13.8)	1 (2.3)		
No LMD before brain metastasis resection	75 (51.7)	0 (0.0)		
Not applicable (no brain metastasis resection performed)	50 (34.5)	42 (97.7)		
Modality of LMD Diagnosis, n (%)			0.003	0.013
LMD diagnosis based solely on MRI	91 (62.8)	15 (34.9)		
LMD diagnosis based solely on CSF sampling	3 (2.1)	2 (4.7)		
LMD diagnosis based on MRI and CSF sampling	51 (35.2)	26 (60.5)		
Dural biopsy	0 (0.0)	0 (0.0)		
Dominant pattern of LMD, n (%)			0.073	0.15
cLMD	85 (60.3)	32 (80.0)		
Mixed	19 (13.5)	2 (5.0)		
nLMD	37 (26.2)	6 (15.0)		
Unknown	4	3		
Presence of hydrocephalus at time of LMD diagnosis, n (%)			0.89	0.92
Hydrocephalus	18 (12.4)	5 (11.6)		
No hydrocephalus	127 (87.6)	38 (88.4)		
EANO-ESMO subtype, n (%)				
Type IA	43 (30)	24 (55.8)		
Type IB	12 (8.3)	3 (7.0)		
Type IC	7 (4.8)	1 (2.3)		
Type ID	0 (0)	1 (2.3)		
Type IIA	43 (30)	9 (21.0)		
Type IIB	25 (17)	3 (7.0)		
Type IIC	11 (7.6)	0 (0.0)		
Type IID	4 (2.8)	2 (4.7)		
History intracranial metastases, n (%)			< 0.001	< 0.001
No history of parenchymal brain metastases before LMD diagnosis	26 (17.9)	43 (100.0)		
History of parenchymal brain metastases before LMD diagnosis	119 (82.1)	0 (0.0)		
Parenchymal metastasis at the time of LMD diagnosis, n (%)			< 0.001	< 0.001
No parenchymal metastasis present at the time of LMD diagnosis	21 (14.5)	43 (100.0)		
Parenchymal metastasis present at the time of LMD diagnosis	124 (85.5)	0 (0.0)		
Activity of intracranial parenchymal diseases at the time of LMD diagnosis, n (%)			< 0.001	< 0.001
Not applicable (no parenchymal intracranial disease)	0 (0.0)	43 (100.0)		
PD	103 (71.0)	0 (0.0)		
PR	2 (1.4)	0 (0.0)		
SD	40 (27.6)	0 (0.0)		
Activity of primary tumor at the time of LMD diagnosis, n (%)			0.84	0.91
Active primary tumor	19 (13.1)	7 (16.3)		
No active primary tumor	123 (84.8)	35 (81.4)		
Not applicable (CUP)	3 (2.1)	1 (2.3)		
Extracranial metastases before LMD diagnosis, n (%)			0.018	0.051
No history of extracranial metastases before LMD diagnosis	51 (35.2)	7 (16.3)		
History of extracranial metastases before LMD diagnosis	94 (64.8)	36 (83.7)		
Extracranial metastases at the time of LMD diagnosis, n (%)			0.006	0.023
No extracranial metastasis present at the time of LMD diagnosis	64 (44.1)	9 (20.9)		
Extracranial metastasis present at the time of LMD diagnosis	81 (55.9)	34 (79.1)		
Activity of extracranial metastatic diseases at the time of LMD diagnosis, n (%)			0.20	0.31
Not applicable (no extracranial metastases)	45 (31.0)	7 (16.3)		
PD	42 (29.0)	18 (41.9)		
PR	9 (6.2)	3 (7.0)		
SD	49 (33.8)	15 (34.9)		
Entity, n (%)			0.014	0.044
Breast cancer	52 (35.9)	12 (27.9)		
Melanoma	23 (15.9)	4 (9.3)		
Non-small cell lung cancer	36 (24.8)	6 (14.0)		
Other	34 (23.4)	21 (48.8)		

General patient characteristics, including clinical characteristics and imaging-related patient features grouped according to the lack of brain metastasis in previous patient history and lack of brain metastasis at the time of LMD diagnosis vs. brain metastasis in the previous patient history as well as the presence of brain metastasis at the time of LMD diagnosis (CNS status)

Classic LMD (cLMD), nodular LMD (nLMD), LMD was classified into Type A: LM with typical linear MRI abnormalities; type B: LM with nodular disease; type C: LM with both linear and nodular disease; type D: LM without MRI abnormalities (except hydrocephalus) according to Le Rhun et and colleagues; Progressive disease (PD), partial response (PR), stable disease (SD) according to response evaluation criteria response assessment in neuro-oncology (RANO) criteria and response evaluation criteria in solid tumours (RECIST) criteria (version 1.1) for intracranial parenchymal metastasis and extracranial metastasis, respectively

^*1*^ Wilcoxon rank sum test; Pearson’s Chi-squared test; Fisher’s exact test

^*2*^ Benjamini & Hochberg correction for multiple testing

**Table 2:** Treatment-related characteristics before LMD diagnosis

Variable	CNS parenchymal disease, N = 145	No CNS parenchymal disease, N = 43	p-value^*1*^	q-value^*2*^
History of intracranial metastases, n (%)	119 (82)	0 (0)	< 0.001	< 0.001
Type of Neurosurgical Intervention, n (%)				
No neurosurgical intervention	38 (26.2)	32.0 (74.4)		
Resection	74 (51.0)	0.0 (0.0)		
Resection and later reservoir placement	10 (6.9)	0 (0.0)		
Resection and later VP shunt placement	3 (2.1)	0 (0.0)		
Resection and reservoir placement	3 (2.1)	0 (0.0)		
Resection and VP shunt placement	4 (2.8)	0 (0.0)		
Resection and VP shunt placement and later reservoir placement	1 (0.7)	0 (0.0)		
Reservoir placement	4 (2.8)	8 (18.6)		
Reservoir placement and VP shunt placement	1 (0.7)	0 (0.0)		
VP shunt placement	6 (4.1)	3 (7.0)		
VP shunt placement and later reservoir placement	1 (0.7)	0 (0.0)		
Presence of Implanted Reservoir, n (%)	21 (14.5)	8 (18.6)	0.51	0.57
Timing of Neurosurgical Resection Relative to LMD Diagnosis, n (%)			< 0.001	< 0.001
LMD at the time of brain metastasis resection	20 (13.8)	0.0 (0.0)		
No LMD before brain metastasis resection	75 (51.7)	0 (0.0)		
Not applicable (no brain metastasis resection performed)	50 (34.5)	43.0 (100.0)		
Number of Brain Metastasis Resections, n (%)			< 0.001	< 0.001
No neurosurgical brain metastasis resection	50 (34.5)	43.0 (100.0)		
One	61 (42.1)	0.0 (0.0)		
Two	28 (19.3)	0 (0.0)		
Three	1 (0.7)	0 (0.0)		
Four	4 (2.8)	0 (0.0)		
Five	1 (0.7)	0 (0.0)		
Line of Systemic Treatment Before LMD Diagnosis, n (%)			0.16	0.20
No systemic therapy (before LMD diagnosis)	22 (15.2)	7 (16.3)		
1 therapy line	69 (47.6)	16 (37.2)		
2 therapy lines	24 (16.6)	9 (20.9)		
3 therapy lines	18 (12.4)	3 (7.0)		
4 or more therapy lines	12 (8.3)	8 (18.6)		
Systemic Treatment Before LMD Diagnosis, n (%)			0.59	0.59
No systemic treatment before LMD diagnosis	22 (15.2)	8 (18.6)		
Systemic treatment before LMD diagnosis	123 (84.8)	35 (81.4)		
Chemotherapy Before LMD Diagnosis, n (%)				
No chemotherapy	56 (38.6)	13 (31.0)		
5-Fluorouracil, Oxaliplatin, Docetxel	1 (0.7)	0 (0.0)		
5-FU	1 (0.7)	2 (4.8)		
Adriamycin and cyclophosphamide followed by Paclitaxel	1 (0.7)	0 (0.0)		
Capecitabine	2 (1.4)	2 (4.8)		
Carboplatin and Etoposide	4 (2.8)	0 (0.0)		
Carboplatin and Paclitaxel	7 (4.8)	2 (4.8)		
Carboplatin and Pemetrexed	2 (1.4)	1 (2.4)		
Cisplatin	2 (1.4)	0 (0.0)		
Cisplatin and Etoposide	0 (0.0)	1 (2.4)		
Cisplatin and Gemcitabine	0 (0.0)	1 (2.4)		
Cisplatin and Paclitaxel	0 (0.0)	1 (2.4)		
Cisplatin and Pemetrexed	4 (2.8)	0 (0.0)		
Cisplatin and Vinorelbine	7 (4.8)	0 (0.0)		
Cisplatin, Doxorubicin and Cyclophophamide	1 (0.7)	1 (2.4)		
Cisplatin, Pemetrexed	1 (0.7)	0 (0.0)		
Dacarbazin, Cisplatin and Vindesin	1 (0.7)	0 (0.0)		
Dacarbazine	3 (2.1)	0 (0.0)		
Docetaxel	2 (1.4)	0 (0.0)		
Docetaxel, Doxorubicine and Cyclophosphamide	1 (0.7)	0 (0.0)		
Epirubicine and Cyclophosphamide followed by Paclitaxel	34 (23.4)	4 (9.5)		
FLOT	1 (0.7)	6 (14.3)		
Fluoruracil	1 (0.7)	0 (0.0)		
FOLFIRI	2 (1.4)	0 (0.0)		
FOLFOX	1 (0.7)	3 (7.1)		
Gemcitabine and Cisplatin	3 (2.1)	0 (0.0)		
Gemcitabine and Paclitaxel	1 (0.7)	0 (0.0)		
Gemicitabine and Carboplatin	1 (0.7)	0 (0.0)		
Paclitaxel	1 (0.7)	4 (9.5)		
Paclitaxel and Carboplatin	1 (0.7)	0 (0.0)		
Paclitaxel and Ifosfamid	2 (1.4)	0 (0.0)		
Temozolomide and Capecitabine	1.0 (0.7)	0 (0.0)		
Unknown	0	1		
Targeted Therapy Before LMD Diagnosis, n (%)			0.056	0.081
No targeted therapy	93 (64.1)	24 (55.8)		
Abemaciclib	0 (0.0)	1 (2.3)		
Afatinib	1 (0.7)	0 (0.0)		
Axcitinib	0 (0.0)	2.0 (4.6)		
Axitinib	0 (0.0)	1 (2.3)		
Bevacizumab	8 (5.5)	5 (11.6)		
Bevacizumab, Palbociclib	0 (0.0)	1 (2.3)		
Binimetinib	1 (0.7)	0 (0.0)		
Binimetinib and Encorafinib	1 (0.7)	0 (0.0)		
Brigatinib	1 (0.7)	0 (0.0)		
Cabozatinib and Sunitinib	1 (0.7)	0 (0.0)		
Crizotinib	1 (0.7)	0 (0.0)		
Enzalutamide	1 (0.7)	0 (0.0)		
Everolimus	1 (0.7)	0 (0.0)		
Lapatinib	1 (0.7)	1 (2.3)		
Nintedanib	1 (0.7)	1 (2.3)		
Osimertinib	1 (0.7)	1 (2.3)		
Palbociclib	4 (2.8)	3 (7.0)		
Pazpanib	0 (0.0)	1 (2.3)		
Ramucirumab	1 (0.7)	2 (4.7)		
Sacituzumab-Govitecan	1 (0.7)	0 (0.0)		
Sunitinib	1 (0.7)	0 (0.0)		
Sunitinib and Pazopanib	1 (0.7)	0 (0.0)		
Trametinib	1 (0.7)	0 (0.0)		
Trametinib and Dabrafenib	7 (4.8)	0 (0.0)		
Trastuzumab	8 (5.5)	0 (0.0)		
Trastuzumab and Pertuzumab	8 (5.5)	1 (2.3)		
Vemurafenib	1 (0.7)	0 (0.0)		
Checkpoint Inhibitors Before LMD Diagnosis, n (%)			0.52	0.57
No immunotherapy	110 (75.9)	36 (83.7)		
Atezolizumab	0 (0.0)	1 (2.3)		
Avelumab	1 (0.7)	0 (0.0)		
Durvalumab	1 (0.7)	0 (0.0)		
Interferon	1 (0.7)	0 (0.0)		
Nivolumab	8 (5.5)	1 (2.3)		
Nivolumab and Ipilimumab	12 (8.3)	1 (2.3)		
Pembrolizumab	12 (8.3)	4 (9.3)		
Local Treatment Before LMD Diagnosis, n (%)			< 0.001	< 0.001
None	49 (33.8)	35 (81.4)		
Brain metastasis resection	10 (6.9)	0 (0.0)		
Brain metastasis resection and radiation therapy	54 (37.2)	0 (0.0)		
Radiation therapy	29 (20.0)	0 (0.0)		
Radiation therapy (SNUC)	0 (0.0)	1 (2.3)		
Radiation therapy (spine metastases)	0 (0.0)	4 (9.3)		
Surgery (spinal intervention)	0 (0.0)	1 (2.3)		
Surgery (spinal intervention) and radiation therapy (spine metastases)	3 (2.1)	2 (4.7)		
Radiation Therapy Before LMD Diagnosis, n (%)			< 0.001	< 0.001
No radiation therapy	61 (42.1)	36 (83.7)		
Conventional radiation therapy	8 (5.5)	5 (11.6)		
Conventional radiation therapy (later WBRT)	1 (0.7)	0 (0.0)		
CSI	0 (0.0)	2 (4.7)		
SRS	56 (38.6)	0 (0.0)		
SRS (later WBRT)	1 (0.7)	0 (0.0)		
WBRT	14 (9.7)	0 (0.0)		
WBRT (later conventional radiation therapy)	1 (0.7)	0 (0.0)		
WBRT (later SRS)	3 (2.1)	0 (0.0)		

Treatment-related patient characteristics before LMD diagnosis grouped according to the lack of brain metastasis in previous patient history and lack of brain metastasis at the time of LMD diagnosis vs. brain metastasis in the previous patient history as well as the presence of brain metastasis at the time of LMD diagnosis (CNS status) including local surgical procedures such as micorsurgical brain metastasis resection, ventriculoperitoneal (VP) shunt placement, reservoir placement or a combination of these (in context of made LMD diagnosis); serial treatment, i.e. VP shunt placement following resection is indicated in the table; systemic therapies were classified into chemotherapy, targeted therapy and immunotherapy; Craniospinal irradiation (CSI), stereotactic radiosurgery (SRS), whole brain radiation (WBRT). If sequential radiotherapy was performed, this was indicated in the table (e.g., “SRS (later WBRT)”)

Sequential VP shunt placement was performed due to the new onset of hydrocephalus in the course of the disease

^*1*^ Pearson’s Chi-squared test; Fisher’s exact test

^*2*^ Benjamini & Hochberg correction for multiple testing

**Table 3:** Treatment-related characteristics after LMD diagnosis

Variable	CNS parenchymal disease, N = 145	No CNS parenchymal disease, N = 43	p-value^*1*^	q-value^*2*^
History intracranial metastases, n (%)	119 (82)	0 (0)	< 0.001	< 0.001
Intrathecal Therapy After Diagnosis of LMD, n (%)			0.24	0.42
No i.t. therapy	118 (81.4)	30 (69.8)		
i.t. therapy via repetitive LP	16 (11.0)	7 (16.3)		
i.t. therapy via reservoir	11 (7.6)	6 (14.0)		
Chemotherapy After Diagnosis of LMD, n (%)			0.024	0.084
No chemotherapy	127 (87.6)	32 (74.4)		
5-FU	1 (0.7)	1 (2.3)		
Adriamycin, Cyclophosphamide and Vincristine	0 (0.0)	1 (2.3)		
Capecitabine	6 (4.1)	1 (2.3)		
Carboplatin	1 (0.7)	0 (0.0)		
Carboplatin and Etoposide	1 (0.7)	0 (0.0)		
Carboplatin and nab-Paclitaxel	2 (1.4)	0 (0.0)		
Carboplatin, Paclitaxel	1 (0.7)	0 (0.0)		
Cisplatin	1 (0.7)	1 (2.3)		
Cisplatin and Etoposide	1 (0.7)	0 (0.0)		
Cisplatin, Pemetrexed and Carboplatin	1 (0.7)	0 (0.0)		
Docetaxel	0 (0.0)	1 (2.3)		
Doxorubicin	1 (0.7)	0 (0.0)		
Doxorubicin and MTX	0 (0.0)	1 (2.3)		
FLOT	0 (0.0)	1 (2.3)		
FOLFIRI	0 (0.0)	1 (2.3)		
Gemcitabine	1 (0.7)	0 (0.0)		
Paclitaxel	1 (0.7)	1 (2.3)		
Topotecan	0 (0.0)	1 (2.3)		
Trifluridin and Tipiracil	0 (0.0)	1 (2.3)		
Targeted Therapy After Diagnosis of LMD, n (%)			0.60	0.70
No targeted therapy	118 (81.4)	37 (86.0)		
Axitinib	1 (0.7)	0 (0.0)		
Bevacizumab	1 (0.7)	1 (2.3)		
Binimetinib	1 (0.7)	0 (0.0)		
Crizotinib	2 (1.4)	0 (0.0)		
Eribulin	1 (0.7)	0 (0.0)		
Erlotinib	0.0 (0.0)	1 (2.3)		
Lapatinib	3 (2.1)	0 (0.0)		
Nintedanib	0 (0.0)	1 (2.3)		
Olaparib	1 (0.7)	0 (0.0)		
Osimertinib	2 (1.4)	0 (0.0)		
Palbociclib	1 (0.7)	0 (0.0)		
Pazopanib	0 (0.0)	1 (2.3)		
Ramucirumab	1 (0.7)	0 (0.0)		
Sacituzumab-Govitecan	2 (1.4)	1 (2.3)		
Sorafenib	1 (0.7)	0 (0.0)		
Trametinib and Dabrafenib	3 (2.1)	0 (0.0)		
Trastuzumab	1 (0.7)	0 (0.0)		
Trastuzumab-Deruxtecan	1 (0.7)	0 (0.0)		
Trastuzumab-Emtansin	4 (2.8)	0 (0.0)		
Trastuzumab-Emtansin and Tucatinib	1 (0.7)	1 (2.3)		
Checkpoint Inhibitors After Diagnosis of LMD, n (%)			0.43	0.61
No immunotherapy	134 (92.4)	38 (88.4)		
Atezolizumab	1 (0.7)	0 (0.0)		
Nivolumab	2 (1.4)	0 (0.0)		
Nivolumab, Ipilimumab	3 (2.1)	3 (7.0)		
Pembrolizumab	5 (3.4)	2 (4.7)		
Local Therapies After Diagnosis of LMD, n (%)			0.72	0.72
Brain metastasis resection	6 (4.1)	0.0 (0.0)		
Brain metastasis resection and later reservoir placement	2 (1.4)	0 (0.0)		
Brain metastasis resection and later VP shunt placement	1 (0.7)	0 (0.0)		
Brain metastasis resection and reservoir placement	2 (1.4)	0 (0.0)		
Brain metastasis resection and VP shunt placement	2 (1.4)	0 (0.0)		
Brain metastasis resection and VP shunt placement followed by WBRT	1 (0.7)	0 (0.0)		
Brain metastasis resection followed by conventional radiotherapy	1 (0.7)	0 (0.0)		
Brain metastasis resection followed by CSI	2 (1.4)	0 (0.0)		
Brain metastasis resection followed by WBRT	7 (4.8)	0 (0.0)		
Brain metastasis resection, reservoir placement followed by SRS	1 (0.7)	0 (0.0)		
Conventional radiation therapy	3 (2.1)	2 (4.7)		
CSI	4 (2.8)	0 (0.0)		
None	52 (35.9)	20.0 (46.5)		
Reservoir placement	7 (4.8)	6 (14.0)		
Reservoir placement and followed by WBRT	1 (0.7)	1 (2.3)		
Reservoir placement and VP shunt placement	1 (0.7)	0 (0.0)		
Reservoir placement and WBRT	0 (0.0)	1 (2.3)		
Reservoir placement followed by SRS	1 (0.7)	0 (0.0)		
Reservoir placement followed by WBRT	2 (1.4)	0 (0.0)		
SRS	7 (4.8)	0 (0.0)		
Surgery (spinal intervention)	1 (0.7)	1 (2.3)		
Surgery and radiation therapy	1 (0.7)	0 (0.0)		
VP shunt placement followed by WBRT	1 (0.7)	0 (0.0)		
VP shunt placement	5 (3.4)	2 (4.7)		
VP shunt placement and later reservoir placement	1 (0.7)	0 (0.0)		
VP shunt placement and later reservoir placement followed by CSI	1 (0.7)	0 (0.0)		
VP shunt placement followed by WBRT	1 (0.7)	1 (2.3)		
WBRT	31 (21.4)	9 (20.9)		
Radiation Therapy After Diagnosis of LMD, n (%)			0.22	0.42
No radiation therapy	80 (55.2)	29 (67.4)		
Conventional radiation therapy	5 (3.4)	2 (4.7)		
CSI	7 (4.8)	0 (0.0)		
SRS	9 (6.2)	0 (0.0)		
WBRT	44 (30.3)	12 (27.9)		

Treatment-Related Patient Characteristics After LMD Diagnosis grouped according to the history of previous brain metastasis before LMD diagnosis, including local intrathecal (i.t.) methotrexate (MTX) via repetitive lumbar punctures or a Rickham reservoir, systemic therapies or local radiation therapy

*CSI* craniospinal irradiation, *SRS* stereotactic radiosurgery, *WBRT* whole brain radiation

^*1*^ Pearson’s Chi-squared test; Fisher’s exact test

^*2*^ Benjamini & Hochberg correction for multiple testing

## Discussion

LMD represents a critical and late-stage complication of metastatic malignancies characterized by the dissemination of cancer cells into the leptomeninges and CSF. Despite the growing recognition of its clinical importance, comprehensive analyses focusing on the prognostic impact of intracranial parenchymal BrM are limited. Instead, most studies have focused on LMD-specific radiological characteristics or treatments administered upon LMD diagnosis [9, 12, 15, 16]. This study provides a detailed retrospective analysis of a relatively large single-center cohort of different tumor entities with a comprehensive description of patient characteristics and prognostic factors in patients newly diagnosed with LMD. One key finding in our analysis revealed that a history of intracranial parenchymal metastases seems to impact OS in LMD patients, emphasizing the importance of the CNS disease status in the prognosis of LMD patients, aligning with the increasing body of evidence that CNS involvement dictates a more aggressive clinical course and a poorer prognosis. In this regard, our data also highlight that HCP, indicative of significant CSF flow disruption and increased intracranial pressure, at the time of LMD diagnosis is associated with a more severe disease state and consequently poorer outcomes. This also goes hand in hand with our finding showing a trend of higher risk of death in patients who got tumor cells detected upon first lumbar puncture. In our study, we provide more insights into the context of BrM burden and extracranial disease burden, differentiating between the history of BrM or EcM before LMD diagnosis and the presence of BrM and EcM at the time of LMD diagnosis as well as the activity of BrM and EcM according to RANO or RECIST criteria. In terms of the activity of extracranial disease, which was in part discussed in other retrospective studies, we differentiated between EcM and the primary tumor mass [8, 9]. In contrast, the presence or activity of extracranial metastases as well as the activity of the primary tumor, often considered as drivers of disease progression, did not show a significant association with survival outcomes, which could suggest that once LMD is diagnosed, it becomes the predominant factor affecting patient mortality, regardless of extracranial disease status. Interestingly, patients with no previous history of CNS parenchymal disease showed higher frequency of extracranial metastases at time of LMD diagnosis. 

Our findings indicate that systemic therapies administered after LMD diagnosis, particularly TT and radiation therapy, are associated with improved survival outcomes [16]. The use of TT post-LMD diagnosis significantly prolonged OS, reflecting the efficacy of these treatments in controlling disease progression within the CNS and beyond. This supports the current clinical guidelines advocating for the use of systemic treatments, including chemotherapy and TT, in conjunction with local interventions like radiation therapy. Interestingly, no significant difference in survival was observed between patients receiving intrathecal methotrexate (i.t. MTX) and those who did not receive this treatment, yet i.t. therapy itself provided a survival advantage. This may be due to the limited sample size of patients undergoing i.t. therapy in our cohort. It is also noteworthy that the route of i.t. MTX administration, whether via a reservoir or repetitive lumbar punctures, did not influence survival outcomes. These findings suggest the need for further research to establish the efficacy of i.t. therapies and to optimize their administration methods in LMD patients.

Several limitations of our study warrant discussion. The retrospective design and single-center setting may limit the generalizability of the findings. The heterogeneity of the included tumor entities and the variability in treatment regimens before and after LMD diagnosis could also influence the identified prognostic factors. Additionally, due to the short survival of many patients and the lack of comprehensive follow-up data, we could not assess the impact of subsequent treatment responses or changes in disease status over time. Furthermore, we did not include data on follow-up clinical visits and follow-up imaging, including MRI or CT staging to evaluate MRI or CT response (CNS-specific progression-free survival and extra-CNS progression-free survival) or serial CSF sampling to evaluate CSF response. This is due to missing data for most patients, given the short survival and the lack of outpatient follow-up visits, as most patients were referred to outpatient palliative care doctors outside of our institution. This holds also true for best supportive and palliative care intervention as quality-of-life assessment which were not part of this study. Moreover, we did not make use of the graded prognostic assessment (GPA) or Leptomeningeal Assessment in Neuro-Oncology (LANO) neurological assessment in our study. However, we assessed other important clinical factors such as KPS, age, and the presence of other non-oncological diseases as prognostic factors [21]. Moreover, not all patients underwent complete work-up according to EANO-ESMO guidelines, including CSF cytology analysis and complete imaging of the neuro-axis. We included data on CSF cytology, which cannot be considered as a test for sensitivity or accuracy of CSF cytology given that not all patients received CSF analysis, and our patients were diagnosed and subsequently treated by different treating physicians (i.e. oncologists, neurosurgeons, nerologists, radiooncologists, dermatologists and gynecologists). Future research should aim to overcome these limitations by involving larger, multi-center cohorts and incorporating prospective data collection to better understand the longitudinal impact of treatments and disease progression in LMD patients. Prospective studies focusing on the prognostic implications of BrM and EcM, as well as the efficacy of specific therapeutic strategies, are crucial for refining treatment guidelines and improving patient outcomes. In conclusion, our study highlights the significant prognostic role of intracranial BrM and the limited impact of extracranial disease burden in patients with newly diagnosed LMD. The findings underscore the importance of aggressive and tailored treatment strategies, particularly the use of targeted therapies and radiation therapy, in improving survival outcomes. Despite these limitations, our findings offer insights and underscore the heterogeneity and complexity of administered local and systemic treatments and the role of intracranial BrM and extracranial disease burden (EcM and primary tumor mass) in patients with newly diagnosed LMD. The median OS of 2.83 months underscores the poor outcome of these patients and the urgency to promote a systematic and interdisciplinary discussion of these cases at diagnosis to improve therapeutic interventions and management as well as follow-up. Median OS time in this cohort was comparable with previously published data [7, 9, 12, 15, 16, 22, 23].

## Conclusion

This work contributes to the growing body of literature advocating for a more nuanced understanding of LMD and the need for entity-specific treatment approaches to enhance patient care. The study emphasizes the necessity of prospective, interdisciplinary, multi-center registries to better capture the complexity and heterogeneity of LMD patient populations and treatment responses, ultimately guiding more effective and personalized yet standardized and timely therapeutic interventions. The significant prognostic impact of the primary tumor type at LMD diagnosis warrants prospective entity-specific trials.

## Supplementary Information

Below is the link to the electronic supplementary material.

Supplementary file1 (PDF 1862 KB)

Supplementary file2 (PDF 1862 KB)

Supplementary file3 (PDF 1862 KB)

Supplementary file4 (DOCX 125 KB)

## Data Availability

No datasets were generated or analysed during the current study.
