# Supplementary material for: Clinical characteristics and outcomes in leptomeningeal disease with or without brain metastasis: insights from an explorative data analysis of the Charité LMD registry
Source: J Neurooncol. 2025 Feb 11;175(3):943–65. doi: 10.1007/s11060-025-04937-x (PMC12511199; doi:10.1007/s11060-025-04937-x)
Supplement: Supplementary file 1 — Supplementary file1 (PDF 1862 KB) [file 11060_2025_4937_MOESM1_ESM.pdf]

S1A

A

Patient screening:

- brain metastasis registry
- patients undergoing VP shunt or reservoir placement
- Tumorboard server (search tags „Lumbalpunktion“, „Mengingeosis“, „LP“, “lepto“

Patients with suspected leptomeningeal metastasis (LMD)

Excluded patients with LMD (n=30):

- Midline glioma (n=2), glioblastoma (n=3), ganglioglioma (n=1), anaplastic Xanthoastrocytoma (n=1)
- Medulloblastmoma (n=3)
- Ependymoma (n=1), pineoblastoma (=1), melanomcytoma (n=1)
- Embryonal rhabdomyosarcoma (n=1)
- DLBCL (n=6), PCNSTCL (n=1), CML (n=2), CLL (n=2), multiple myeloma (n=2)
- Merkel cell carcinoma (n=2)
- Hemangioblastoma (n=1)

Patients with suspected leptomeningeal metastasis (LMD) due to a solid malignancy

Patients with evidence of LMD on MRI +/- CSF studies (n=188)

S1B

B

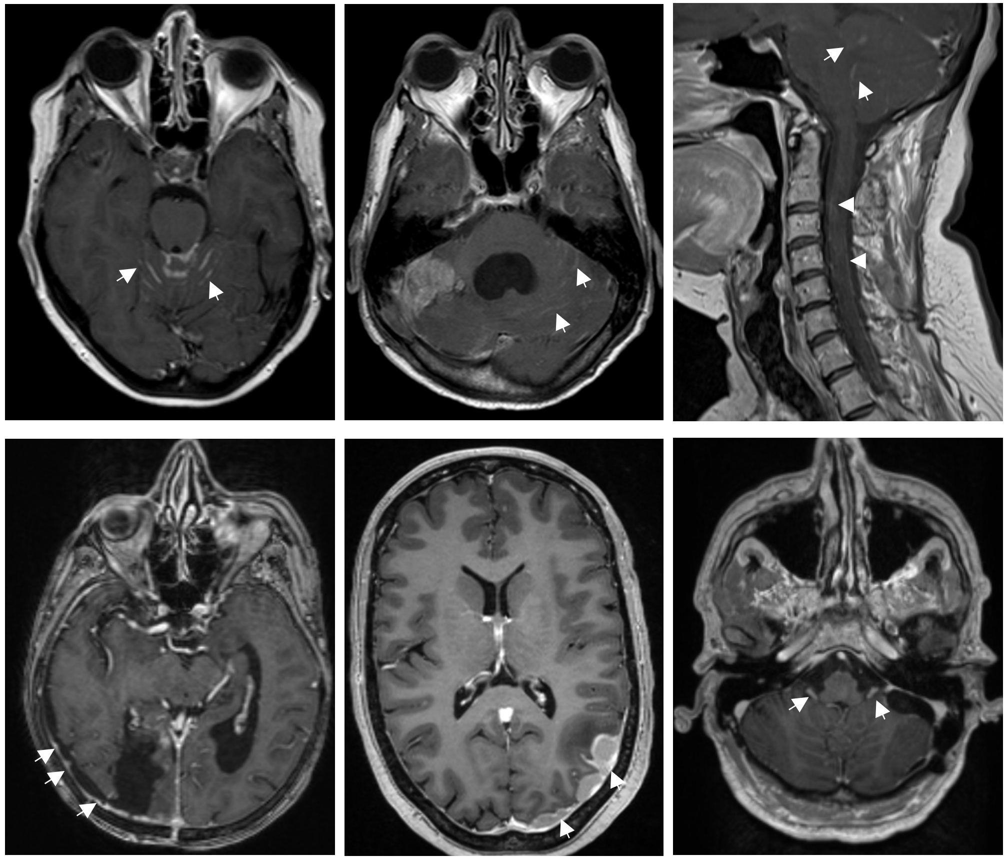

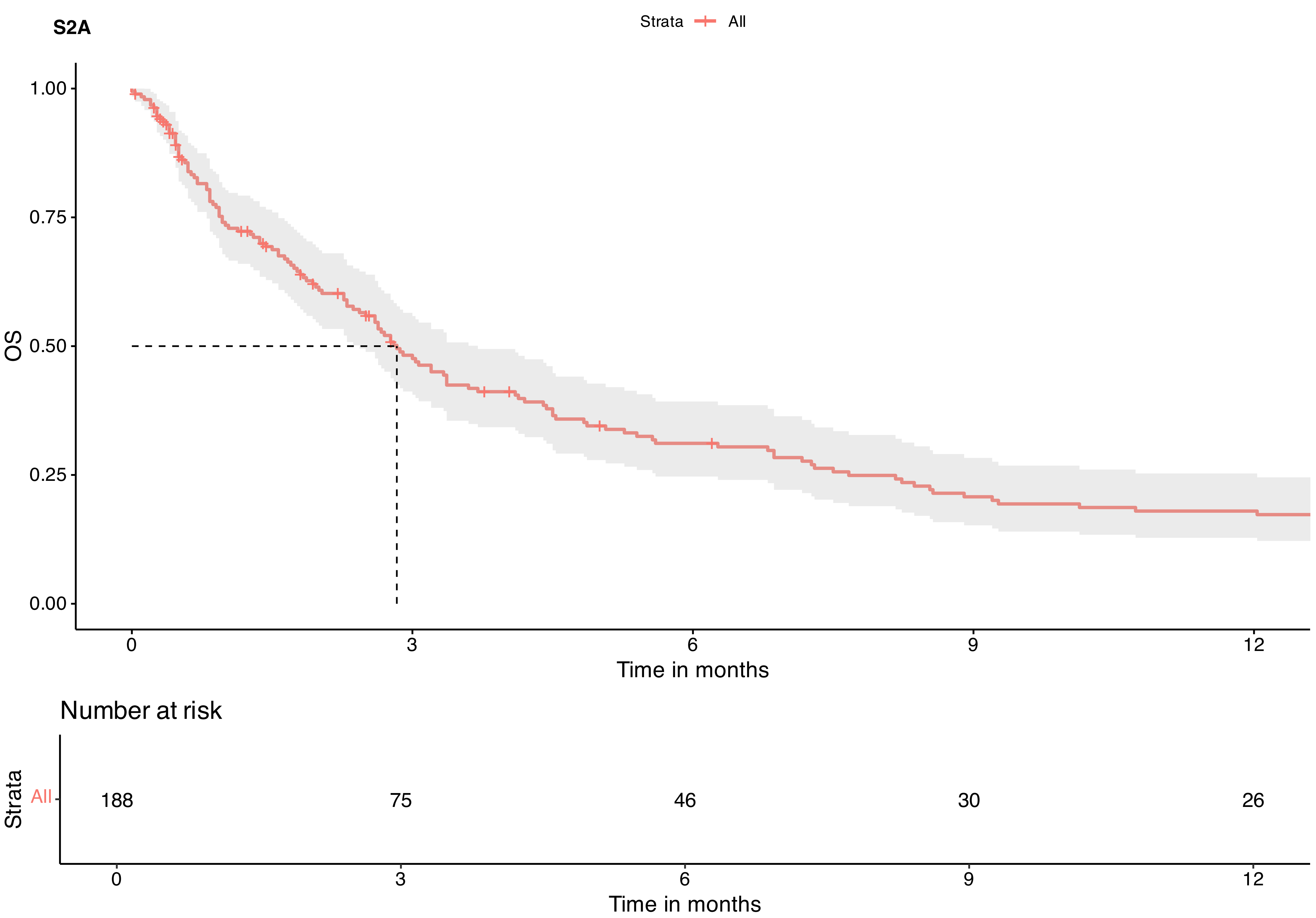
S2A
Strata All
1.00
0.75
0.50
0.25
0.00
OS
0
3
6
9
12
Time in months
Number at risk
Strata
All
188
75
46
30
26
0
3
6
9
12
Time in months

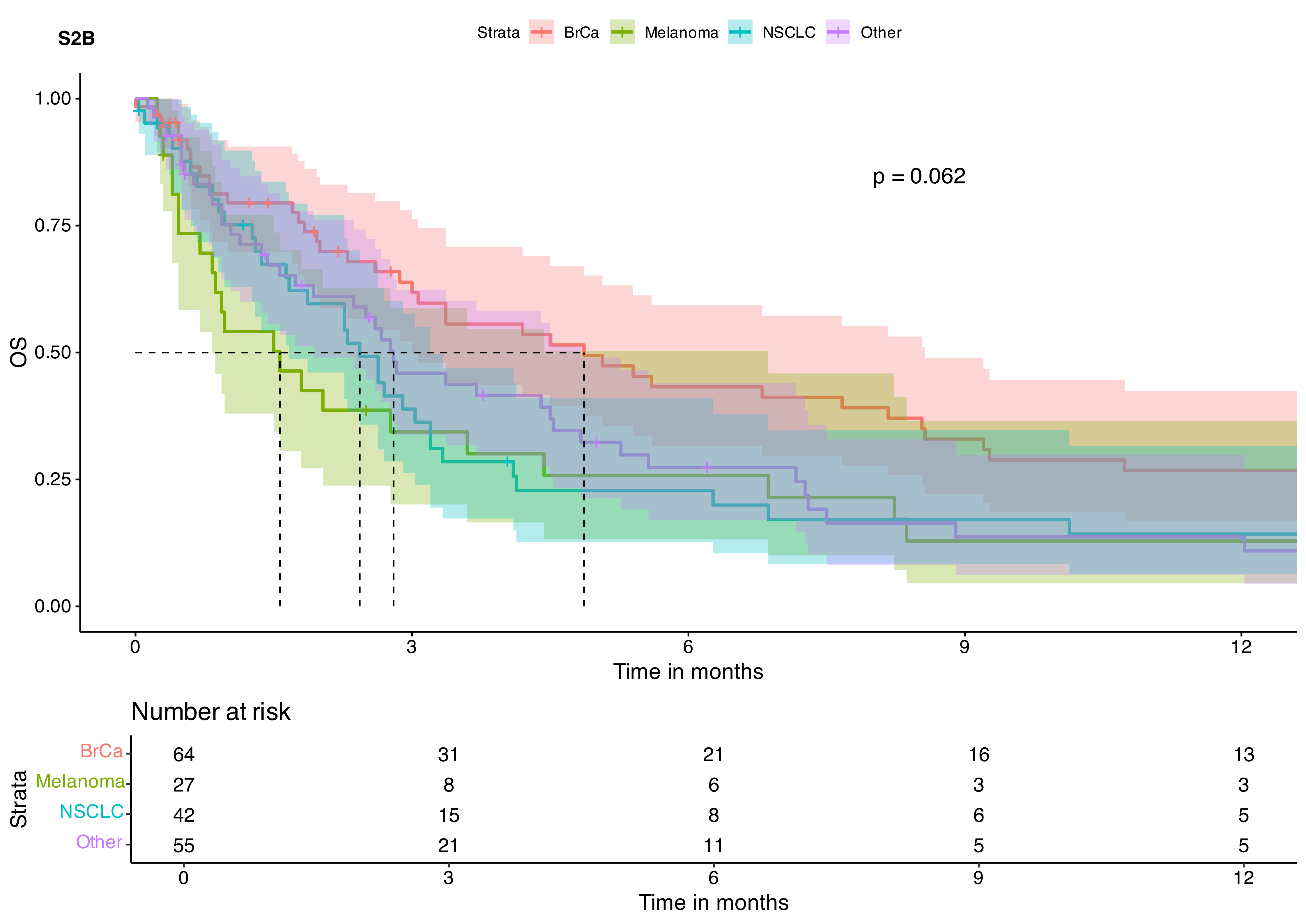
S2B
Strata
BrCa
Melanoma
NSCLC
Other
p = 0.062
OS
1.00
0.75
0.50
0.25
0.00
0
3
6
9
12
Time in months
Number at risk
Strata
BrCa 64 31 21 16 13
Melanoma 27 8 6 3 3
NSCLC 42 15 8 6 5
Other 55 21 11 5 5
0
3
6
9
12
Time in months

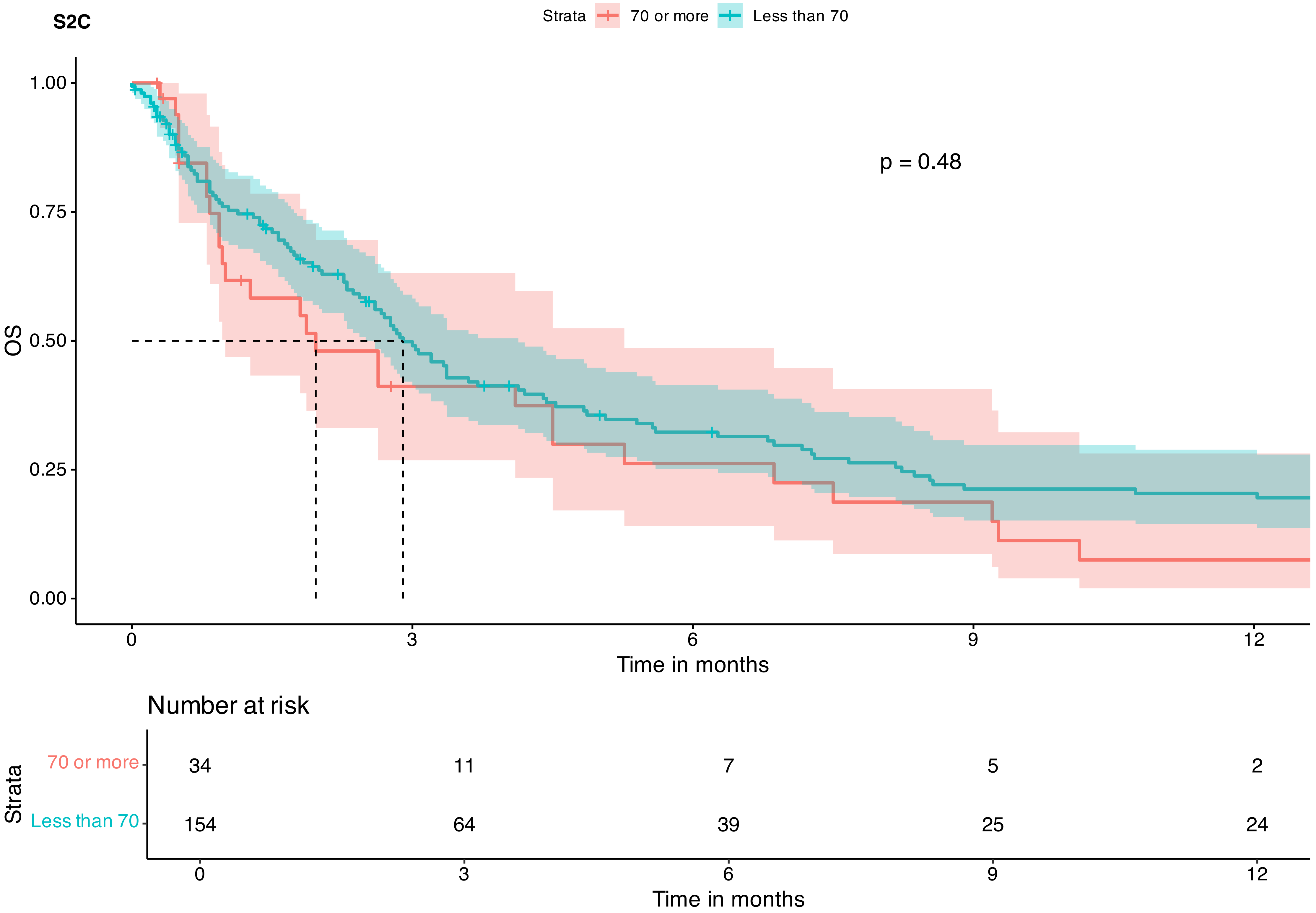
S2C
Strata
70 or more
Less than 70
p = 0.48
OS
1.00
0.75
0.50
0.25
0.00
0
3
6
9
12
Time in months
Number at risk
Strata
70 or more
Less than 70
34
11
7
5
2
154
64
39
25
24
0
3
6
9
12
Time in months

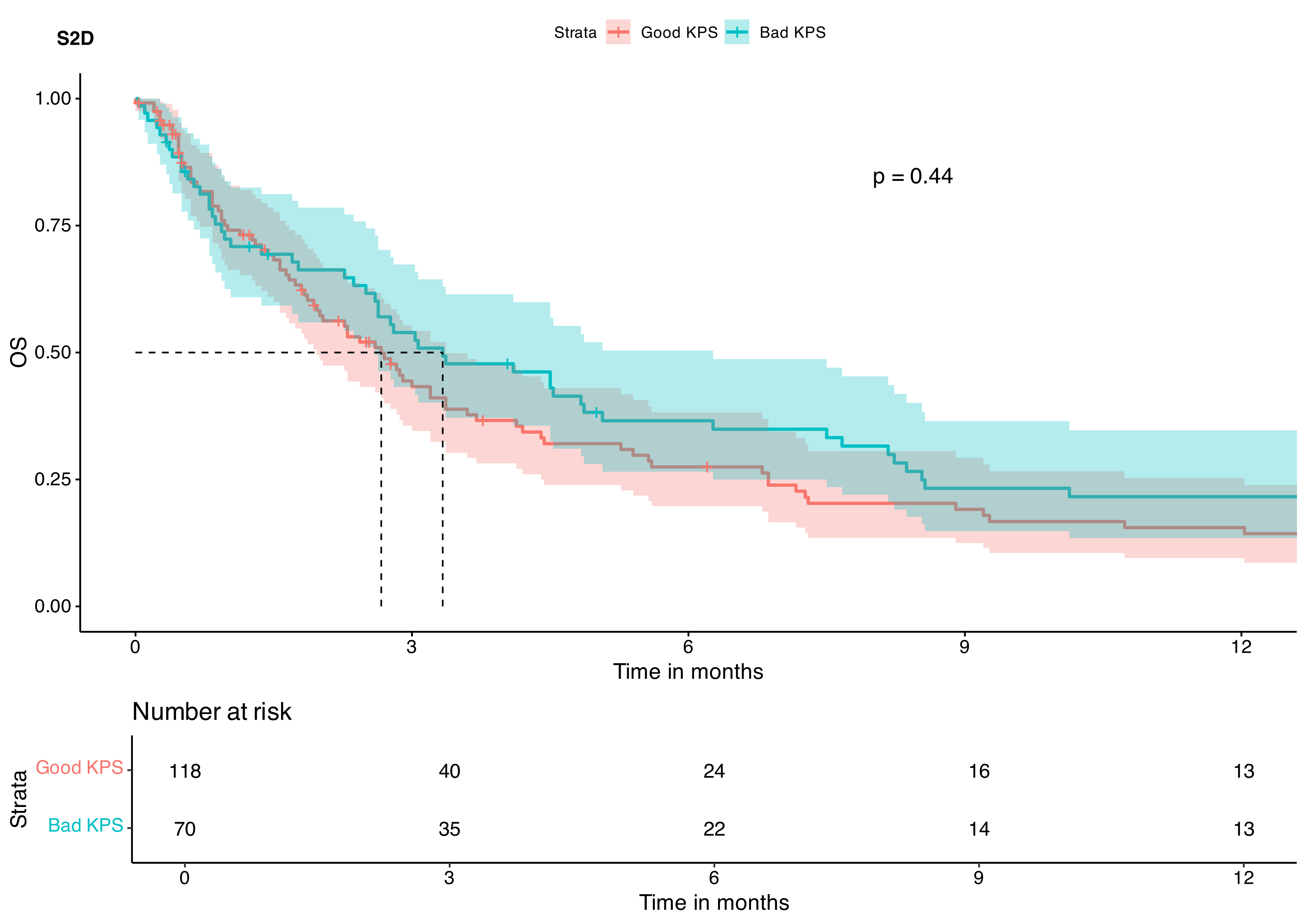
S2D
Strata
Good KPS
Bad KPS
p = 0.44
OS
1.00
0.75
0.50
0.25
0.00
0
3
6
9
12
Time in months
Number at risk
Strata
Good KPS
Bad KPS
118
40
24
16
13
70
35
22
14
13
0
3
6
9
12
Time in months

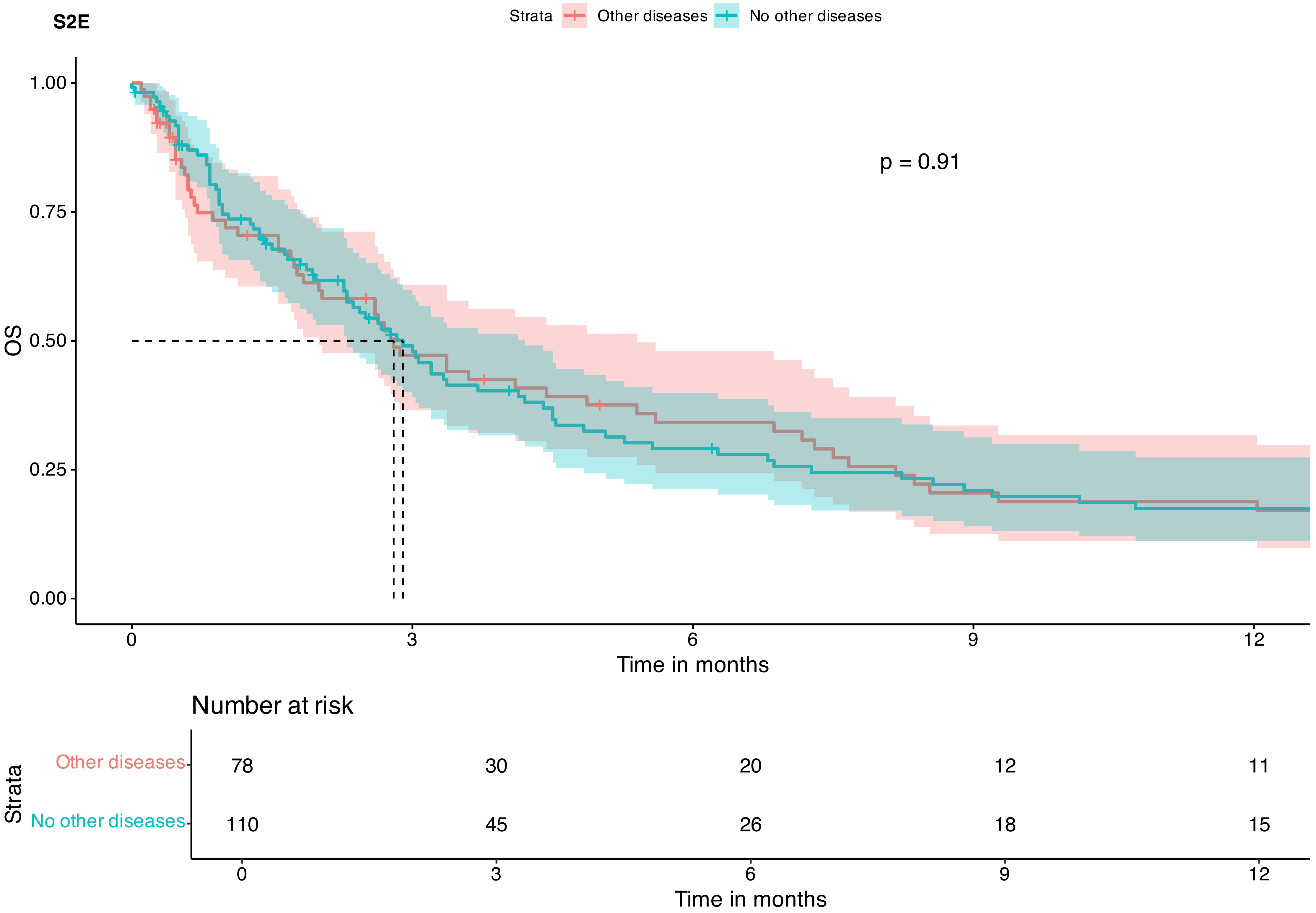

S2E
Strata
Other diseases
No other diseases
p = 0.91
OS
1.00
0.75
0.50
0.25
0.00
0
3
6
9
12
Time in months
Number at risk
Strata
Other diseases
No other diseases
78
30
20
12
11
110
45
26
18
15
0
3
6
9
12
Time in months

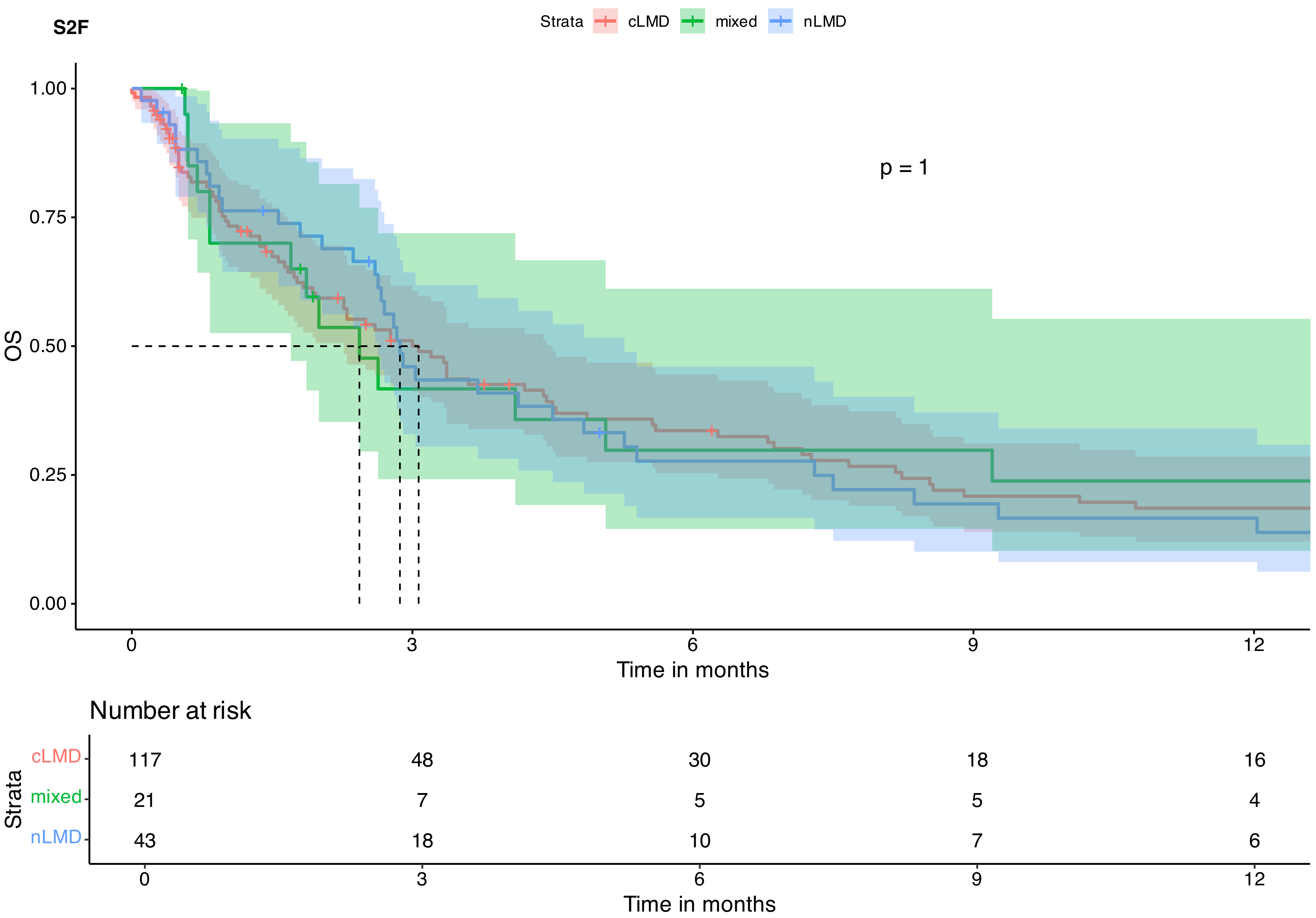
S2F
Strata
cLMD
mixed
nLMD
p = 1
OS
1.00
0.75
0.50
0.25
0.00
0
3
6
9
12
Time in months
Number at risk
Strata
cLMD 117 48 30 18 16
mixed 21 7 5 5 4
nLMD 43 18 10 7 6
0
3
6
9
12
Time in months

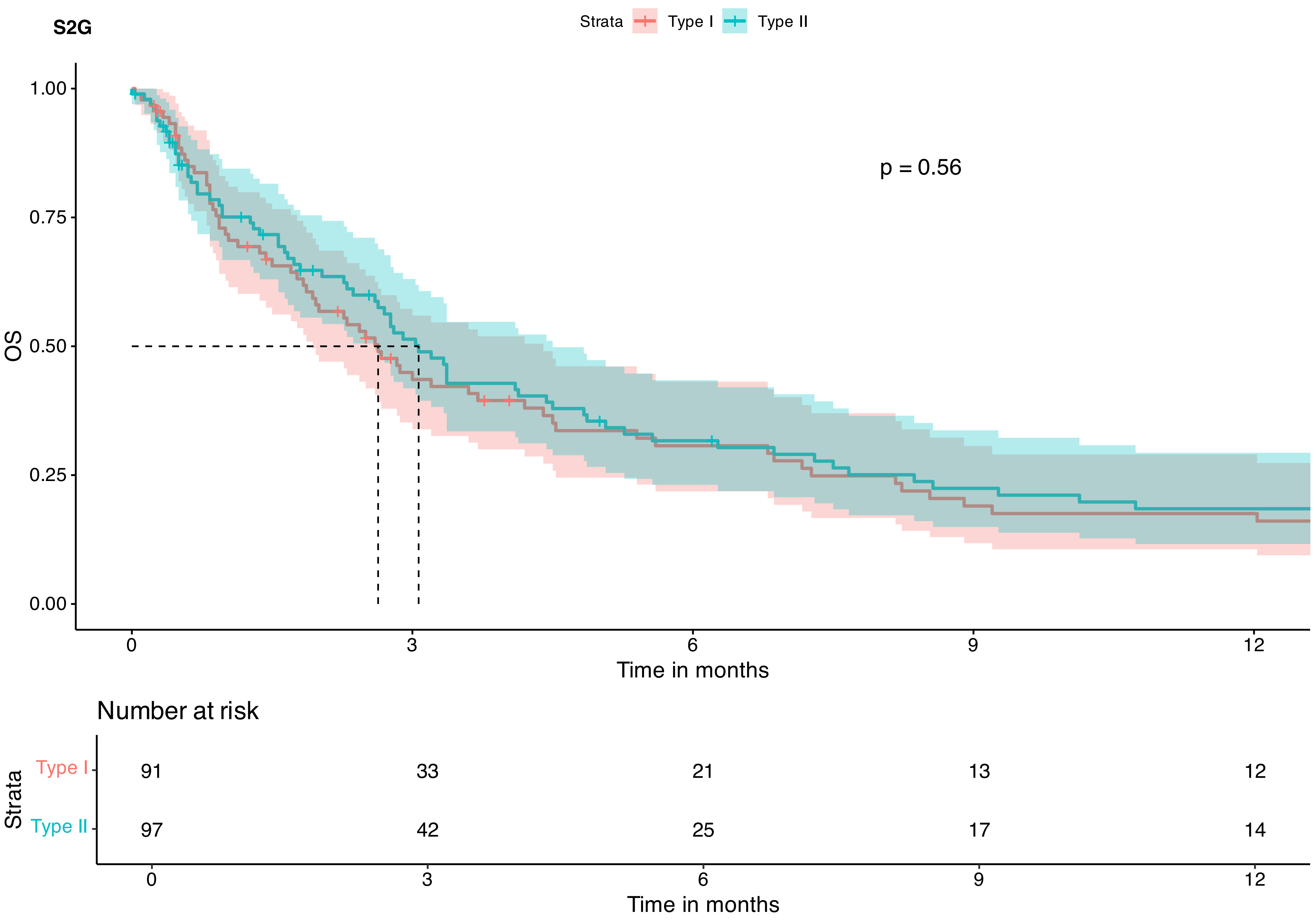

S2G
Strata
Type I
Type II
p = 0.56
OS
1.00
0.75
0.50
0.25
0.00
0
3
6
9
12
Time in months
Number at risk
Strata
Type I 91 33 21 13 12
Type II 97 42 25 17 14
0
3
6
9
12
Time in months

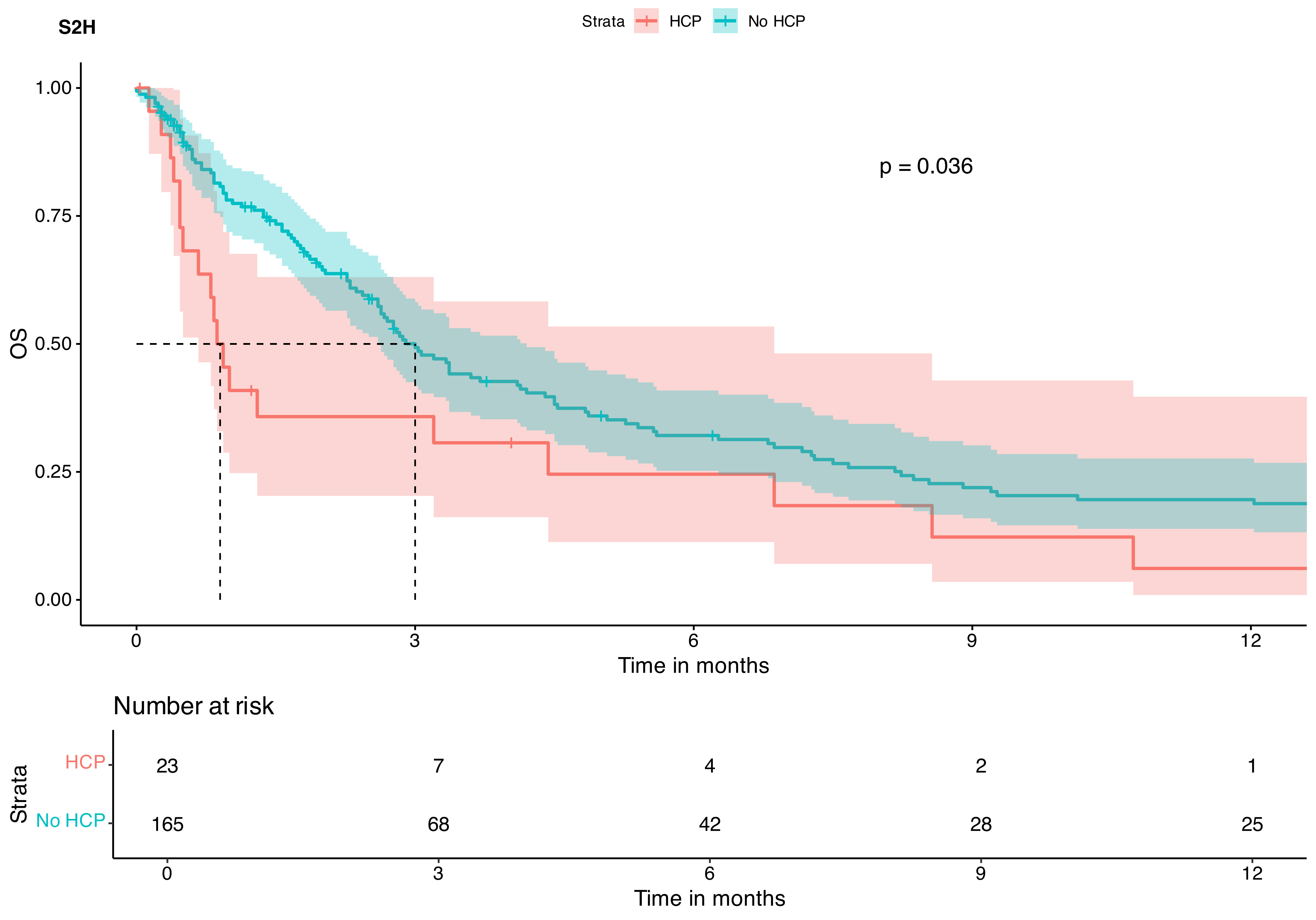
S2H
Strata
HCP
No HCP
p = 0.036
OS
1.00
0.75
0.50
0.25
0.00
0
3
6
9
12
Time in months
Number at risk
Strata
HCP
No HCP
23
7
4
2
1
165
68
42
28
25
0
3
6
9
12
Time in months

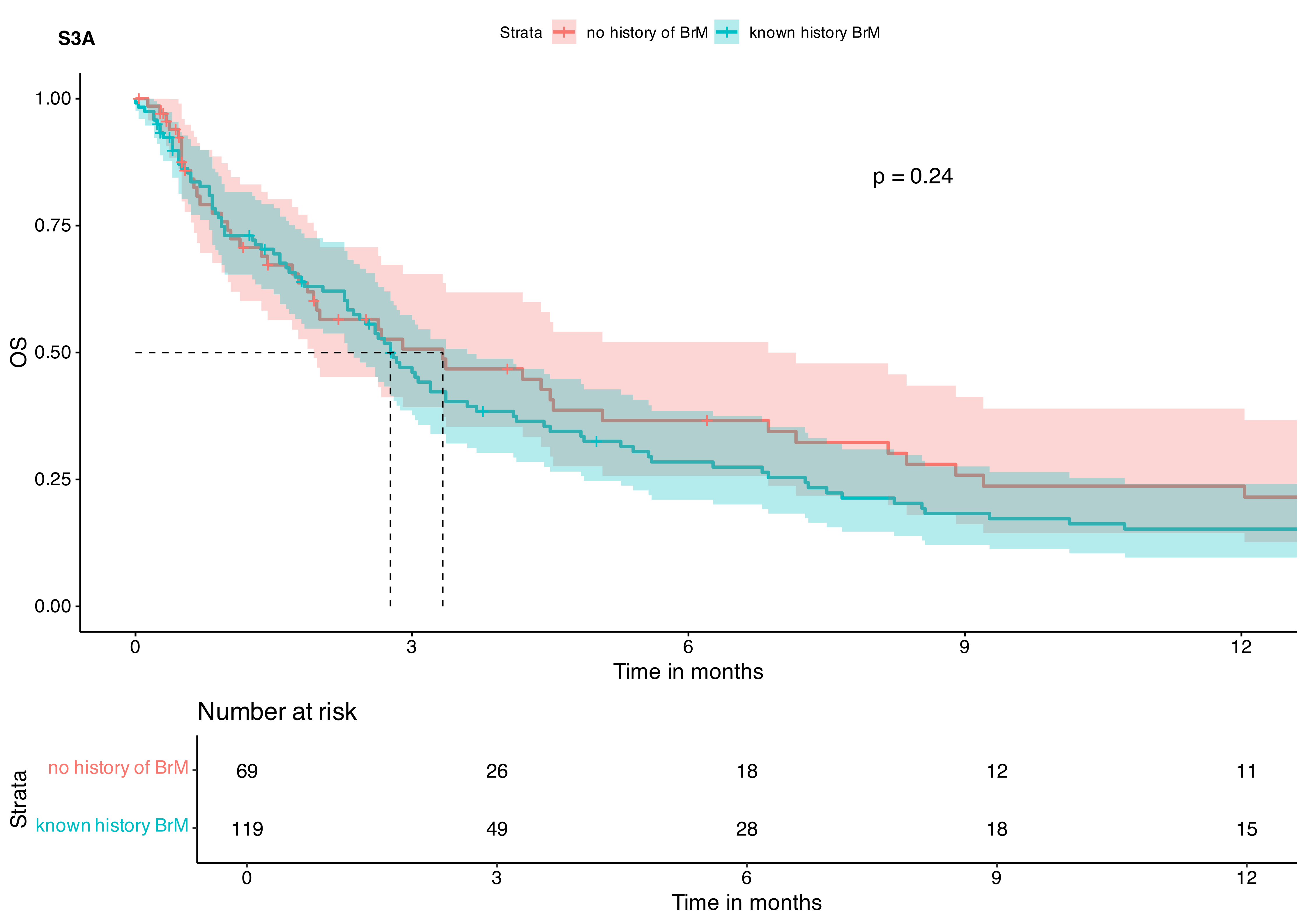

S3A
Strata
no history of BrM
known history BrM
p = 0.24
OS
1.00
0.75
0.50
0.25
0.00
0
3
6
9
12
Time in months
Number at risk
Strata
no history of BrM 69 26 18 12 11
known history BrM 119 49 28 18 15
0
3
6
9
12
Time in months

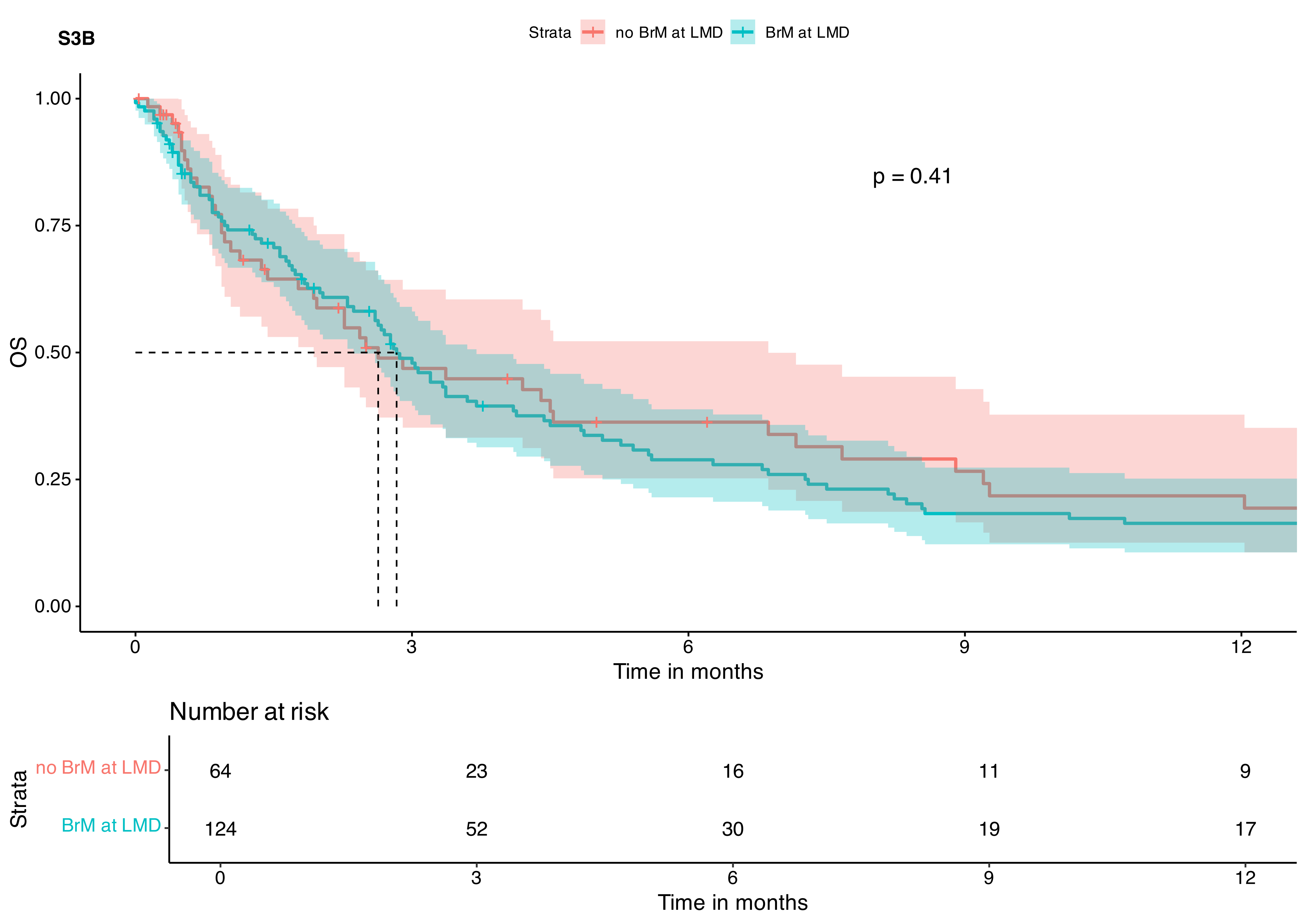

S3B
Strata
no BrM at LMD
BrM at LMD
p = 0.41
OS
1.00
0.75
0.50
0.25
0.00
0
3
6
9
12
Time in months
Number at risk
Strata
no BrM at LMD 64 23 16 11 9
BrM at LMD 124 52 30 19 17
0
3
6
9
12
Time in months

S3C

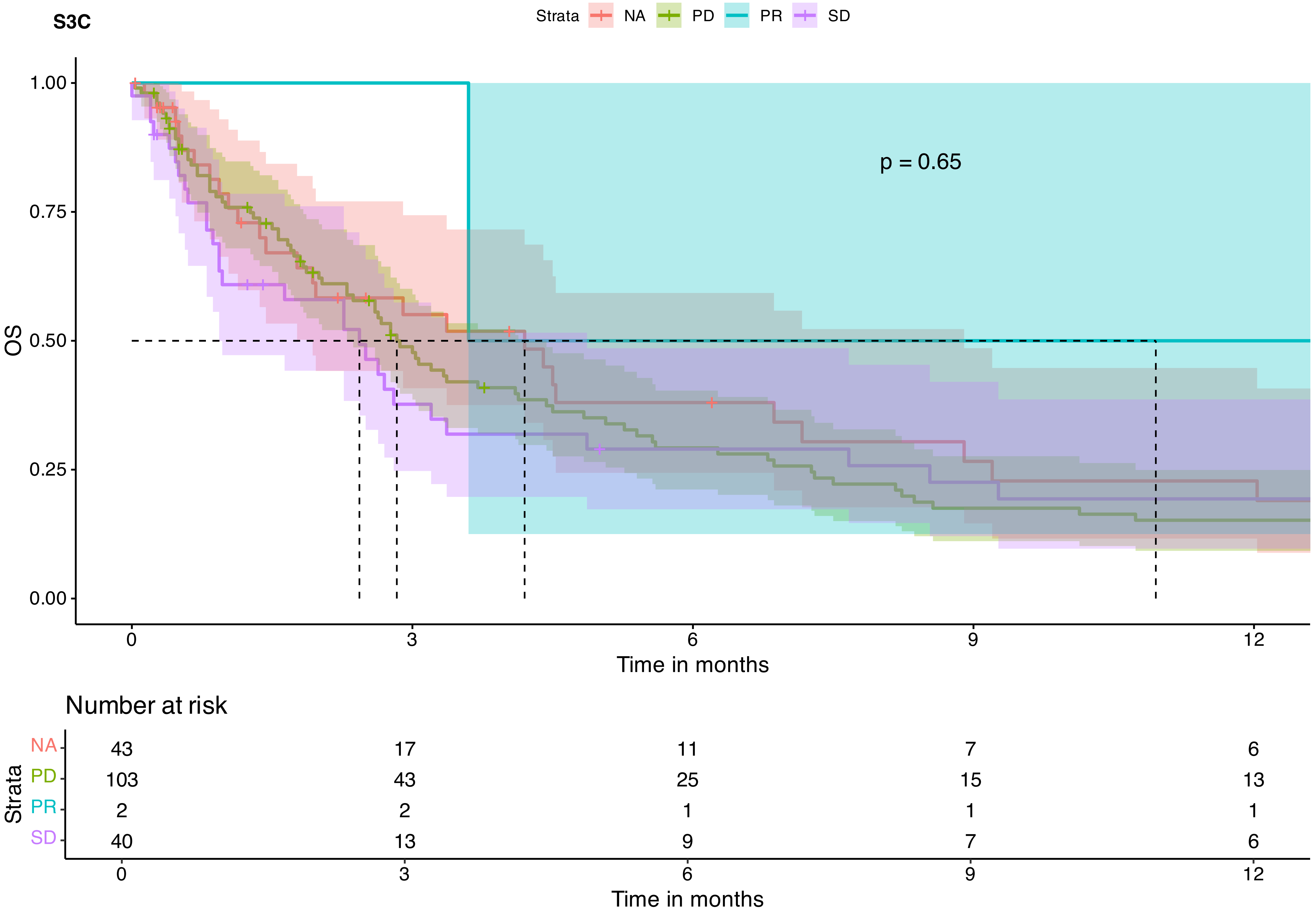

Strata
NA
PD
PR
SD
p = 0.65
OS
1.00
0.75
0.50
0.25
0.00
0
3
6
9
12
Time in months
Number at risk
Strata
NA 43 17 11 7 6
PD 103 43 25 15 13
PR 2 2 1 1 1
SD 40 13 9 7 6
0
3
6
9
12
Time in months

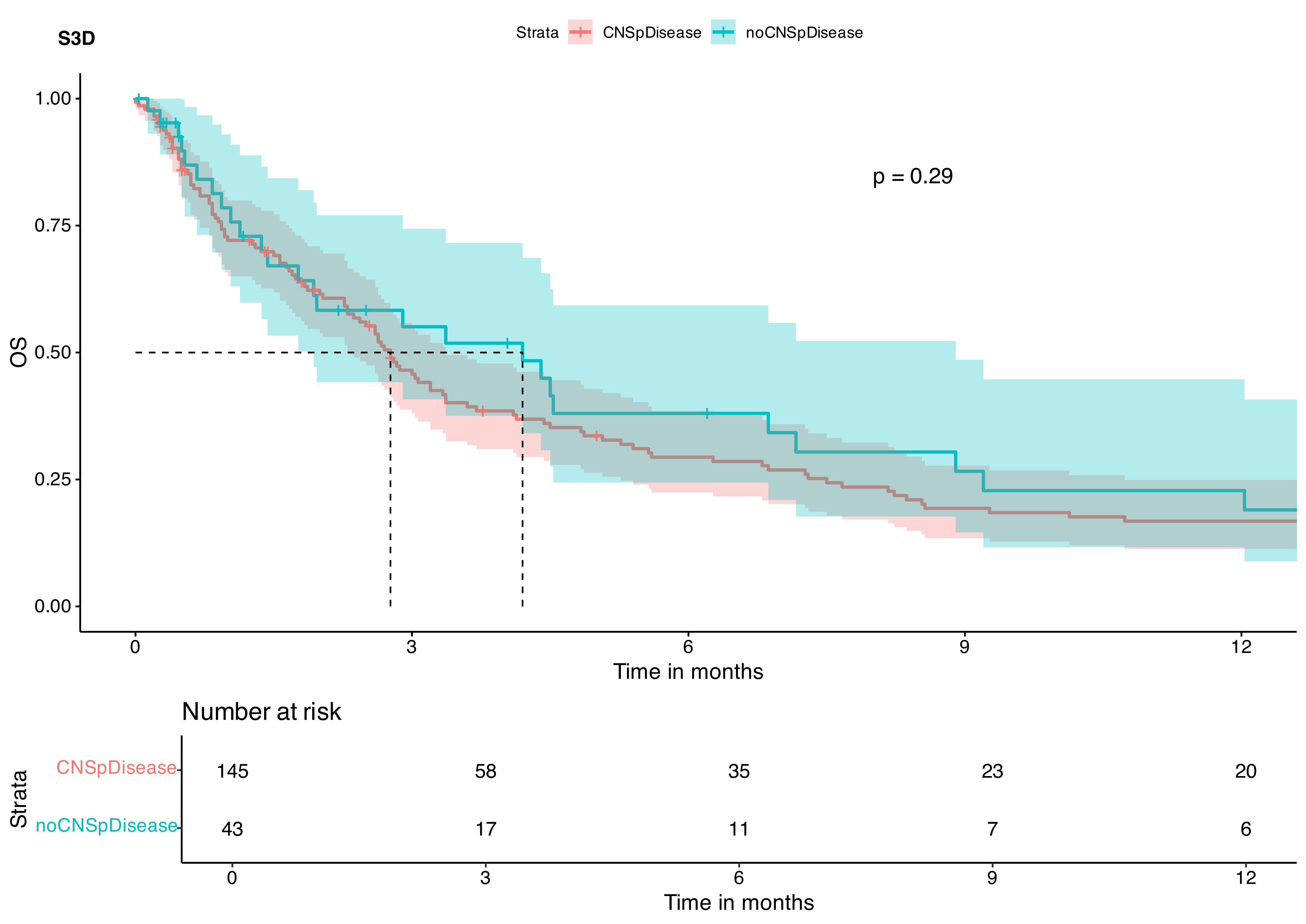
S3D
Strata
CNSpDisease
noCNSpDisease
p = 0.29
OS
1.00
0.75
0.50
0.25
0.00
0
3
6
9
12
Time in months
Number at risk
Strata
CNSpDisease 145 58 35 23 20
noCNSpDisease 43 17 11 7 6
0
3
6
9
12
Time in months

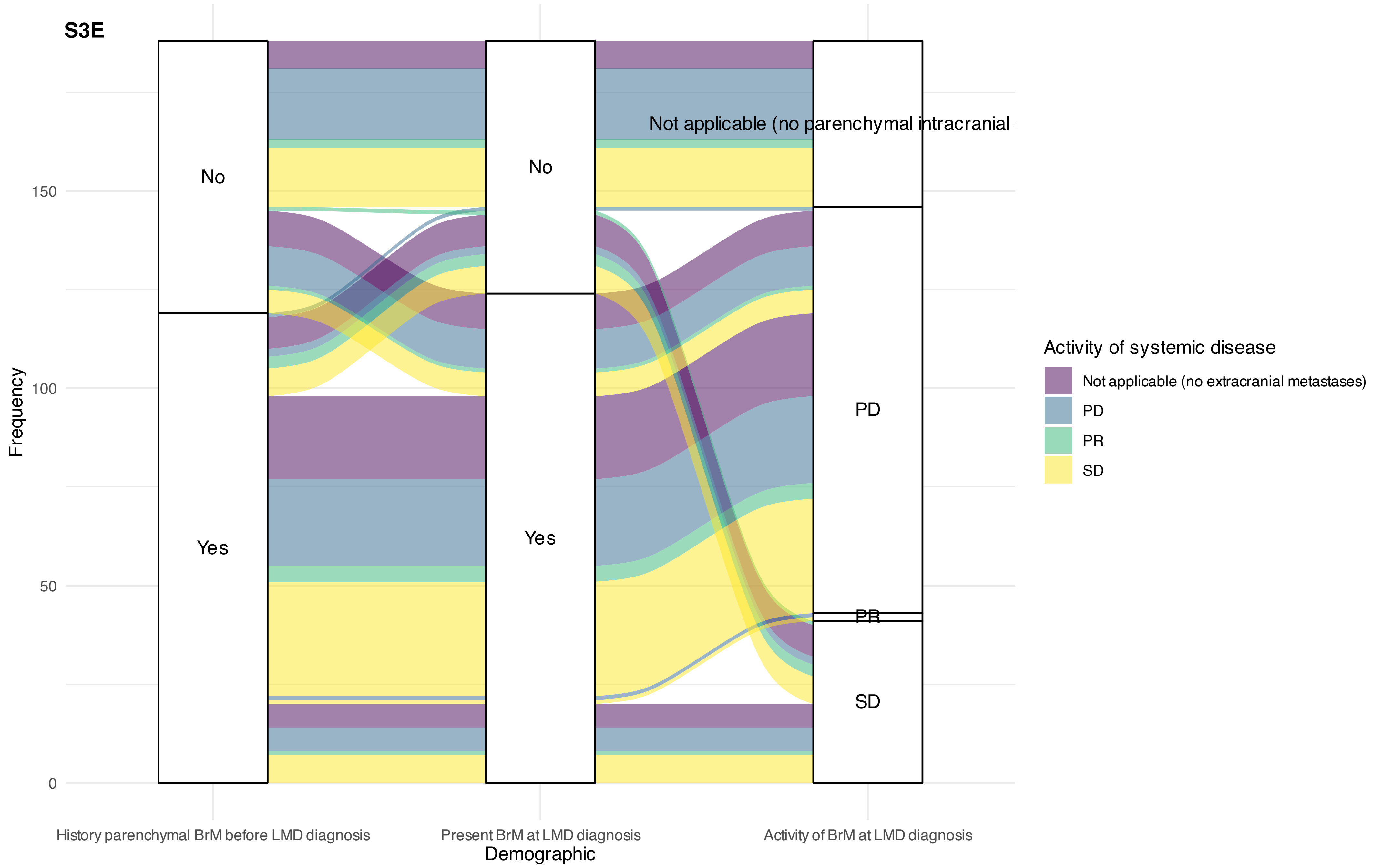
S3E
Frequency
150
100
50
0
No
Yes
No
Yes
Not applicable (no parenchymal intracranial
PD
PR
SD
History parenchymal BrM before LMD diagnosis
Present BrM at LMD diagnosis
Activity of BrM at LMD diagnosis
Demographic
Activity of systemic disease
Not applicable (no extracranial metastases)
PD
PR
SD

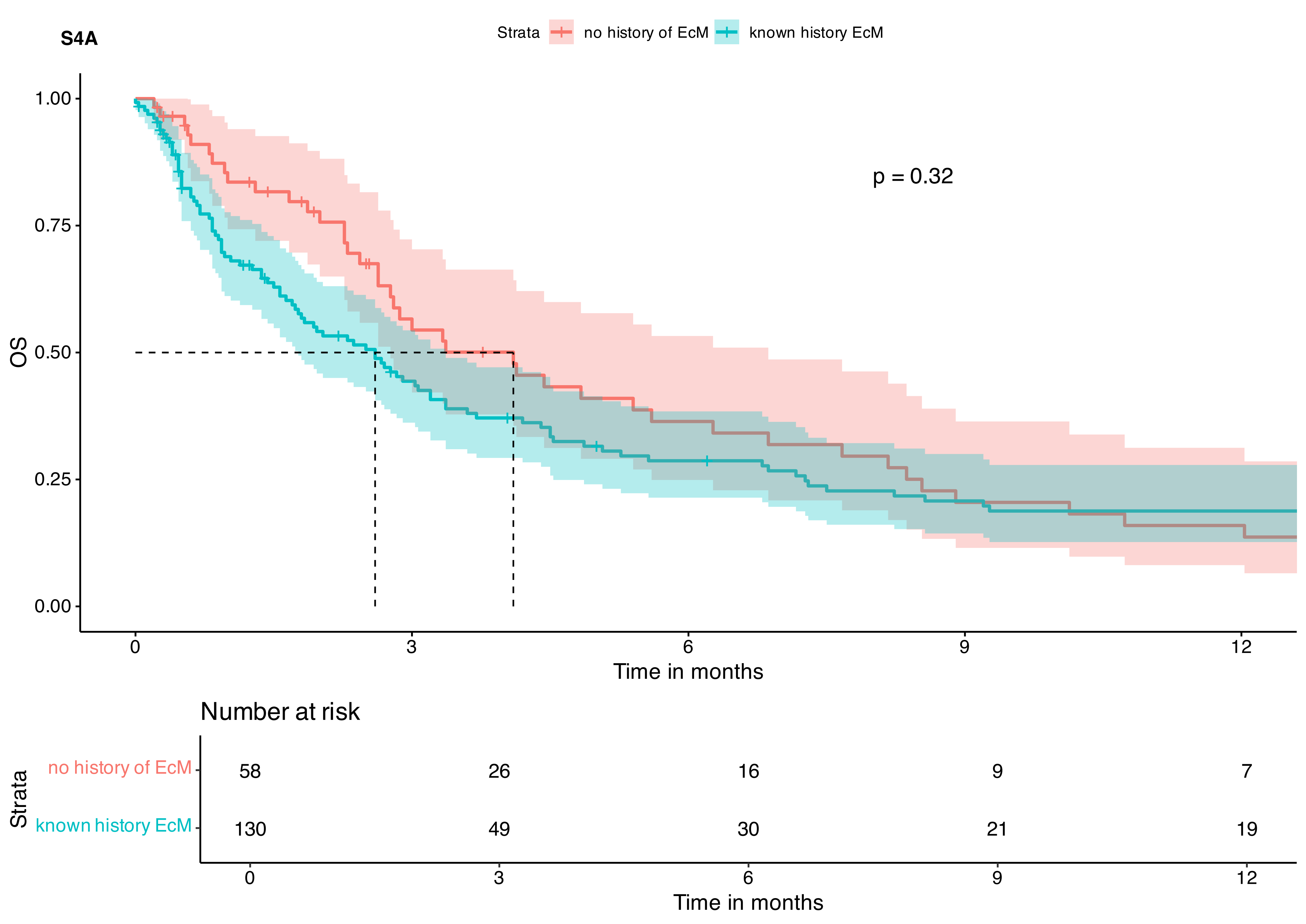

S4A
Strata
no history of EcM
known history EcM
p = 0.32
1.00
0.75
0.50
0.25
0.00
OS
0
3
6
9
12
Time in months
Number at risk
Strata
no history of EcM
known history EcM
58
26
16
9
7
130
49
30
21
19
0
3
6
9
12
Time in months

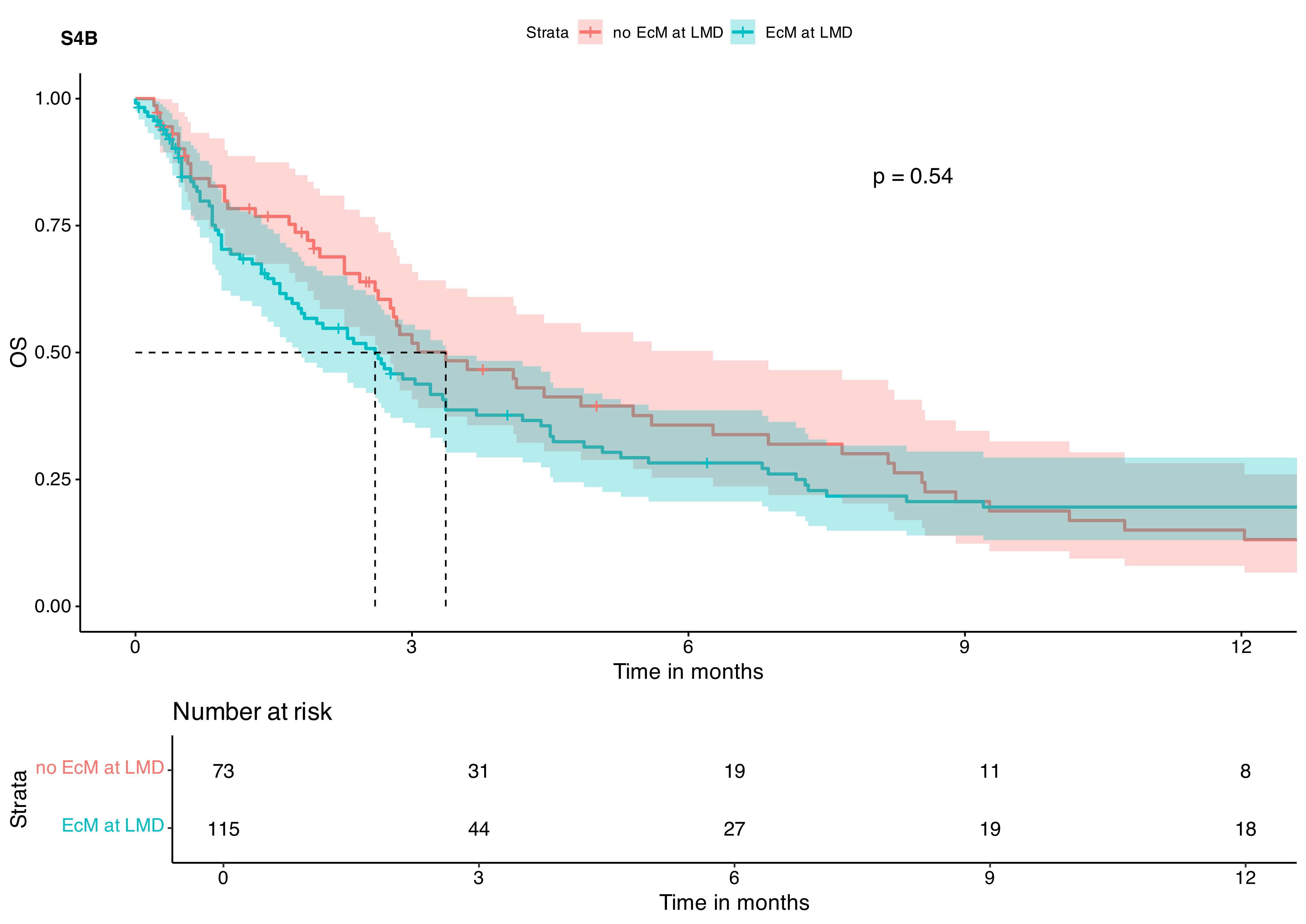

S4B
Strata
no EcM at LMD
EcM at LMD
p = 0.54
OS
1.00
0.75
0.50
0.25
0.00
0
3
6
9
12
Time in months
Number at risk
Strata
no EcM at LMD 73 31 19 11 8
EcM at LMD 115 44 27 19 18
Time in months

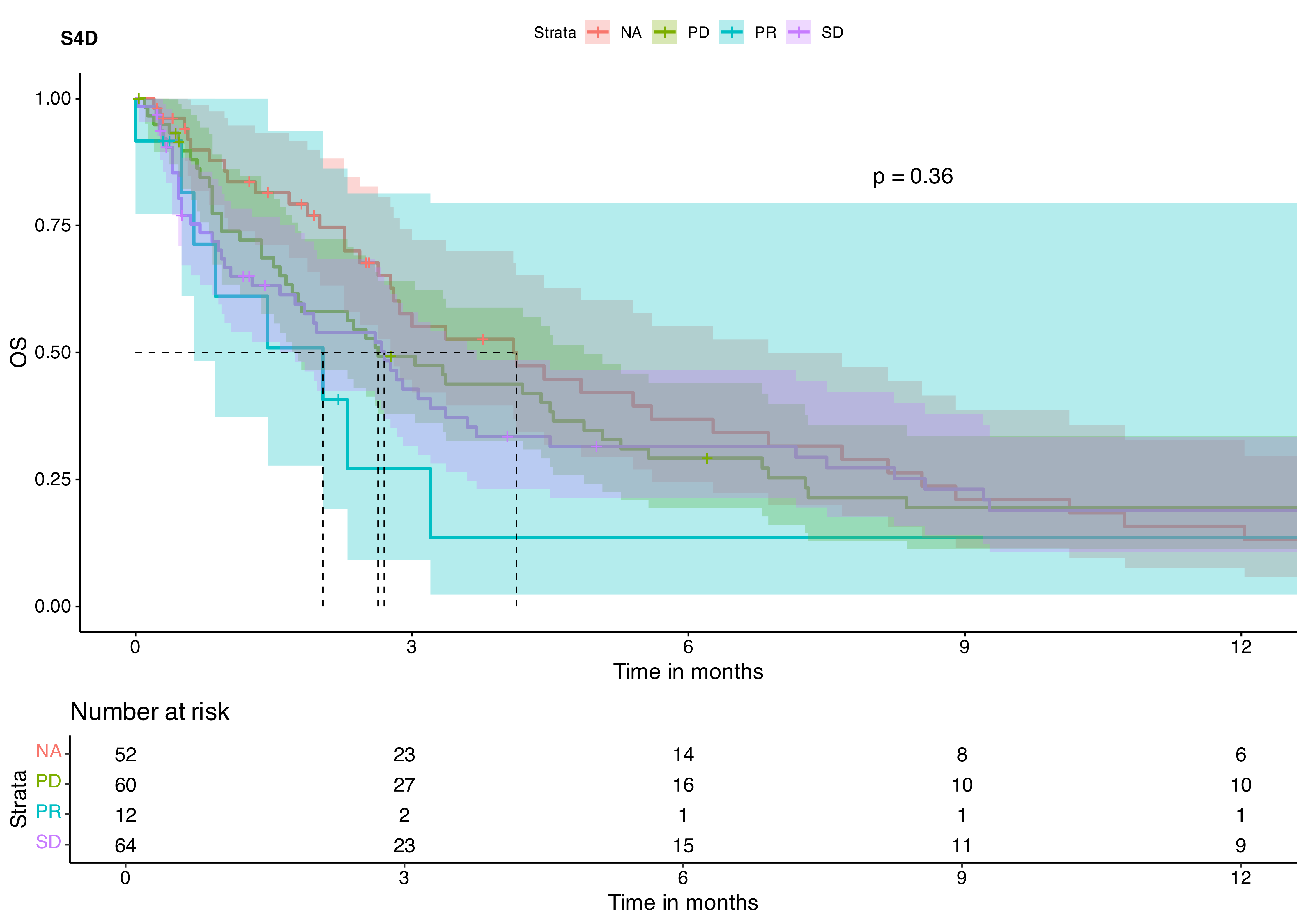

S4D
Strata NA PD PR SD
p = 0.36
OS
1.00
0.75
0.50
0.25
0.00
0
3
6
9
12
Time in months
Number at risk
Strata
NA 52 23 14 8 6
PD 60 27 16 10 10
PR 12 2 1 1 1
SD 64 23 15 11 9
0
3
6
9
12
Time in months

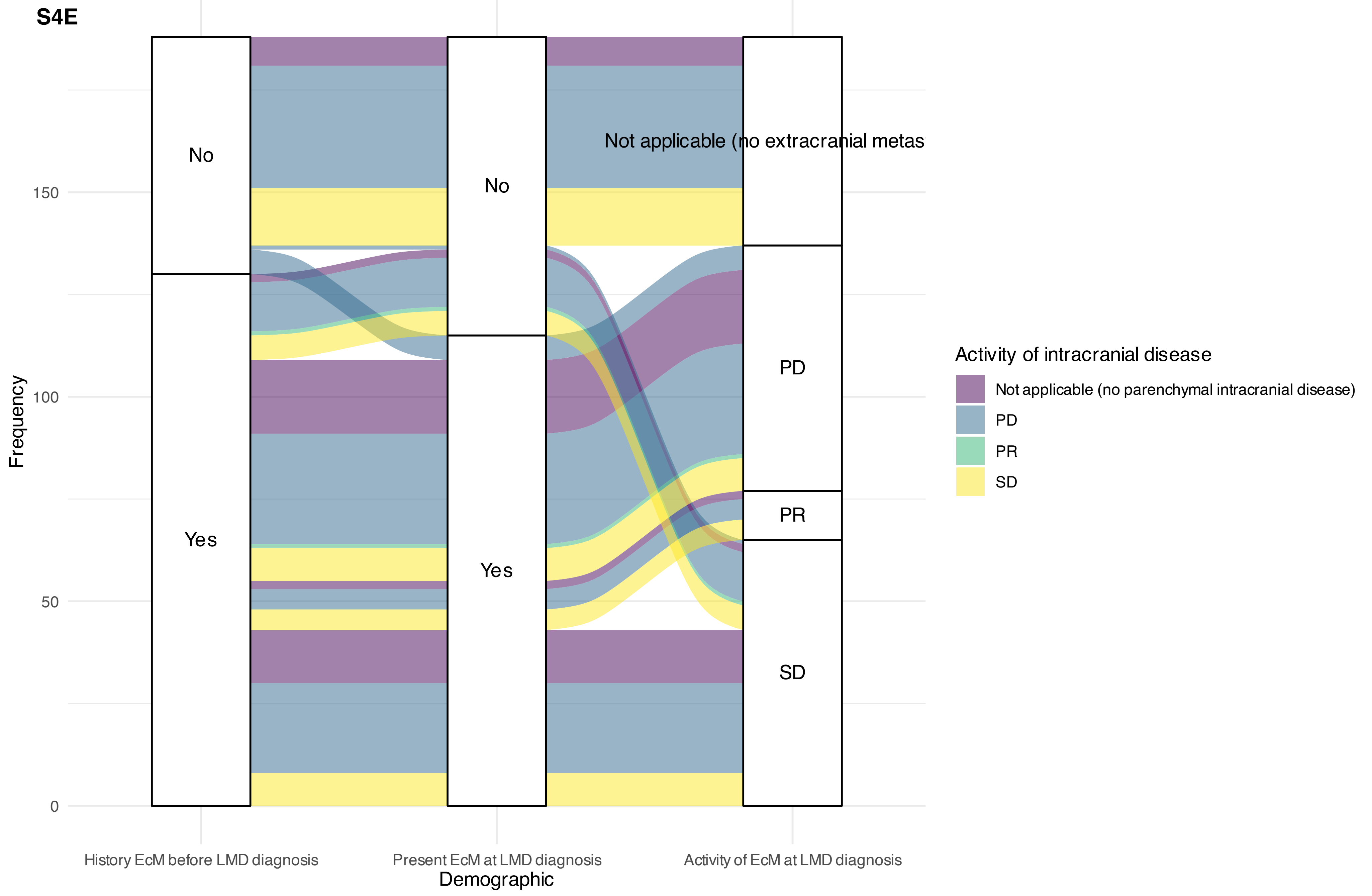

S4E
No
Yes
No
Yes
Not applicable (no extracranial metas
PD
PR
SD
150
100
50
0
Frequency
History EcM before LMD diagnosis
Present EcM at LMD diagnosis
Activity of EcM at LMD diagnosis
Demographic
Activity of intracranial disease
Not applicable (no parenchymal intracranial disease)
PD
PR
SD

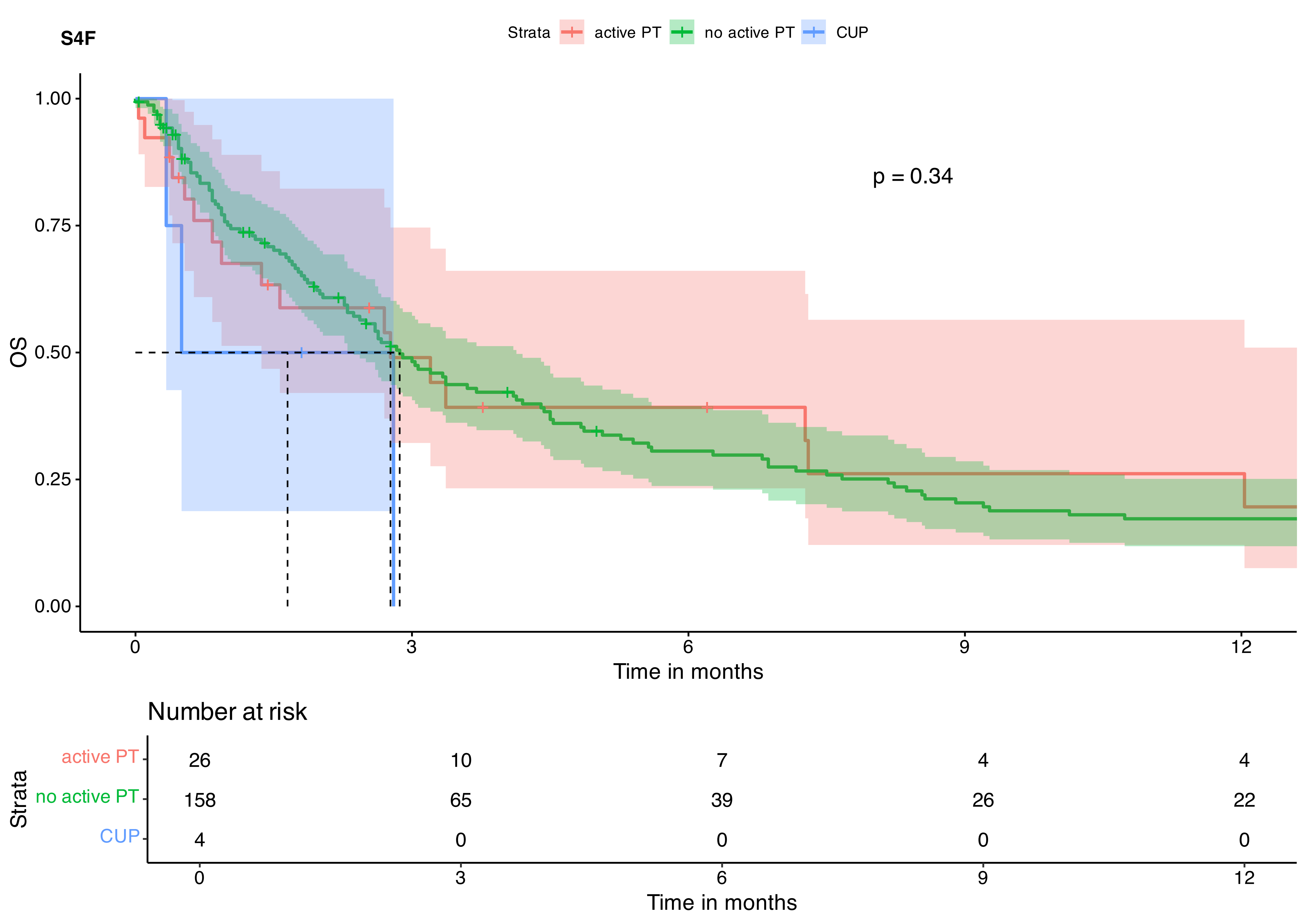
S4F
Strata
active PT
no active PT
CUP
p = 0.34
OS
1.00
0.75
0.50
0.25
0.00
0
3
6
9
12
Time in months
Number at risk
Strata
active PT 26 10 7 4 4
no active PT 158 65 39 26 22
CUP 4 0 0 0 0
0
3
6
9
12
Time in months
