# Supplementary material for: Clinical characteristics and outcomes in leptomeningeal disease with or without brain metastasis: insights from an explorative data analysis of the Charité LMD registry
Source: J Neurooncol. 2025 Feb 11;175(3):943–65. doi: 10.1007/s11060-025-04937-x (PMC12511199; doi:10.1007/s11060-025-04937-x)
Supplement: Supplementary file 4 — Supplementary file4 (DOCX 125 KB) [file 11060_2025_4937_MOESM4_ESM.docx]

Supplementary information

Clinical Characteristics and Outcomes in Leptomeningeal Disease with or without Brain Metastasis: Insights from an Explorative Data Analysis of the Charité LMD Registry

**David Wasilewski, Chiara Eitner, Rober Ates, Selin Murad, Zoe Shaked, Julia Alexandra Steinle, Andreas Wetzel-Yalelis, Tarik Alp Sargut, Judith Rösler, Majd Abdulhamid Samman, Peter Truckenmüller, Robert Mertens, Daniel Kroneberg, Alexander Kowski, Helena Radbruch, David Capper, Felix Ehret, Siyer Roohani, Nikolaj Frost, Jawed Nawabi, Julia Onken, Maximilian Schlaak, Jens-Uwe Blohmer, Uwe Pelzer, Ulrich Keller, Jalid Sehouli, Peter Vajkoczy, Ulrich Keilholz, Martin Misch**

**Supplementary figure 1: Patient selection and representative MRI findings of patient at LMD diagnosis**

CONSORTS diagram summarizing excluded and included patients in this study (**A**). Representative MRI images with a patient showing signs of classic LMD (cLMD) as evidenced by the presence of “sugar-coating”-like contrast-enhancing lesions near the cerebellar foliae (upper left); in the upper middle a cLMD involvement can be appreciated; extension of cLMD affecting the spinal cord with dural enhancement near the resection cavity (lower left); nodular LMD (nLMD) in a patient with previously resected perieto-occipital BrM (lower middle); cranial nerve involvement with an enhancement of near the meatus acusticus internus as a sign of cLMD (**B**).

**Supplementary figure 2: KM estimates for baseline demographic and baseline variables influencing OS**

KM survival curves for the total cohort with a median OS of 2.83 months [95% CI: 2.43 – 3.6] (**A**) and stratified according to tumor type (entity): breast cancer patients showed a median OS of 5.1 months [95% CI: 3.1 – 8.6] after LMD diagnosis, whereas melanoma patients showed a median OS of only 0.97 months [95% CI: 0.7 – 4.4], and patients with NSCLC a median OS of 2.6 months [95% CI: 1.7 – 3.2], and patient with other entities a median OS of 2.8 months [1.9 – 5.3], p=0.02 (**B**) or age group with patients dichotomized into ≥70 years of age vs. < 70 years of age (**C**), performance status group (e.g., Karnofsky Performance Scale) (**D**), presence of other chronic non-oncological diseases (i.e. cardiovascular disease, chronic respiratory diseases or chronic liver or kidney diseases) (**E**), type of LMD involvement (**F**), type I (evidence of tumor cells by means of CSF analysis) vs. type II (CSF negative cytology) (**F**), LMD presence of HCP at baseline (**G**).

**Supplementary figure 3: KM estimates for characteristics of intracranial parenchymal brain metastases in the context of LMD**

KM survival curves for patients grouped according to the history of presence of intracranial (parenchymal) BrM before LMD diagnosis, where patients with a history of previous BrM before LMD diagnosis showed a median OS of 2.8 months [95% CI: 2.3 – 3.6] vs. patients without a history of brain metastases before LMD diagnosis with a median OS of 3.3 months [95% CI: 1.9 – 6.9], p=0.24 (**A**), presence of intracranial (parenchymal) brain metastases at the timepoint of LMD diagnosis with patients without intracranial brain metastases at the time of LMD diagnosis showing a median OS of 2.6 months [95% CI: 1.9 – 6.9] vs. patients with presence of brain metastases at the time of LMD diagnosis with a median OS of 2.8 months [95% CI: 2.4 – 3.7], p=0.41 (**B**), activity of intracranial (parenchymal) brain metastases at the timepoint of LMD diagnosis (stable disease: SD, progressive disease: PD, partial response: PR, not applicable: NA (due to lack of intracranial (parenchymal) brain metastases) (**C**). An alluvial plot visualizes the transitions in patients with LMD across several dimensions, including history of parenchymal BrM before LMD diagnosis, presence of BrM at LMD diagnosis, activity of BrM at LMD diagnosis, and activity of systemic disease. The flow of patients is depicted from their initial status to their current status, highlighting the distribution and transitions among different conditions and activities related to brain and systemic metastases (**D**).

**Supplementary figure 4: KM estimates for characteristics of extracranial metastases and primary tumor activity in the context of LMD**

KM survival curves for patients grouped according to the history of presence EcM before LMD diagnosis where patients with a history of EcM showed a median OS of 2.6 months [95% CI: 1.8 – 3.4] vs. patients without a history of EcM showing a median OS of 4.1 months [95% CI: 2.8 – 6.9], p=0.32 (**A**), presence of EcM at the timepoint of LMD diagnosis in which patients without EcM at time of LMD diagnosis showed a median OS of 3.4 months [95% CI: 2.6 – 6.3] vs. patients with EcM at time of LMD diagnosis showing a median OS of 2.6 months [95%CI: 1.8 – 3.4], p=0.54, (**B**), activity of EcM at timepoint of LMD diagnosis (stable disease: SD, progressive disease: PD, partial response: PR, not applicable: NA (due to lack of intracranial (parenchymal) brain metastases) (**C**). Furthermore, patients were dichotomized into those with previous BrM before LMD diagnosis as well as the presence of BrM at diagnosis of LMD (median OS 2.8 months [95% CI: 2.3 – 3.4]) vs. patients without previous BrM and without the presence of BrM at diagnosis of LMD (median OS 4.2 months [95% CI: 1.9 – 8.9], p=0,29) (**D**). An alluvial plot visualizes the relationship between the history, presence, and activity of EcM in patients with LMD at the time of LMD diagnosis. It highlights the progression from patients' history of EcM before LMD diagnosis, the presence of EcM at LMD diagnosis, to the activity of EcM at LMD diagnosis, showing the distribution and flow of different states across these stages. Patients with an active primary tumor had a median overall survival (OS) of 3.20 months [95% CI: 2.70 – NA] compared to those with no active primary tumor showing a median OS of 2.83 months [95% CI: 2.30 – 3.70], and patients for whom this was not applicable (CUP) had a median OS of 1.65 months [95% CI: 0.333 – NA], p=0.34 (**E**).

| **Table 1a: General Patient Characteristics** | |
| --- | --- |
| **Variable** | **N = 188** |
| **Age, Median (IQR)** | 57.0 (48.0 – 66.9) |
| **Gender, n (%)** |  |
| Female | 120.0 (63.8) |
| Male | 68.0 (36.2) |
| **KPS group at the time of LMD diagnosis, n (%)** |  |
| 70% or less | 118 (63) |
| More than 70% | 70 (37) |
| **Presence of other underlying chronic diseases, n (%)** |  |
| No other underlying chronic diseases | 78.0 (41.5) |
| Other underlying chronic diseases | 110.0 (58.5) |
| **Headache present at diagnosis of LMD, n (%)** | 78.0 (41.5) |
| **Nausea present at diagnosis of LMD, n (%)** | 43.0 (22.9) |
| **Headache present at diagnosis of LMD, n (%)** | 24.0 (12.8) |
| **Vertigo present at diagnosis of LMD, n (%)** | 42.0 (22.3) |
| **Visual deficits present at diagnosis of LMD, n (%)** | 28.0 (14.9) |
| **Seizures present at diagnosis of LMD, n (%)** | 14.0 (7.4) |
| **Decrease in clinical performance present at diagnosis of LMD, n (%)** | 97.0 (51.6) |
| **Cranial nerve deficits present at diagnosis of LMD, n (%)** | 49.0 (26.1) |
| **LMD in the context of brain metastasis resection, n (%)** |  |
| LMD at time of brain metastasis resection | 21.0 (11.2) |
| No LMD before brain metastasis resection | 75.0 (39.9) |
| Not applicable (no brain metastasis resection performed) | 92.0 (48.9) |
| **Modality of LMD Diagnosis, n (%)** |  |
| LMD diagnosis based solely on MRI | 106.0 (56.4) |
| LMD diagnosis based solely on CSF sampling | 5.0 (2.7) |
| LMD diagnosis based solely on MRI and CSF sampling | 77.0 (41.0) |
| Dural biopsy | 0.0 (0.0) |
| **Dominant pattern of LMD, n (%)** |  |
| cLMD | 117.0 (64.6) |
| Mixed | 21.0 (11.6) |
| nLMD | 43.0 (23.8) |
| Unknown | 7 |
| **Presence of HCP at time of LMD diagnosis, n (%)** |  |
| HCP | 23.0 (12.2) |
| No HCP | 165.0 (87.8) |
| **EANO-ESMO subtype, n (%)** |  |
| Type IA | 66 (35) |
| Type IB | 15 (8.0) |
| Type IC | 8 (4.3) |
| Type ID | 2 (1.1) |
| Type IIA | 52 (28) |
| Type IIB | 28 (15) |
| Type IIC | 11 (5.9) |
| Type IID | 6 (3.2) |
| **Parenchymal brain metastases before LMD diagnosis, n (%)** |  |
| No history parenchymal brain metastases before LMD diagnosis | 69.0 (36.7) |
| History parenchymal brain metastases before LMD diagnosis | 119.0 (63.3) |
| **Parenchymal metastasis at time of LMD diagnosis, n (%)** |  |
| No parenchymal metastasis present at time of LMD diagnosis | 64.0 (34.0) |
| Parenchymal metastasis present at time of LMD diagnosis | 124.0 (66.0) |
| **Activity of intracranial parenchymal diseases at time of LMD diagnosis, n (%)** |  |
| Not applicable (no parenchymal intracranial disease) | 42.0 (22.3) |
| PD | 103.0 (54.8) |
| PR | 2.0 (1.1) |
| SD | 41.0 (21.8) |
| **Activity of primary tumor at time of LMD diagnosis, n (%)** |  |
| active primary tumor | 26.0 (13.8) |
| no active primary tumor | 158.0 (84.0) |
| not applicable (CUP) | 4.0 (2.1) |
| **Extracranial metastases before LMD diagnosis, n (%)** |  |
| No history extracranial metastases before LMD diagnosis | 58.0 (30.9) |
| History extracranial metastases before LMD diagnosis | 130.0 (69.1) |
| **Extracranial metastases at time of LMD diagnosis, n (%)** |  |
| No extracranial metastasis present at time of LMD diagnosis | 73.0 (38.8) |
| Extracranial metastasis present at time of LMD diagnosis | 115.0 (61.2) |
| **Activity of extracranial metastatic diseases at time of LMD diagnosis, n (%)** |  |
| Not applicable (no extracranial metastases) | 51.0 (27.1) |
| PD | 60.0 (31.9) |
| PR | 12.0 (6.4) |
| SD | 65.0 (34.6) |

**Supplementary table 1a: General patient characteristics,** including clinical characteristics and imaging-related patient features for all patients. Classic LMD (cLMD), nodular LMD (nLMD), LMD was classified into Type A: LM with typical linear MRI abnormalities; type B: LM with nodular disease; type C: LM with both linear and nodular disease; type D: LM without MRI abnormalities (except HCP) according to Le Rhun et and colleagues; Progressive disease (PD), partial response (PR), stable disease (SD) according to response evaluation criteria response assessment in neuro-oncology (RANO) criteria and response evaluation criteria in solid tumours (RECIST) criteria (version 1.1) for intracranial parenchymal metastasis and extracranial metastasis, respectively.

| **Table 1b: histopathological and biomarker-related patient characteristics** | |
| --- | --- |
| **Characteristic** | **N = 188** |
| **entity, n (%)** |  |
| Breast cancer | 64.0 (34.0) |
| Cervical cancer | 2.0 (1.1) |
| Colorectal cancer | 6.0 (3.2) |
| CUP | 4.0 (2.1) |
| Endometrial cancer | 2.0 (1.1) |
| Esophageal cancer | 5.0 (2.7) |
| Gastric cancer | 7.0 (3.7) |
| Head and neck cancer | 2.0 (1.1) |
| Hepatocellular carcinoma | 1.0 (0.5) |
| Leiomyosarcoma | 1.0 (0.5) |
| Melanoma | 27.0 (14.4) |
| Non-small cell lung cancer | 42.0 (22.3) |
| Ovarian cancer | 4.0 (2.1) |
| Pancreatic cancer | 2.0 (1.1) |
| Perivascular epithelioid cell tumor | 1.0 (0.5) |
| Prostate cancer | 3.0 (1.6) |
| Renal cell cancer | 6.0 (3.2) |
| Sinuonasal adenocarcinoma | 1.0 (0.5) |
| Small cell lung cancer | 5.0 (2.7) |
| Testicular cancer | 1.0 (0.5) |
| Urothelial cancer | 2.0 (1.1) |
| **targetable mutation or molecular alteration, n (%)** | 73.0 (38.8) |
| **Specific mutation or signaling pathway activated, n (%)** |  |
| ALK alteration (translocation or amplification) | 3.0 (1.6) |
| BRAF mut | 15.0 (8.0) |
| BRAF wt | 4.0 (2.1) |
| EGFR mutation | 6.0 (3.2) |
| Her2/NEU Dako score 0 | 21.0 (11.2) |
| Her2/NEU Dako score 1+ | 13.0 (6.9) |
| Her2/NEU Dako score 2+ | 4.0 (2.1) |
| Her2/NEU Dako score 3+ | 21.0 (11.2) |
| KRAS mut | 3.0 (1.6) |
| MET alteration (amplification, overexpression or exon skipping) | 5.0 (2.7) |
| NA | 6.0 (3.2) |
| None | 84.0 (44.7) |
| NRAS mut | 3.0 (1.6) |

**Supplementary table 1b:** Histopathological and biomarker-related patient characteristics for all patients.

| **Table 1c: Combined General Patient Characteristics** | |
| --- | --- |
| **Variable** | **N = 188** |
| **Neurosurgical brain metastasis resection, n (%)** |  |
| No brain metastasis resection | 92.0 (48.9) |
| Brain metastasis resection | 96.0 (51.1) |
| **Type of neurosurgical Intervention, n (%)** |  |
| No neurosurgical intervention | 69.0 (36.7) |
| Resection | 75.0 (39.9) |
| Resection and later reservoir placement | 10.0 (5.3) |
| Resection and later VP shunt placement | 3.0 (1.6) |
| Resection and reservoir placement | 3.0 (1.6) |
| Resection and VP shunt placement | 4.0 (2.1) |
| Resection and VP shunt placement and later reservoir placement | 1.0 (0.5) |
| Reservoir placement | 12.0 (6.4) |
| Reservoir placement and VP shunt placement | 1.0 (0.5) |
| VP shunt placement | 9.0 (4.8) |
| VP shunt placement and later reservoir placement | 1.0 (0.5) |
| **Presence of implanted reservoir, n (%)** | 29.0 (15.4) |
| **Timing of neurosurgical resection relative to LMD diagnosis, n (%)** |  |
| LMD at the time of brain metastasis resection | 21.0 (11.2) |
| No LMD before brain metastasis resection | 75.0 (39.9) |
| Not applicable (no brain metastasis resection performed) | 92.0 (48.9) |
| **OP brain metastasis before LMD diagnosis vs after, n (%)** |  |
| Not applicable | 95.0 (50.5) |
| OP after LMD diagnosis | 24.0 (12.8) |
| OP before LMD diagnosis | 69.0 (36.7) |
| **Number of brain metastasis resections, n (%)** |  |
| Five | 1.0 (0.5) |
| Four | 4.0 (2.1) |
| No neurosurgical brain metastasis resection | 92.0 (48.9) |
| One | 62.0 (33.0) |
| Three | 1.0 (0.5) |
| Two | 28.0 (14.9) |
| **Line of systemic treatment before LMD diagnosis, n (%)** |  |
| No systemic therapy (before LMD diagnosis) | 29.0 (15.4) |
| 1 therapy line | 85.0 (45.2) |
| 2 therapy lines | 33.0 (17.6) |
| 3 therapy lines | 21.0 (11.2) |
| 4 or more therapy lines | 20.0 (10.7) |
| **Systemic Treatment before LMD diagnosis, n (%)** |  |
| No systemic treatment before LMD diagnosis | 30.0 (16.0) |
| Systemic treatment before LMD diagnosis | 158.0 (84.0) |
| **Chemotherapy before LMD diagnosis, n (%)** |  |
| 5-Fluorouracil, Oxaliplatin, Docetxel | 1.0 (0.5) |
| 5-FU | 3.0 (1.6) |
| Adriamycin and cyclophosphamide followed by paclitaxel | 1.0 (0.5) |
| Capecitabine | 4.0 (2.1) |
| Carboplatin and etoposide | 4.0 (2.1) |
| Carboplatin and paclitaxel | 9.0 (4.8) |
| Carboplatin and pemetrexed | 3.0 (1.6) |
| Cisplatin | 2.0 (1.1) |
| Cisplatin and etoposide | 1.0 (0.5) |
| Cisplatin and gemcitabine | 1.0 (0.5) |
| Cisplatin and paclitaxel | 1.0 (0.5) |
| Cisplatin and pemetrexed | 4.0 (2.1) |
| Cisplatin and vinorelbine | 7.0 (3.7) |
| Cisplatin, doxorubicin and cyclophophamide | 2.0 (1.1) |
| Cisplatin, pemetrexed | 1.0 (0.5) |
| Dacarbazin, cisplatin and vindesin | 1.0 (0.5) |
| Dacarbazine | 3.0 (1.6) |
| Docetaxel | 2.0 (1.1) |
| Docetaxel, doxorubicine and cyclophosphamide | 1.0 (0.5) |
| Epirubicine and cyclophosphamide followed by paclitaxel | 38.0 (20.3) |
| FLOT | 7.0 (3.7) |
| Fluoruracil | 1.0 (0.5) |
| FOLFIRI | 2.0 (1.1) |
| FOLFOX | 4.0 (2.1) |
| Gemcitabine and cisplatin | 3.0 (1.6) |
| Gemcitabine and paclitaxel | 1.0 (0.5) |
| Gemicitabine and carboplatin | 1.0 (0.5) |
| No chemotherapy | 69.0 (36.9) |
| No targeted therapy | 1.0 (0.5) |
| Paclitaxel | 5.0 (2.7) |
| Paclitaxel and carboplatin | 1.0 (0.5) |
| Paclitaxel and ifosfamid | 2.0 (1.1) |
| Temozolomide and capecitabine | 1.0 (0.5) |
| Unknown | 1 |
| **Targeted therapy before LMD diagnosis, n (%)** |  |
| No targeted therapy | 117.0 (62.2) |
| Abemaciclib | 1.0 (0.5) |
| Afatinib | 1.0 (0.5) |
| Axcitinib | 1.0 (0.5) |
| Axitinib | 1.0 (0.5) |
| Bevacizumab | 13.0 (6.9) |
| Bevacizumab, Palbociclib | 1.0 (0.5) |
| Binimetinib | 1.0 (0.5) |
| Binimetinib and encorafinib | 1.0 (0.5) |
| Brigatinib | 1.0 (0.5) |
| Cabozatinib and sunitinib | 1.0 (0.5) |
| Crizotinib | 1.0 (0.5) |
| Enzalutamide | 1.0 (0.5) |
| Everolimus | 1.0 (0.5) |
| Lapatinib | 2.0 (1.1) |
| Nintedanib | 2.0 (1.1) |
| Osimertinib | 2.0 (1.1) |
| Palbociclib | 7.0 (3.7) |
| Pazpanib | 1.0 (0.5) |
| Ramucirumab | 3.0 (1.6) |
| Sacituzumab-Govitecan | 1.0 (0.5) |
| Sunitinib | 1.0 (0.5) |
| Sunitinib and pazopanib | 1.0 (0.5) |
| Trametinib | 1.0 (0.5) |
| Trametinib and dabrafenib | 7.0 (3.7) |
| Trastuzumab | 8.0 (4.3) |
| Trastuzumab and pertuzumab | 9.0 (4.8) |
| Vemurafenib | 1.0 (0.5) |
| **Checkpoint Inhibitors before LMD diagnosis, n (%)** |  |
| No immunotherapy | 146.0 (77.7) |
| Atezolizumab | 1.0 (0.5) |
| Avelumab | 1.0 (0.5) |
| Durvalumab | 1.0 (0.5) |
| Interferone | 1.0 (0.5) |
| Nivolumab | 9.0 (4.8) |
| Nivolumab and ipilimumab | 13.0 (6.9) |
| Pembrolizumab | 16.0 (8.5) |
| **Local treatment before LMD diagnosis, n (%)** |  |
| None | 84.0 (44.7) |
| Brain metastasis resection | 10.0 (5.3) |
| Brain metastasis resection and radiation therapy | 54.0 (28.7) |
| Radiation therapy | 29.0 (15.4) |
| Radiation therapy (SNUC) | 1.0 (0.5) |
| Radiation therapy (spine metastases) | 4.0 (2.1) |
| Surgery (spinal intervention) | 1.0 (0.5) |
| Surgery (spinal intervention) and radiation therapy (spine metastases) | 5.0 (2.7) |
| **Radiation therapy before LMD diagnosis, n (%)** |  |
| No radiation therapy | 97.0 (51.6) |
| Conventional radiation therapy | 13.0 (6.9) |
| Conventional radiation therapy (later WBRT) | 1.0 (0.5) |
| CSI | 2.0 (1.1) |
| SRS | 56.0 (29.8) |
| SRS (later WBRT) | 1.0 (0.5) |
| WBRT | 14.0 (7.4) |
| WBRT (later conventional radiation therapy) | 1.0 (0.5) |
| WBRT (later SRS) | 3.0 (1.6) |

**Table 1c:** Treatment-related patient characteristics before LMD diagnosis, including local surgical procedures such as microsurgical brain metastasis resection, ventriculoperitoneal (VP) shunt placement, reservoir placement, or a combination of these (in the context of made LMD diagnosis); serial treatment, i.e. VP shunt placement following resection is indicated in the table; systemic therapies were classified into chemotherapy, targeted therapy and immunotherapy; Craniospinal irradiation (CSI), stereotactic radiosurgery (SRS), whole brain radiation (WBRT). If sequential radiotherapy was performed this was indicated in the table (e.g., “SRS (later WBRT)”). Sequential VP shunt placement was performed due to the new onset of HCP in the course of the disease.

| **Table 1d: Treatment-Related Patient Characteristics After LMD Diagnosis** | |
| --- | --- |
| **Characteristic** | **N = 188** |
| **Reservoir implantation, n (%)** | 29.0 (15.4) |
| **intrathecal therapy after diagnosis of LMD, n (%)** |  |
| No i.t. therapy | 148.0 (78.7) |
| i.t. therapy via repetitive LP | 23.0 (12.2) |
| i.t. therapy via reservoir | 17.0 (9.0) |
| **CTx after diagnosis of LMD, n (%)** |  |
| No chemotherapy | 159.0 (84.6) |
| 5-FU | 2.0 (1.1) |
| Adriamycin, cyclophosphamide and vincristine | 1.0 (0.5) |
| Capecitabine | 7.0 (3.7) |
| Carboplatin | 1.0 (0.5) |
| Carboplatin and etoposide | 1.0 (0.5) |
| Carboplatin and nab-Paclitaxel | 3.0 (1.6) |
| Cisplatin | 2.0 (1.1) |
| Cisplatin and etoposide | 1.0 (0.5) |
| Cisplatin, pemetrexed and carboplatin | 1.0 (0.5) |
| Docetaxel | 1.0 (0.5) |
| Doxorubicin | 1.0 (0.5) |
| Doxorubicin and MTX | 1.0 (0.5) |
| FLOT | 1.0 (0.5) |
| FOLFIRI | 1.0 (0.5) |
| Gemcitabine | 1.0 (0.5) |
| Paclitaxel | 2.0 (1.1) |
| Topotecan | 1.0 (0.5) |
| Trifluridin and tipiracil | 1.0 (0.5) |
| **TKIs or mAbs after diagnosis of LMD, n (%)** |  |
| No targeted therapy | 155.0 (82.4) |
| Axitinib | 1.0 (0.5) |
| Bevacizumab | 2.0 (1.1) |
| Binimetinib | 1.0 (0.5) |
| Crizotinib | 2.0 (1.1) |
| Eribulin | 1.0 (0.5) |
| Erlotinib | 1.0 (0.5) |
| Lapatinib | 3.0 (1.6) |
| Nintedanib | 1.0 (0.5) |
| Olaparib | 1.0 (0.5) |
| Osimertinib | 2.0 (1.1) |
| Palbociclib | 1.0 (0.5) |
| Pazopanib | 1.0 (0.5) |
| Ramucirumab | 1.0 (0.5) |
| Sacituzumab-Govitecan | 3.0 (1.6) |
| Sorafenib | 1.0 (0.5) |
| Trametinib and dabrafenib | 3.0 (1.6) |
| Trastuzumab | 1.0 (0.5) |
| Trastuzumab-Deruxtecan | 1.0 (0.5) |
| Trastuzumab-Emtansin | 4.0 (2.1) |
| Trastuzumab-Emtansin and tucatinib | 2.0 (1.1) |
| **CPIs after diagnosis of LMD, n (%)** |  |
| No immunotherapy | 172.0 (91.5) |
| Atezolizumab | 1.0 (0.5) |
| Nivolumab | 2.0 (1.1) |
| Nivolumab, ipilimumab | 6.0 (3.2) |
| Pembrolizumab | 7.0 (3.7) |
| **Local therapies after diagnosis of LMD, n (%)** |  |
| None | 71.0 (37.8) |
| Brain metastasis resection | 7.0 (3.7) |
| Brain metastasis resection and later reservoir placement | 2.0 (1.1) |
| Brain metastasis resection and later VP shunt placement | 1.0 (0.5) |
| Brain metastasis resection and reservoir placement | 2.0 (1.1) |
| Brain metastasis resection and VP shunt placement | 2.0 (1.1) |
| Brain metastasis resection and VP shunt placement followed by WBRT | 1.0 (0.5) |
| Brain metastasis resection followed by conventional radiotherapy | 1.0 (0.5) |
| Brain metastasis resection followed by CSI | 2.0 (1.1) |
| Brain metastasis resection followed by WBRT | 7.0 (3.7) |
| Brain metastasis resection, reservoir placement followed by SRS | 1.0 (0.5) |
| Conventional radiation therapy | 5.0 (2.7) |
| CSI | 4.0 (2.1) |
| Reservoir placement | 13.0 (6.9) |
| Reservoir placement and followed by WBRT | 2.0 (1.1) |
| Reservoir placement and VP shunt placement | 1.0 (0.5) |
| Reservoir placement and WBRT | 1.0 (0.5) |
| Reservoir placement followed by SRS | 1.0 (0.5) |
| Reservoir placement followed by WBRT | 2.0 (1.1) |
| SRS | 7.0 (3.7) |
| Surgery (spinal intervention) | 2.0 (1.1) |
| Surgery and radiation therapy | 1.0 (0.5) |
| VP shunt placement followed by WBRT | 1.0 (0.5) |
| VP shunt placement | 7.0 (3.7) |
| VP shunt placement and later reservoir placement | 1.0 (0.5) |
| VP shunt placement and later reservoir placement followed by CSI | 1.0 (0.5) |
| VP shunt placement followed by WBRT | 2.0 (1.1) |
| WBRT | 40.0 (21.3) |
| **Irradiation after diagnosis of LMD, n (%)** |  |
| No radiation therapy | 109.0 (58.0) |
| Conventional radiation therapy | 7.0 (3.7) |
| CSI | 7.0 (3.7) |
| SRS | 9.0 (4.8) |
| WBRT | 56.0 (29.8) |

**Table 1d:** Treatment-Related Patient Characteristics After LMD Diagnosis for all patients, including local intrathecal (i.t.) methotrexate (MTX) via repetitive lumbar punctures or a Rickham reservoir, systemic therapies, or local radiation therapy. Craniospinal irradiation (CSI), stereotactic radiosurgery (SRS), whole brain radiation (WBRT).

| **Table 2a: General Patient Characteristics** | | | | | | |
| --- | --- | --- | --- | --- | --- | --- |
| **Variable** | **Breast cancer**, N = 64 | **Melanoma**, N = 27 | **Non-small cell lung cancer**, N = 42 | **Other**, N = 55 | **p-value***^1^* | **q-value***^2^* |
| **Age, Median (IQR)** | 51.1 (44.7 – 59.9) | 55.6 (46.4 – 67.1) | 65.4 (56.9 – 69.2) | 59.4 (50.1 – 67.8) | <0.001 | 0.003 |
| **Gender, n (%)** |  |  |  |  | <0.001 | <0.001 |
| Female | 63.0 (98.4) | 14.0 (51.9) | 19.0 (45.2) | 24.0 (43.6) |  |  |
| Male | 1.0 (1.6) | 13.0 (48.1) | 23.0 (54.8) | 31.0 (56.4) |  |  |
| **KPS, n (%)** |  |  |  |  | 0.80 | 0.84 |
| 70% or less | 42 (66) | 18 (67) | 24 (57) | 34 (62) |  |  |
| More than 70% | 22 (34) | 9 (33) | 18 (43) | 21 (38) |  |  |
| **Presence of other underlying chronic diseases, n (%)** |  |  |  |  | <0.001 | <0.001 |
| No other underlying chronic diseases | 37.0 (57.8) | 17.0 (63.0) | 6.0 (14.3) | 18.0 (32.7) |  |  |
| Other underlying chronic diseases | 27.0 (42.2) | 10.0 (37.0) | 36.0 (85.7) | 37.0 (67.3) |  |  |
| **Headache present at diagnosis of LMD, n (%)** | 28.0 (43.8) | 11.0 (40.7) | 17.0 (40.5) | 22.0 (40.0) | 0.98 | 0.98 |
| **Nausea present at diagnosis of LMD, n (%)** | 16.0 (25.0) | 7.0 (25.9) | 7.0 (16.7) | 13.0 (23.6) | 0.74 | 0.83 |
| **Headache present at diagnosis of LMD, n (%)** | 8.0 (12.5) | 6.0 (22.2) | 4.0 (9.5) | 6.0 (10.9) | 0.47 | 0.61 |
| **Vertigo present at diagnosis of LMD, n (%)** | 17.0 (26.6) | 6.0 (22.2) | 9.0 (21.4) | 10.0 (18.2) | 0.75 | 0.83 |
| **Visual deficits present at diagnosis of LMD, n (%)** | 13.0 (20.3) | 3.0 (11.1) | 6.0 (14.3) | 6.0 (10.9) | 0.51 | 0.63 |
| **Seizures present at diagnosis of LMD, n (%)** | 2.0 (3.1) | 5.0 (18.5) | 3.0 (7.1) | 4.0 (7.3) | 0.10 | 0.21 |
| **Decrease in clinical performance present at diagnosis of LMD, n (%)** | 32.0 (50.0) | 15.0 (55.6) | 26.0 (61.9) | 24.0 (43.6) | 0.33 | 0.54 |
| **Cranial nerve deficits present at diagnosis of LMD, n (%)** | 11.0 (17.2) | 5.0 (18.5) | 19.0 (45.2) | 14.0 (25.5) | 0.010 | 0.040 |
| **LMD in the context of brain metastasis resection, n (%)** |  |  |  |  | 0.42 | 0.59 |
| LMD at the time of brain metastasis resection | 10.0 (15.6) | 3.0 (11.1) | 3.0 (7.1) | 5.0 (9.1) |  |  |
| No LMD before brain metastasis resection | 24.0 (37.5) | 15.0 (55.6) | 17.0 (40.5) | 19.0 (34.5) |  |  |
| Not applicable (no brain metastasis resection performed) | 30.0 (46.9) | 9.0 (33.3) | 22.0 (52.4) | 31.0 (56.4) |  |  |
| **Modality of LMD Diagnosis, n (%)** |  |  |  |  | 0.071 | 0.19 |
| LMD diagnosis based solely on MRI | 29.0 (45.3) | 18.0 (66.7) | 30.0 (71.4) | 29.0 (52.7) |  |  |
| LMD diagnosis based solely on CSF sampling | 1.0 (1.6) | 1.0 (3.7) | 1.0 (2.4) | 2.0 (3.6) |  |  |
| LMD diagnosis based solely on MRI and CSF sampling | 34.0 (53.1) | 8.0 (29.6) | 11.0 (26.2) | 24.0 (43.6) |  |  |
| Dural biopsy | 0.0 (0.0) | 0.0 (0.0) | 0.0 (0.0) | 0.0 (0.0) |  |  |
| **Dominant pattern of LMD, n (%)** |  |  |  |  | 0.049 | 0.15 |
| cLMD | 47.0 (74.6) | 16.0 (61.5) | 22.0 (57.9) | 32.0 (59.3) |  |  |
| Mixed | 9.0 (14.3) | 3.0 (11.5) | 6.0 (15.8) | 3.0 (5.6) |  |  |
| nLMD | 7.0 (11.1) | 7.0 (26.9) | 10.0 (26.3) | 19.0 (35.2) |  |  |
| Unknown | 1 | 1 | 4 | 1 |  |  |
| **Presence of HCP at time of LMD diagnosis, n (%)** |  |  |  |  | 0.16 | 0.30 |
| HCP | 8.0 (12.5) | 6.0 (22.2) | 6.0 (14.3) | 3.0 (5.5) |  |  |
| No HCP | 56.0 (87.5) | 21.0 (77.8) | 36.0 (85.7) | 52.0 (94.5) |  |  |
| **EANO-ESMO subtype, n (%)** |  |  |  |  |  |  |
| Type IA | 29 (45) | 8 (30) | 9 (21) | 20 (36) |  |  |
| Type IB | 3 (4.7) | 2 (7.4) | 3 (7.1) | 7 (13) |  |  |
| Type IC | 5 (7.8) | 1 (3.7) | 2 (4.8) | 0 (0) |  |  |
| Type ID | 1 (1.6) | 0 (0) | 1 (2.4) | 0 (0) |  |  |
| Type IIA | 17 (27) | 8 (30) | 13 (31) | 14 (25) |  |  |
| Type IIB | 4 (6.3) | 5 (19) | 8 (19) | 11 (20) |  |  |
| Type IIC | 4 (6.3) | 2 (7.4) | 3 (7.1) | 2 (3.6) |  |  |
| Type IID | 1 (1.6) | 1 (3.7) | 3 (7.1) | 1 (1.8) |  |  |
| **Parenchymal brain metastases before LMD diagnosis, n (%)** |  |  |  |  | 0.023 | 0.081 |
| No history parenchymal brain metastases before LMD diagnosis | 21.0 (32.8) | 6.0 (22.2) | 13.0 (31.0) | 29.0 (52.7) |  |  |
| History parenchymal brain metastases before LMD diagnosis | 43.0 (67.2) | 21.0 (77.8) | 29.0 (69.0) | 26.0 (47.3) |  |  |
| **Parenchymal metastasis at the time of LMD diagnosis, n (%)** |  |  |  |  | 0.080 | 0.19 |
| No parenchymal metastasis present at the time of LMD diagnosis | 20.0 (31.3) | 6.0 (22.2) | 12.0 (28.6) | 26.0 (47.3) |  |  |
| Parenchymal metastasis present at the time of LMD diagnosis | 44.0 (68.8) | 21.0 (77.8) | 30.0 (71.4) | 29.0 (52.7) |  |  |
| **Activity of intracranial parenchymal diseases at the time of LMD diagnosis, n (%)** |  |  |  |  |  |  |
| Not applicable (no parenchymal intracranial disease) | 11.0 (17.2) | 4.0 (14.8) | 6.0 (14.3) | 21.0 (38.2) |  |  |
| PD | 33.0 (51.6) | 17.0 (63.0) | 26.0 (61.9) | 27.0 (49.1) |  |  |
| PR | 0.0 (0.0) | 1.0 (3.7) | 1.0 (2.4) | 0.0 (0.0) |  |  |
| SD | 20.0 (31.3) | 5.0 (18.5) | 9.0 (21.4) | 7.0 (12.7) |  |  |
| **Activity of primary tumor at the time of LMD diagnosis, n (%)** |  |  |  |  | 0.004 | 0.023 |
| active primary tumor | 5.0 (7.8) | 1.0 (3.7) | 7.0 (16.7) | 13.0 (23.6) |  |  |
| no active primary tumor | 59.0 (92.2) | 26.0 (96.3) | 35.0 (83.3) | 38.0 (69.1) |  |  |
| not applicable (CUP) | 0.0 (0.0) | 0.0 (0.0) | 0.0 (0.0) | 4.0 (7.3) |  |  |
| **Extracranial metastases before LMD diagnosis, n (%)** |  |  |  |  | 0.21 | 0.37 |
| No history extracranial metastases before LMD diagnosis | 20.0 (31.3) | 6.0 (22.2) | 18.0 (42.9) | 14.0 (25.5) |  |  |
| History extracranial metastases before LMD diagnosis | 44.0 (68.8) | 21.0 (77.8) | 24.0 (57.1) | 41.0 (74.5) |  |  |
| **Extracranial metastases at the time of LMD diagnosis, n (%)** |  |  |  |  | 0.37 | 0.56 |
| No extracranial metastasis present at the time of LMD diagnosis | 29.0 (45.3) | 12.0 (44.4) | 15.0 (35.7) | 17.0 (30.9) |  |  |
| Extracranial metastasis present at the time of LMD diagnosis | 35.0 (54.7) | 15.0 (55.6) | 27.0 (64.3) | 38.0 (69.1) |  |  |
| **Activity of extracranial metastatic diseases at the time of LMD diagnosis, n (%)** |  |  |  |  |  |  |
| Not applicable (no extracranial metastases) | 20.0 (31.3) | 5.0 (18.5) | 14.0 (33.3) | 12.0 (21.8) |  |  |
| PD | 17.0 (26.6) | 7.0 (25.9) | 13.0 (31.0) | 23.0 (41.8) |  |  |
| PR | 6.0 (9.4) | 2.0 (7.4) | 2.0 (4.8) | 2.0 (3.6) |  |  |
| SD | 21.0 (32.8) | 13.0 (48.1) | 13.0 (31.0) | 18.0 (32.7) |  |  |
| *^1^* Kruskal-Wallis rank sum test; Pearson’s Chi-squared test; Fisher’s exact test | | | | | | |
| *^2^* Benjamini & Hochberg correction for multiple testing | | | | | | |

**Supplementary table 2a: General patient characteristics, including clinical characteristics and imaging-related patient features grouped for the major tumor entities (i.e., breast cancer, NSCLC, melanoma) and other tumor entities.** Classic LMD (cLMD), nodular LMD (nLMD), LMD was classified into Type A: LM with typical linear MRI abnormalities; type B: LM with nodular disease; type C: LM with both linear and nodular disease; type D: LM without MRI abnormalities (except HCP) according to Le Rhun et and colleagues; Progressive disease (PD), partial response (PR), stable disease (SD) according to response evaluation criteria response assessment in neuro-oncology (RANO) criteria and response evaluation criteria in solid tumours (RECIST) criteria (version 1.1) for intracranial parenchymal metastasis and extracranial metastasis, respectively.

| **Variable** | **Breast cancer**, N = 64 | **Melanoma**, N = 27 | **Non-small cell lung cancer**, N = 42 | **Other**, N = 55 | **p-value***^1^* | **q-value***^2^* |
| --- | --- | --- | --- | --- | --- | --- |
| **Targetable mutation, n (%)** | 37.0 (57.8) | 17.0 (63.0) | 15.0 (35.7) | 4.0 (7.3) | <0.001 | <0.001 |
| **DAKO HER2/NEU, n (%)** |  |  |  |  |  |  |
| Her2/NEU Dako score 0 | 22.0 (34.4) | 0.0 (0.0) | 1.0 (2.4) | 2.0 (3.6) |  |  |
| Her2/NEU Dako score 1+ | 13.0 (20.3) | 0.0 (0.0) | 0.0 (0.0) | 0.0 (0.0) |  |  |
| Her2/NEU Dako score 2+ | 2.0 (3.1) | 0.0 (0.0) | 0.0 (0.0) | 0.0 (0.0) |  |  |
| Her2/NEU Dako score 3+ | 21.0 (32.8) | 0.0 (0.0) | 0.0 (0.0) | 0.0 (0.0) |  |  |
| NA | 6.0 (9.4) | 0.0 (0.0) | 0.0 (0.0) | 7.0 (12.7) |  |  |
| Not applicable (no breast cancer, no gastric cancer) | 0.0 (0.0) | 27.0 (100.0) | 41.0 (97.6) | 46.0 (83.6) |  |  |
| **Estrogen receptor, n (%)** |  |  |  |  |  |  |
| NA | 10.0 (15.6) | 0.0 (0.0) | 0.0 (0.0) | 0.0 (0.0) |  |  |
| Negative | 28.0 (43.8) | 0.0 (0.0) | 0.0 (0.0) | 0.0 (0.0) |  |  |
| Not applicable (no breast cancer, no endometrial cancer) | 0.0 (0.0) | 27.0 (100.0) | 42.0 (100.0) | 53.0 (96.4) |  |  |
| Positive | 26.0 (40.6) | 0.0 (0.0) | 0.0 (0.0) | 2.0 (3.6) |  |  |
| **Progesteron receptor, n (%)** |  |  |  |  |  |  |
| NA | 13.0 (20.3) | 0.0 (0.0) | 0.0 (0.0) | 1.0 (1.8) |  |  |
| Negative | 38.0 (59.4) | 0.0 (0.0) | 0.0 (0.0) | 1.0 (1.8) |  |  |
| Not applicable (no breast cancer, no endometrial cancer) | 0.0 (0.0) | 27.0 (100.0) | 42.0 (100.0) | 53.0 (96.4) |  |  |
| Positive | 13.0 (20.3) | 0.0 (0.0) | 0.0 (0.0) | 0.0 (0.0) |  |  |
| **LP1, n (%)** |  |  |  |  | 0.069 | 0.17 |
| No 1st LP | 18.0 (28.1) | 13.0 (48.1) | 12.0 (28.6) | 16.0 (29.1) |  |  |
| No tumor cells detected | 12.0 (18.8) | 6.0 (22.2) | 17.0 (40.5) | 15.0 (27.3) |  |  |
| Tumor cells detected | 34.0 (53.1) | 8.0 (29.6) | 13.0 (31.0) | 24.0 (43.6) |  |  |
| **LP2, n (%)** |  |  |  |  | 0.15 | 0.22 |
| No 2nd LP | 40.0 (62.5) | 20.0 (74.1) | 32.0 (76.2) | 42.0 (76.4) |  |  |
| No tumor cells detected | 9.0 (14.1) | 1.0 (3.7) | 7.0 (16.7) | 7.0 (12.7) |  |  |
| Tumor cells detected | 15.0 (23.4) | 6.0 (22.2) | 3.0 (7.1) | 6.0 (10.9) |  |  |
| **LP3, n (%)** |  |  |  |  | 0.58 | 0.58 |
| No 3rd LP | 53.0 (82.8) | 24.0 (88.9) | 36.0 (85.7) | 49.0 (89.1) |  |  |
| No tumor cells detected | 4.0 (6.3) | 1.0 (3.7) | 5.0 (11.9) | 3.0 (5.5) |  |  |
| Tumor cells detected | 7.0 (10.9) | 2.0 (7.4) | 1.0 (2.4) | 3.0 (5.5) |  |  |
| **EANO-ESMO LMD subtypes 1, n (%)** |  |  |  |  |  |  |
| Type IA | 29.0 (45.3) | 8.0 (29.6) | 9.0 (21.4) | 20.0 (36.4) |  |  |
| Type IB | 3.0 (4.7) | 2.0 (7.4) | 3.0 (7.1) | 7.0 (12.7) |  |  |
| Type IC | 5.0 (7.8) | 1.0 (3.7) | 2.0 (4.8) | 0.0 (0.0) |  |  |
| Type ID | 1.0 (1.6) | 0.0 (0.0) | 1.0 (2.4) | 0.0 (0.0) |  |  |
| Type IIA | 17.0 (26.6) | 8.0 (29.6) | 13.0 (31.0) | 14.0 (25.5) |  |  |
| Type IIB | 4.0 (6.3) | 5.0 (18.5) | 8.0 (19.0) | 11.0 (20.0) |  |  |
| Type IIC | 4.0 (6.3) | 2.0 (7.4) | 3.0 (7.1) | 2.0 (3.6) |  |  |
| Type IID | 1.0 (1.6) | 1.0 (3.7) | 3.0 (7.1) | 1.0 (1.8) |  |  |
| **EANO-ESMO LMD subtypes 2, n (%)** |  |  |  |  | 0.092 | 0.17 |
| Type I | 38.0 (59.4) | 11.0 (40.7) | 15.0 (35.7) | 27.0 (49.1) |  |  |
| Type II | 26.0 (40.6) | 16.0 (59.3) | 27.0 (64.3) | 28.0 (50.9) |  |  |
| **History of intracranial metastases, n (%)** |  |  |  |  | 0.023 | 0.084 |
| No history of intracranial metastases | 21.0 (32.8) | 6.0 (22.2) | 13.0 (31.0) | 29.0 (52.7) |  |  |
| History of intracranial metastases | 43.0 (67.2) | 21.0 (77.8) | 29.0 (69.0) | 26.0 (47.3) |  |  |
| **Intracranial metastasis present at the time of LMD diagnosis, n (%)** |  |  |  |  | 0.080 | 0.17 |
| No intracranial metastasis present | 20.0 (31.3) | 6.0 (22.2) | 12.0 (28.6) | 26.0 (47.3) |  |  |
| Intracranial metastasis present | 44.0 (68.8) | 21.0 (77.8) | 30.0 (71.4) | 29.0 (52.7) |  |  |
| **Active intracranial metastasis present at the time of LMD diagnosis, n (%)** |  |  |  |  |  |  |
| Progressive Disease | 33.0 (51.6) | 17.0 (63.0) | 26.0 (61.9) | 27.0 (49.1) |  |  |
| Stable Disease | 20.0 (31.3) | 5.0 (18.5) | 9.0 (21.4) | 7.0 (12.7) |  |  |
| Partial Response | 0.0 (0.0) | 1.0 (3.7) | 1.0 (2.4) | 0.0 (0.0) |  |  |
| Not Applicable | 11.0 (17.2) | 4.0 (14.8) | 6.0 (14.3) | 21.0 (38.2) |  |  |
| **History of extracranial metastases, n (%)** |  |  |  |  | 0.21 | 0.26 |
| No history of extracranial metastases | 20.0 (31.3) | 6.0 (22.2) | 18.0 (42.9) | 14.0 (25.5) |  |  |
| History of extracranial metastases | 44.0 (68.8) | 21.0 (77.8) | 24.0 (57.1) | 41.0 (74.5) |  |  |
| **Extracranial metastasis present at the time of LMD diagnosis, n (%)** |  |  |  |  | 0.37 | 0.41 |
| No extracranial metastasis present | 29.0 (45.3) | 12.0 (44.4) | 15.0 (35.7) | 17.0 (30.9) |  |  |
| Extracranial metastasis present | 35.0 (54.7) | 15.0 (55.6) | 27.0 (64.3) | 38.0 (69.1) |  |  |
| **Active extracranial metastasis present at the time of LMD diagnosis, n (%)** |  |  |  |  |  |  |
| Progressive Disease | 17.0 (26.6) | 7.0 (25.9) | 13.0 (31.0) | 23.0 (41.8) |  |  |
| Stable Disease | 21.0 (32.8) | 13.0 (48.1) | 13.0 (31.0) | 18.0 (32.7) |  |  |
| Partial Response | 6.0 (9.4) | 2.0 (7.4) | 2.0 (4.8) | 2.0 (3.6) |  |  |
| Not Applicable | 20.0 (31.3) | 5.0 (18.5) | 14.0 (33.3) | 12.0 (21.8) |  |  |
| **Activity of primary tumor mass at LMD diagnosis, n (%)** |  |  |  |  | 0.004 | 0.024 |
| No Active Primary Tumor | 59.0 (92.2) | 26.0 (96.3) | 35.0 (83.3) | 38.0 (69.1) |  |  |
| Active Primary Tumor | 5.0 (7.8) | 1.0 (3.7) | 7.0 (16.7) | 13.0 (23.6) |  |  |
| Not Applicable (CUP) | 0.0 (0.0) | 0.0 (0.0) | 0.0 (0.0) | 4.0 (7.3) |  |  |
| **HCP present at diagnosis of LMD, n (%)** |  |  |  |  | 0.16 | 0.22 |
| No HCP | 56.0 (87.5) | 21.0 (77.8) | 36.0 (85.7) | 52.0 (94.5) |  |  |
| HCP | 8.0 (12.5) | 6.0 (22.2) | 6.0 (14.3) | 3.0 (5.5) |  |  |
| *^1^* Pearson’s Chi-squared test; Fisher’s exact test | | | | | | |
| *^2^* Benjamini & Hochberg correction for multiple testing | | | | | | |

**Supplementary table 2b:** Pathological and pathoanatomical characteristics grouped for the major tumor entities (i.e. breast cancer, NSCLC, melanoma) and other tumor entities.

| **Variable** | **Breast cancer**, N = 64 | **Melanoma**, N = 27 | **Non-small cell lung cancer**, N = 42 | **Other**, N = 55 | **p-value***^1^* | **q-value***^2^* |
| --- | --- | --- | --- | --- | --- | --- |
| **Neurosurgical brain metastasis resection, n (%)** |  |  |  |  | 0.17 | 0.42 |
| No brain metastasis resection | 29.0 (45.3) | 9.0 (33.3) | 22.0 (52.4) | 32.0 (58.2) |  |  |
| Brain metastasis resection | 35.0 (54.7) | 18.0 (66.7) | 20.0 (47.6) | 23.0 (41.8) |  |  |
| **Reservoir implantaton, n (%)** | 12.0 (18.8) | 2.0 (7.4) | 5.0 (11.9) | 10.0 (18.2) | 0.49 | 0.49 |
| **Timing of neurosurgical resection relative to LMD diagnosis, n (%)** |  |  |  |  | 0.42 | 0.49 |
| LMD at time of brain metastasis resection | 10.0 (15.6) | 3.0 (11.1) | 3.0 (7.1) | 5.0 (9.1) |  |  |
| No LMD before brain metastasis resection | 24.0 (37.5) | 15.0 (55.6) | 17.0 (40.5) | 19.0 (34.5) |  |  |
| Not applicable (no brain metastasis resection performed) | 30.0 (46.9) | 9.0 (33.3) | 22.0 (52.4) | 31.0 (56.4) |  |  |
| **OP brain metastasis before LMD diagnosis vs after, n (%)** |  |  |  |  | 0.42 | 0.49 |
| Not applicable | 30.0 (46.9) | 10.0 (37.0) | 22.0 (52.4) | 33.0 (60.0) |  |  |
| OP after LMD diagnosis | 11.0 (17.2) | 3.0 (11.1) | 4.0 (9.5) | 6.0 (10.9) |  |  |
| OP before LMD diagnosis | 23.0 (35.9) | 14.0 (51.9) | 16.0 (38.1) | 16.0 (29.1) |  |  |
| **Number of brain metastasis resections, n (%)** |  |  |  |  |  |  |
| Five | 0.0 (0.0) | 1.0 (3.7) | 0.0 (0.0) | 0.0 (0.0) |  |  |
| Four | 1.0 (1.6) | 1.0 (3.7) | 1.0 (2.4) | 1.0 (1.8) |  |  |
| No neurosurgical brain metastasis resection | 30.0 (46.9) | 9.0 (33.3) | 22.0 (52.4) | 31.0 (56.4) |  |  |
| One | 25.0 (39.1) | 13.0 (48.1) | 9.0 (21.4) | 15.0 (27.3) |  |  |
| Three | 1.0 (1.6) | 0.0 (0.0) | 0.0 (0.0) | 0.0 (0.0) |  |  |
| Two | 7.0 (10.9) | 3.0 (11.1) | 10.0 (23.8) | 8.0 (14.5) |  |  |
| **Line of systemic treatment before LMD diagnosis, n (%)** |  |  |  |  |  |  |
| 1 therapy line | 23.0 (35.9) | 15.0 (55.6) | 18.0 (42.9) | 29.0 (52.7) |  |  |
| 2 therapy lines | 17.0 (26.6) | 3.0 (11.1) | 7.0 (16.7) | 6.0 (10.9) |  |  |
| 3 therapy lines | 8.0 (12.5) | 3.0 (11.1) | 4.0 (9.5) | 6.0 (10.9) |  |  |
| 4 or more therapy lines | 9.0 (14.1) | 1.0 (3.7) | 1.0 (2.4) | 4.0 (7.3) |  |  |
| 4 therapy lines | 1.0 (1.6) | 1.0 (3.7) | 0.0 (0.0) | 0.0 (0.0) |  |  |
| 6 therapy lines | 2.0 (3.1) | 0.0 (0.0) | 0.0 (0.0) | 0.0 (0.0) |  |  |
| 8 therapy lines | 1.0 (1.6) | 0.0 (0.0) | 0.0 (0.0) | 0.0 (0.0) |  |  |
| No systemic therapy (before LMD diagnosis) | 3.0 (4.7) | 4.0 (14.8) | 12.0 (28.6) | 10.0 (18.2) |  |  |
| **Systemic Treatment before LMD diagnosis, n (%)** |  |  |  |  | 0.005 | 0.023 |
| No systemic treatment before LMD diagnosis | 3.0 (4.7) | 4.0 (14.8) | 12.0 (28.6) | 11.0 (20.0) |  |  |
| Systemic treatment before LMD diagnosis | 61.0 (95.3) | 23.0 (85.2) | 30.0 (71.4) | 44.0 (80.0) |  |  |
| **Chemotherapy before LMD diagnosis, n (%)** |  |  |  |  |  |  |
| No chemotherapy | 9.0 (14.3) | 23.0 (85.2) | 19.0 (45.2) | 18.0 (32.7) |  |  |
| 5-Fluorouracil, Oxaliplatin, Docetxel | 0.0 (0.0) | 0.0 (0.0) | 0.0 (0.0) | 1.0 (1.8) |  |  |
| 5-FU | 0.0 (0.0) | 0.0 (0.0) | 1.0 (2.4) | 2.0 (3.6) |  |  |
| Adriamycin and cyclophosphamide, followed by paclitaxel | 1.0 (1.6) | 0.0 (0.0) | 0.0 (0.0) | 0.0 (0.0) |  |  |
| Capecitabine | 2.0 (3.2) | 0.0 (0.0) | 0.0 (0.0) | 2.0 (3.6) |  |  |
| Carboplatin and etoposide | 0.0 (0.0) | 0.0 (0.0) | 2.0 (4.8) | 2.0 (3.6) |  |  |
| Carboplatin and paclitaxel | 1.0 (1.6) | 0.0 (0.0) | 3.0 (7.1) | 5.0 (9.1) |  |  |
| Carboplatin and pemetrexed | 0.0 (0.0) | 0.0 (0.0) | 3.0 (7.1) | 0.0 (0.0) |  |  |
| Cisplatin | 1.0 (1.6) | 0.0 (0.0) | 1.0 (2.4) | 0.0 (0.0) |  |  |
| Cisplatin and etoposide | 0.0 (0.0) | 0.0 (0.0) | 0.0 (0.0) | 1.0 (1.8) |  |  |
| Cisplatin and gemcitabine | 0.0 (0.0) | 0.0 (0.0) | 1.0 (2.4) | 0.0 (0.0) |  |  |
| Cisplatin and paclitaxel | 0.0 (0.0) | 0.0 (0.0) | 0.0 (0.0) | 1.0 (1.8) |  |  |
| Cisplatin and pemetrexed | 0.0 (0.0) | 0.0 (0.0) | 4.0 (9.5) | 0.0 (0.0) |  |  |
| Cisplatin and vinorelbine | 0.0 (0.0) | 0.0 (0.0) | 6.0 (14.3) | 1.0 (1.8) |  |  |
| Cisplatin, doxorubicin and cyclophosphamide | 1.0 (1.6) | 0.0 (0.0) | 0.0 (0.0) | 1.0 (1.8) |  |  |
| Cisplatin, pemetrexed | 0.0 (0.0) | 0.0 (0.0) | 1.0 (2.4) | 0.0 (0.0) |  |  |
| Dacarbazin, cisplatin and vindesin | 0.0 (0.0) | 1.0 (3.7) | 0.0 (0.0) | 0.0 (0.0) |  |  |
| Dacarbazine | 0.0 (0.0) | 3.0 (11.1) | 0.0 (0.0) | 0.0 (0.0) |  |  |
| Docetaxel | 1.0 (1.6) | 0.0 (0.0) | 0.0 (0.0) | 1.0 (1.8) |  |  |
| Docetaxel, doxorubicine and cyclophosphamide | 1.0 (1.6) | 0.0 (0.0) | 0.0 (0.0) | 0.0 (0.0) |  |  |
| Epirubicine and cyclophosphamide, followed by paclitaxel | 38.0 (60.3) | 0.0 (0.0) | 0.0 (0.0) | 0.0 (0.0) |  |  |
| FLOT | 0.0 (0.0) | 0.0 (0.0) | 0.0 (0.0) | 7.0 (12.7) |  |  |
| Fluoruracil | 0.0 (0.0) | 0.0 (0.0) | 0.0 (0.0) | 1.0 (1.8) |  |  |
| FOLFIRI | 0.0 (0.0) | 0.0 (0.0) | 0.0 (0.0) | 2.0 (3.6) |  |  |
| FOLFOX | 2.0 (3.2) | 0.0 (0.0) | 0.0 (0.0) | 2.0 (3.6) |  |  |
| Gemcitabine and cisplatin | 0.0 (0.0) | 0.0 (0.0) | 0.0 (0.0) | 3.0 (5.5) |  |  |
| Gemcitabine and paclitaxel | 0.0 (0.0) | 0.0 (0.0) | 1.0 (2.4) | 0.0 (0.0) |  |  |
| Gemicitabine and carboplatin | 1.0 (1.6) | 0.0 (0.0) | 0.0 (0.0) | 0.0 (0.0) |  |  |
| No targeted therapy | 0.0 (0.0) | 0.0 (0.0) | 0.0 (0.0) | 1.0 (1.8) |  |  |
| Paclitaxel | 4.0 (6.3) | 0.0 (0.0) | 0.0 (0.0) | 1.0 (1.8) |  |  |
| Paclitaxel and carboplatin | 0.0 (0.0) | 0.0 (0.0) | 0.0 (0.0) | 1.0 (1.8) |  |  |
| Paclitaxel and ifosfamid | 1.0 (1.6) | 0.0 (0.0) | 0.0 (0.0) | 1.0 (1.8) |  |  |
| Temozolomide and capecitabine | 0.0 (0.0) | 0.0 (0.0) | 0.0 (0.0) | 1.0 (1.8) |  |  |
| Unknown | 1 | 0 | 0 | 0 |  |  |
| **Targeted therapy before LMD diagnosis, n (%)** |  |  |  |  |  |  |
| No targeted therapy | 32.0 (50.0) | 16.0 (59.3) | 32.0 (76.2) | 37.0 (67.3) |  |  |
| Abemaciclib | 1.0 (1.6) | 0.0 (0.0) | 0.0 (0.0) | 0.0 (0.0) |  |  |
| Afatinib | 0.0 (0.0) | 0.0 (0.0) | 1.0 (2.4) | 0.0 (0.0) |  |  |
| Axitinib | 0.0 (0.0) | 0.0 (0.0) | 0.0 (0.0) | 1.0 (1.8) |  |  |
| Axitinib | 0.0 (0.0) | 0.0 (0.0) | 0.0 (0.0) | 1.0 (1.8) |  |  |
| Bevacizumab | 4.0 (6.3) | 0.0 (0.0) | 2.0 (4.8) | 7.0 (12.7) |  |  |
| Bevacizumab, Palbociclib | 1.0 (1.6) | 0.0 (0.0) | 0.0 (0.0) | 0.0 (0.0) |  |  |
| Binimetinib | 0.0 (0.0) | 1.0 (3.7) | 0.0 (0.0) | 0.0 (0.0) |  |  |
| Binimetinib and encorafinib | 0.0 (0.0) | 1.0 (3.7) | 0.0 (0.0) | 0.0 (0.0) |  |  |
| Brigatinib | 0.0 (0.0) | 0.0 (0.0) | 1.0 (2.4) | 0.0 (0.0) |  |  |
| Cabozatinib and sunitinib | 0.0 (0.0) | 0.0 (0.0) | 0.0 (0.0) | 1.0 (1.8) |  |  |
| Crizotinib | 0.0 (0.0) | 0.0 (0.0) | 1.0 (2.4) | 0.0 (0.0) |  |  |
| Enzalutamide | 0.0 (0.0) | 0.0 (0.0) | 0.0 (0.0) | 1.0 (1.8) |  |  |
| Everolimus | 0.0 (0.0) | 0.0 (0.0) | 0.0 (0.0) | 1.0 (1.8) |  |  |
| Lapatinib | 2.0 (3.1) | 0.0 (0.0) | 0.0 (0.0) | 0.0 (0.0) |  |  |
| Nintedanib | 0.0 (0.0) | 0.0 (0.0) | 2.0 (4.8) | 0.0 (0.0) |  |  |
| Osimertinib | 0.0 (0.0) | 0.0 (0.0) | 2.0 (4.8) | 0.0 (0.0) |  |  |
| Palbociclib | 6.0 (9.4) | 0.0 (0.0) | 0.0 (0.0) | 1.0 (1.8) |  |  |
| Pazpanib | 0.0 (0.0) | 0.0 (0.0) | 0.0 (0.0) | 1.0 (1.8) |  |  |
| Ramucirumab | 0.0 (0.0) | 0.0 (0.0) | 1.0 (2.4) | 2.0 (3.6) |  |  |
| Sacituzumab-Govitecan | 1.0 (1.6) | 0.0 (0.0) | 0.0 (0.0) | 0.0 (0.0) |  |  |
| Sunitinib | 0.0 (0.0) | 0.0 (0.0) | 0.0 (0.0) | 1.0 (1.8) |  |  |
| Sunitinib and pazopanib | 0.0 (0.0) | 0.0 (0.0) | 0.0 (0.0) | 1.0 (1.8) |  |  |
| Trametinib | 0.0 (0.0) | 1.0 (3.7) | 0.0 (0.0) | 0.0 (0.0) |  |  |
| Trametinib and dabrafenib | 0.0 (0.0) | 7.0 (25.9) | 0.0 (0.0) | 0.0 (0.0) |  |  |
| Trastuzumab | 8.0 (12.5) | 0.0 (0.0) | 0.0 (0.0) | 0.0 (0.0) |  |  |
| Trastuzumab and pertuzumab | 9.0 (14.1) | 0.0 (0.0) | 0.0 (0.0) | 0.0 (0.0) |  |  |
| Vemurafenib | 0.0 (0.0) | 1.0 (3.7) | 0.0 (0.0) | 0.0 (0.0) |  |  |
| **Checkpoint Inhibitors before LMD diagnosis, n (%)** |  |  |  |  |  |  |
| No immunotherapy | 61.0 (95.3) | 7.0 (25.9) | 32.0 (76.2) | 46.0 (83.6) |  |  |
| Atezolizumab | 0.0 (0.0) | 0.0 (0.0) | 1.0 (2.4) | 0.0 (0.0) |  |  |
| Avelumab | 0.0 (0.0) | 0.0 (0.0) | 0.0 (0.0) | 1.0 (1.8) |  |  |
| Durvalumab | 0.0 (0.0) | 0.0 (0.0) | 0.0 (0.0) | 1.0 (1.8) |  |  |
| Interferon | 0.0 (0.0) | 1.0 (3.7) | 0.0 (0.0) | 0.0 (0.0) |  |  |
| Nivolumab | 0.0 (0.0) | 5.0 (18.5) | 1.0 (2.4) | 3.0 (5.5) |  |  |
| Nivolumab and ipilimumab | 0.0 (0.0) | 13.0 (48.1) | 0.0 (0.0) | 0.0 (0.0) |  |  |
| Pembrolizumab | 3.0 (4.7) | 1.0 (3.7) | 8.0 (19.0) | 4.0 (7.3) |  |  |
| **Local treatment before LMD diagnosis, n (%)** |  |  |  |  |  |  |
| None | 28.0 (43.8) | 8.0 (29.6) | 19.0 (45.2) | 29.0 (52.7) |  |  |
| Brain metastasis resection | 4.0 (6.3) | 2.0 (7.4) | 2.0 (4.8) | 2.0 (3.6) |  |  |
| Brain metastasis resection and radiation therapy | 17.0 (26.6) | 10.0 (37.0) | 14.0 (33.3) | 13.0 (23.6) |  |  |
| Radiation therapy | 9.0 (14.1) | 7.0 (25.9) | 7.0 (16.7) | 6.0 (10.9) |  |  |
| Radiation therapy (SNUC) | 0.0 (0.0) | 0.0 (0.0) | 0.0 (0.0) | 1.0 (1.8) |  |  |
| Radiation therapy (spine metastases) | 3.0 (4.7) | 0.0 (0.0) | 0.0 (0.0) | 1.0 (1.8) |  |  |
| Surgery (spinal intervention) | 0.0 (0.0) | 0.0 (0.0) | 0.0 (0.0) | 1.0 (1.8) |  |  |
| Surgery (spinal intervention) and radiation therapy (spine metastases) | 3.0 (4.7) | 0.0 (0.0) | 0.0 (0.0) | 2.0 (3.6) |  |  |
| **Radiation therapy before LMD diagnosis, n (%)** |  |  |  |  |  |  |
| No radiation therapy | 32.0 (50.0) | 10.0 (37.0) | 21.0 (50.0) | 34.0 (61.8) |  |  |
| Conventional radiation therapy | 5.0 (7.8) | 0.0 (0.0) | 4.0 (9.5) | 4.0 (7.3) |  |  |
| Conventional radiation therapy (later WBRT) | 0.0 (0.0) | 0.0 (0.0) | 1.0 (2.4) | 0.0 (0.0) |  |  |
| CSI | 2.0 (3.1) | 0.0 (0.0) | 0.0 (0.0) | 0.0 (0.0) |  |  |
| SRS | 19.0 (29.7) | 14.0 (51.9) | 12.0 (28.6) | 11.0 (20.0) |  |  |
| SRS (later WBRT) | 0.0 (0.0) | 1.0 (3.7) | 0.0 (0.0) | 0.0 (0.0) |  |  |
| WBRT | 4.0 (6.3) | 1.0 (3.7) | 4.0 (9.5) | 5.0 (9.1) |  |  |
| WBRT (later conventional radiation therapy) | 1.0 (1.6) | 0.0 (0.0) | 0.0 (0.0) | 0.0 (0.0) |  |  |
| WBRT (later SRS) | 1.0 (1.6) | 1.0 (3.7) | 0.0 (0.0) | 1.0 (1.8) |  |  |
| *^1^* Pearson’s Chi-squared test; Fisher’s exact test | | | | | | |
| *^2^* Benjamini & Hochberg correction for multiple testing | | | | | | |

**Supplementary table 2c:** Treatment-related patient characteristics before LMD diagnosis grouped for the major tumor entities (i.e. breast cancer, NSCLC, melanoma) and other tumor entities including local surgical procedures such as micorsurgical brain metastasis resection, ventriculoperitoneal (VP) shunt placement, reservoir placement or a combination of these (in context of made LMD diagnosis); serial treatment, i.e. VP shunt placement following resection is indicated in the table; systemic therapies were classified into chemotherapy, targeted therapy and immunotherapy; Craniospinal irradiation (CSI), stereotactic radiosurgery (SRS), whole brain radiation (WBRT). If sequential radiotherapy was performed this was indicated in the table (e.g. “SRS (later WBRT)”). Sequential VP shunt placement was performed due to signs of HCP.

| **Table 2d: Treatment-Related Characteristics After LMD Diagnosis** | | | | | | |
| --- | --- | --- | --- | --- | --- | --- |
| **Variable** | **Breast cancer**, N = 64 | **Melanoma**, N = 27 | **Non-small cell lung cancer**, N = 42 | **Other**, N = 55 | **p-value***^1^* | **q-value***^2^* |
| **History of intracranial metastases, n (%)** | 43 (67) | 21 (78) | 29 (69) | 26 (47) | 0.023 | 0.023 |
| **Intrathecal Therapy After Diagnosis of LMD, n (%)** |  |  |  |  | 0.014 | 0.020 |
| i.t. therapy via repetitive LP | 13.0 (20.3) | 0.0 (0.0) | 2.0 (4.8) | 8.0 (14.5) |  |  |
| i.t. therapy via reservoir | 6.0 (9.4) | 0.0 (0.0) | 4.0 (9.5) | 7.0 (12.7) |  |  |
| No i.t. therapy | 45.0 (70.3) | 27.0 (100.0) | 36.0 (85.7) | 40.0 (72.7) |  |  |
| **Chemotherapy After Diagnosis of LMD, n (%)** |  |  |  |  |  |  |
| No chemotherapy | 54.0 (84.4) | 27.0 (100.0) | 35.0 (83.3) | 43.0 (78.2) |  |  |
| 5-FU | 1.0 (1.6) | 0.0 (0.0) | 0.0 (0.0) | 1.0 (1.8) |  |  |
| Adriamycin, cyclophosphamide and vincristine | 0.0 (0.0) | 0.0 (0.0) | 0.0 (0.0) | 1.0 (1.8) |  |  |
| Capecitabine | 7.0 (10.9) | 0.0 (0.0) | 0.0 (0.0) | 0.0 (0.0) |  |  |
| Carboplatin | 0.0 (0.0) | 0.0 (0.0) | 1.0 (2.4) | 0.0 (0.0) |  |  |
| Carboplatin and etoposide | 0.0 (0.0) | 0.0 (0.0) | 0.0 (0.0) | 1.0 (1.8) |  |  |
| Carboplatin and nab-Paclitaxel | 0.0 (0.0) | 0.0 (0.0) | 2.0 (4.8) | 0.0 (0.0) |  |  |
| Carboplatin, paclitaxel | 0.0 (0.0) | 0.0 (0.0) | 1.0 (2.4) | 0.0 (0.0) |  |  |
| Cisplatin | 0.0 (0.0) | 0.0 (0.0) | 0.0 (0.0) | 2.0 (3.6) |  |  |
| Cisplatin and etoposide | 0.0 (0.0) | 0.0 (0.0) | 1.0 (2.4) | 0.0 (0.0) |  |  |
| Cisplatin, pemetrexed and carboplatin | 0.0 (0.0) | 0.0 (0.0) | 1.0 (2.4) | 0.0 (0.0) |  |  |
| Docetaxel | 0.0 (0.0) | 0.0 (0.0) | 1.0 (2.4) | 0.0 (0.0) |  |  |
| Doxorubicin | 0.0 (0.0) | 0.0 (0.0) | 0.0 (0.0) | 1.0 (1.8) |  |  |
| Doxorubicin and MTX | 0.0 (0.0) | 0.0 (0.0) | 0.0 (0.0) | 1.0 (1.8) |  |  |
| FLOT | 0.0 (0.0) | 0.0 (0.0) | 0.0 (0.0) | 1.0 (1.8) |  |  |
| FOLFIRI | 0.0 (0.0) | 0.0 (0.0) | 0.0 (0.0) | 1.0 (1.8) |  |  |
| Gemcitabine | 1.0 (1.6) | 0.0 (0.0) | 0.0 (0.0) | 0.0 (0.0) |  |  |
| Paclitaxel | 1.0 (1.6) | 0.0 (0.0) | 0.0 (0.0) | 1.0 (1.8) |  |  |
| Topotecan | 0.0 (0.0) | 0.0 (0.0) | 0.0 (0.0) | 1.0 (1.8) |  |  |
| Trifluridin and tipiracil | 0.0 (0.0) | 0.0 (0.0) | 0.0 (0.0) | 1.0 (1.8) |  |  |
| **Targeted Therapy After Diagnosis of LMD, n (%)** |  |  |  |  |  |  |
| No targeted therapy | 47.0 (73.4) | 23.0 (85.2) | 35.0 (83.3) | 50.0 (90.9) |  |  |
| Axitinib | 0.0 (0.0) | 0.0 (0.0) | 0.0 (0.0) | 1.0 (1.8) |  |  |
| Bevacizumab | 1.0 (1.6) | 0.0 (0.0) | 1.0 (2.4) | 0.0 (0.0) |  |  |
| Binimetinib | 0.0 (0.0) | 1.0 (3.7) | 0.0 (0.0) | 0.0 (0.0) |  |  |
| Crizotinib | 0.0 (0.0) | 0.0 (0.0) | 2.0 (4.8) | 0.0 (0.0) |  |  |
| Eribulin | 1.0 (1.6) | 0.0 (0.0) | 0.0 (0.0) | 0.0 (0.0) |  |  |
| Erlotinib | 0.0 (0.0) | 0.0 (0.0) | 1.0 (2.4) | 0.0 (0.0) |  |  |
| Lapatinib | 3.0 (4.7) | 0.0 (0.0) | 0.0 (0.0) | 0.0 (0.0) |  |  |
| Nintedanib | 0.0 (0.0) | 0.0 (0.0) | 1.0 (2.4) | 0.0 (0.0) |  |  |
| Olaparib | 1.0 (1.6) | 0.0 (0.0) | 0.0 (0.0) | 0.0 (0.0) |  |  |
| Osimertinib | 0.0 (0.0) | 0.0 (0.0) | 2.0 (4.8) | 0.0 (0.0) |  |  |
| Palbociclib | 1.0 (1.6) | 0.0 (0.0) | 0.0 (0.0) | 0.0 (0.0) |  |  |
| Pazopanib | 0.0 (0.0) | 0.0 (0.0) | 0.0 (0.0) | 1.0 (1.8) |  |  |
| Ramucirumab | 0.0 (0.0) | 0.0 (0.0) | 0.0 (0.0) | 1.0 (1.8) |  |  |
| Sacituzumab-Govitecan | 2.0 (3.1) | 0.0 (0.0) | 0.0 (0.0) | 1.0 (1.8) |  |  |
| Sorafenib | 0.0 (0.0) | 0.0 (0.0) | 0.0 (0.0) | 1.0 (1.8) |  |  |
| Trametinib and dabrafenib | 0.0 (0.0) | 3.0 (11.1) | 0.0 (0.0) | 0.0 (0.0) |  |  |
| Trastuzumab | 1.0 (1.6) | 0.0 (0.0) | 0.0 (0.0) | 0.0 (0.0) |  |  |
| Trastuzumab-Deruxtecan | 1.0 (1.6) | 0.0 (0.0) | 0.0 (0.0) | 0.0 (0.0) |  |  |
| Trastuzumab-Emtansin | 4.0 (6.3) | 0.0 (0.0) | 0.0 (0.0) | 0.0 (0.0) |  |  |
| Trastuzumab-Emtansin and tucatinib | 2.0 (3.1) | 0.0 (0.0) | 0.0 (0.0) | 0.0 (0.0) |  |  |
| **Checkpoint Inhibitors After Diagnosis of LMD, n (%)** |  |  |  |  | <0.001 | <0.001 |
| No immunotherapy | 63.0 (98.4) | 20.0 (74.1) | 39.0 (92.9) | 50.0 (90.9) |  |  |
| Atezolizumab | 0.0 (0.0) | 0.0 (0.0) | 1.0 (2.4) | 0.0 (0.0) |  |  |
| Nivolumab | 0.0 (0.0) | 2.0 (7.4) | 0.0 (0.0) | 0.0 (0.0) |  |  |
| Nivolumab, ipilimumab | 0.0 (0.0) | 5.0 (18.5) | 0.0 (0.0) | 1.0 (1.8) |  |  |
| Pembrolizumab | 1.0 (1.6) | 0.0 (0.0) | 2.0 (4.8) | 4.0 (7.3) |  |  |
| **Local Therapies After Diagnosis of LMD, n (%)** |  |  |  |  |  |  |
| Brain metastasis resection | 4.0 (6.3) | 1.0 (3.7) | 0.0 (0.0) | 2.0 (3.6) |  |  |
| Brain metastasis resection and later reservoir placement | 1.0 (1.6) | 0.0 (0.0) | 1.0 (2.4) | 0.0 (0.0) |  |  |
| Brain metastasis resection and later VP shunt placement | 1.0 (1.6) | 0.0 (0.0) | 0.0 (0.0) | 0.0 (0.0) |  |  |
| Brain metastasis resection and reservoir placement | 0.0 (0.0) | 1.0 (3.7) | 0.0 (0.0) | 1.0 (1.8) |  |  |
| Brain metastasis resection and VP shunt placement | 2.0 (3.1) | 0.0 (0.0) | 0.0 (0.0) | 0.0 (0.0) |  |  |
| Brain metastasis resection and VP shunt placement followed by WBRT | 1.0 (1.6) | 0.0 (0.0) | 0.0 (0.0) | 0.0 (0.0) |  |  |
| Brain metastasis resection followed by conventional radiotherapy | 0.0 (0.0) | 0.0 (0.0) | 0.0 (0.0) | 1.0 (1.8) |  |  |
| Brain metastasis resection followed by CSI | 1.0 (1.6) | 0.0 (0.0) | 1.0 (2.4) | 0.0 (0.0) |  |  |
| Brain metastasis resection followed by WBRT | 3.0 (4.7) | 2.0 (7.4) | 1.0 (2.4) | 1.0 (1.8) |  |  |
| Brain metastasis resection, reservoir placement followed by SRS | 0.0 (0.0) | 0.0 (0.0) | 1.0 (2.4) | 0.0 (0.0) |  |  |
| Conventional radiation therapy | 2.0 (3.1) | 1.0 (3.7) | 0.0 (0.0) | 2.0 (3.6) |  |  |
| CSI | 1.0 (1.6) | 1.0 (3.7) | 1.0 (2.4) | 1.0 (1.8) |  |  |
| None | 23.0 (35.9) | 11.0 (40.7) | 19.0 (45.2) | 18.0 (32.7) |  |  |
| Reservoir placement | 3.0 (4.7) | 0.0 (0.0) | 2.0 (4.8) | 8.0 (14.5) |  |  |
| Reservoir placement and followed by WBRT | 1.0 (1.6) | 0.0 (0.0) | 0.0 (0.0) | 1.0 (1.8) |  |  |
| Reservoir placement and VP shunt placement | 1.0 (1.6) | 0.0 (0.0) | 0.0 (0.0) | 0.0 (0.0) |  |  |
| Reservoir placement and WBRT | 0.0 (0.0) | 1.0 (3.7) | 0.0 (0.0) | 0.0 (0.0) |  |  |
| Reservoir placement followed by SRS | 1.0 (1.6) | 0.0 (0.0) | 0.0 (0.0) | 0.0 (0.0) |  |  |
| Reservoir placement followed by WBRT | 1.0 (1.6) | 0.0 (0.0) | 1.0 (2.4) | 0.0 (0.0) |  |  |
| SRS | 3.0 (4.7) | 1.0 (3.7) | 2.0 (4.8) | 1.0 (1.8) |  |  |
| Surgery (spinal intervention) | 0.0 (0.0) | 0.0 (0.0) | 1.0 (2.4) | 1.0 (1.8) |  |  |
| Surgery and radiation therapy | 0.0 (0.0) | 0.0 (0.0) | 0.0 (0.0) | 1.0 (1.8) |  |  |
| VP shunt placemement followed by WBRT | 1.0 (1.6) | 0.0 (0.0) | 0.0 (0.0) | 0.0 (0.0) |  |  |
| VP shunt placement | 2.0 (3.1) | 2.0 (7.4) | 3.0 (7.1) | 0.0 (0.0) |  |  |
| VP shunt placement and later reservoir placement | 1.0 (1.6) | 0.0 (0.0) | 0.0 (0.0) | 0.0 (0.0) |  |  |
| VP shunt placement and later reservoir placement followed by CSI | 1.0 (1.6) | 0.0 (0.0) | 0.0 (0.0) | 0.0 (0.0) |  |  |
| VP shunt placement followed by WBRT | 0.0 (0.0) | 1.0 (3.7) | 1.0 (2.4) | 0.0 (0.0) |  |  |
| WBRT | 10.0 (15.6) | 5.0 (18.5) | 8.0 (19.0) | 17.0 (30.9) |  |  |
| **Radiation Therapy After Diagnosis of LMD, n (%)** |  |  |  |  |  |  |
| Conventional radiation therapy | 2.0 (3.1) | 1.0 (3.7) | 0.0 (0.0) | 4.0 (7.3) |  |  |
| CSI | 3.0 (4.7) | 1.0 (3.7) | 2.0 (4.8) | 1.0 (1.8) |  |  |
| No radiation therapy | 38.0 (59.4) | 15.0 (55.6) | 26.0 (61.9) | 30.0 (54.5) |  |  |
| SRS | 4.0 (6.3) | 1.0 (3.7) | 3.0 (7.1) | 1.0 (1.8) |  |  |
| WBRT | 17.0 (26.6) | 9.0 (33.3) | 11.0 (26.2) | 19.0 (34.5) |  |  |
| *^1^* Pearson’s Chi-squared test; Fisher’s exact test | | | | | | |
| *^2^* Benjamini & Hochberg correction for multiple testing | | | | | | |

**Supplementary table 2d:** Treatment-Related Patient Characteristics After LMD Diagnosis grouped according to entities including local intrathecal (i.t.) methotrexate (MTX) via repetitive lumbar punctures or a Rickham reservoir, systemic therapies or local radiation therapy. Craniospinal irradiation (CSI), stereotactic radiosurgery (SRS), whole brain radiation (WBRT).
